# Transcriptional control of *C. albicans* white-opaque switching and modulation by environmental cues and strain background

**DOI:** 10.1128/mbio.00581-25

**Published:** 2025-04-09

**Authors:** Anupam Sharma, Ameen Homayoon, Michael Weyler, Corey Frazer, Bernardo Ramírez-Zavala, Joachim Morschhäuser, Richard J. Bennett

**Affiliations:** 1 Department of Molecular and Microbiology and Immunology, Brown University 368393 , Providence, Rhode Island, USA; 2 Institute of Molecular Infection Biology, University of Würzburg 9190, Würzburg, Bavaria, Germany; Duke University School of Medicine, Durham, North Carolina, USA

**Keywords:** transcription factors, phenotypic switching, strain specificity, media specificity

## Abstract

The opportunistic fungal pathogen *Candida albicans* can undergo cellular transitions in response to environmental cues that impact its lifestyle and its interactions with the human host. This is exemplified by the white-opaque switch, which is a heritable transition between two phenotypic states that is regulated by a highly interconnected network of transcription factors (TFs). To obtain greater understanding of the transcriptional regulation of the switch, we generated a genome-wide, tetracycline-inducible TF library in the WO-1 strain background and identified those TFs whose forced expression induces white cells to switch to the opaque state. This set of opaque-inducing TFs was also evaluated for their ability to induce switching in a second strain background, that of the standard reference strain SC5314, as well as during growth on different laboratory media. These experiments identify 14 TFs that can drive white-to-opaque switching when overexpressed but that do so in a highly strain- and media-specific manner. In particular, changes in pH, amino acids, and zinc concentrations had marked effects on the ability of TFs to drive phenotypic switching. These results provide insights into the complex transcriptional regulation of switching in *C. albicans* and reveal that an interplay between genetic and environmental factors determines TF function and cell fate.

**IMPORTANCE:**

The white-opaque switch in *Candida albicans* represents a model system for understanding an epigenetic switch in a eukaryotic pathogen. Here, we generated an inducible library of the set of transcription factors (TFs) present in *C. albicans* and identify 14 TFs that can drive the white-to-opaque transition when ectopically expressed. We demonstrate that several of these TFs induce the switch in a highly strain- and media-specific manner. This highlights that both strain background and changes in experimental conditions (including different water sources) can profoundly impact the phenotypic consequences of TF overexpression. Moreover, the inducible TF library provides an invaluable tool for the further analysis of TF function in this important human pathogen.

## INTRODUCTION

Cell differentiation is intrinsic to the development of multicellular organisms but is also important for the lifestyle of unicellular microbes. Examples of microbial cell differentiation are the formation of endospores in gram-positive bacteria (1) or the formation of stalk and swarmer cells in *Caulobacter crescentus* (2). Cellular differentiation is also crucial for pathogenic microorganisms such as Chlamydiae that change between a reticulate body capable of intracellular replication and an infectious elementary body that is transmitted between hosts (3). Dimorphic fungi similarly adopt different morphologies, with both the yeast form and the filamentous mold form having important consequences for pathogenesis in the host (4). Protozoal parasites have even more complex life cycles and go through multiple developmental stages during infection of humans and their insect vectors (5).

Cell differentiation events are also integral to the lifestyle of *Candida albicans,* a fungus that lives as a commensal on the mucosal surfaces of the gastrointestinal and urogenital tracts in healthy individuals. This fungus can switch between growing as a budding yeast and growing as filamentous hyphae or pseudohyphae (6). This transition facilitates invasion into host tissues and promotes symptomatic mucosal infections as well as life-threatening systemic infections, particularly in immunocompromised individuals (7). Besides an altered morphology, hyphae exhibit additional features that contribute to pathogenicity such as the increased production of adhesins, invasins, secreted hydrolytic enzymes, and the toxin candidalysin (8, 9).

*C. albicans* also exhibits the ability to epigenetically switch between “white” and “opaque” states, with the white state considered the default phenotypic state whereas the opaque state is the mating-competent form of the species (10–12). This switch has evolved relatively recently, having been observed only in *C. albicans* and in its close relatives *Candida dubliniensis* and *Candida tropicalis* and regulates sexual competency in each species (13–15). White cells are the more virulent form in a mouse model of systemic candidiasis (16), whereas opaque cells are better at colonization of the skin (17, 18). White and opaque cells also show differences in their interactions with macrophages and neutrophils (19–21), with opaque cells potentially better suited for circumventing immune recognition (22). White-opaque switching occurs stochastically at low frequency and can be induced by a variety of environmental cues including low O_2_, elevated CO_2_, and N-acetyl glucosamine (23–26).

At its core, the white-opaque switch is regulated by a complex network of transcription factors (TFs) that control the formation and inheritance of the opaque state. Wor1 is considered the master regulator of the opaque state and positively regulates its own expression while inhibiting expression of Efg1, which promotes the white state (27–30). In addition to Wor1 and Efg1, six other TFs (Ahr1, Czf1, Ssn6, Wor2, Wor3, and Wor4) operate the core regulatory network that controls switching (27–29, 31–38), while a number of other TFs impact switching frequencies when deleted from the reference isolate SC5314 (34). The white-opaque switch is also regulated by the mating type-like locus (*MTL*) with *MTL***a**/α strains showing lower levels of switching than *MTL* homozygous strains due to repression of the opaque state via the **a**1/α2 complex (11, 39–41).

While multiple TFs regulate white-opaque switching, most of these have been identified using gene deletion assays in a single strain background, that of SC5314 (31, 33–36). In this study, we used a genome-wide approach to identify the set of TFs whose overexpression promotes white-to-opaque switching in WO-1, the strain in which white-opaque switching was first discovered (10). We subsequently tested the set of transcriptional activators of WO-1 switching in SC5314 and found that most TFs induced switching in both strain backgrounds, but that a subset acted in a strain-specific manner. Strikingly, the ability of certain TFs to induce switching was also highly dependent on the media used, with differences in pH, amino acids, and zinc concentrations having major effects on switching frequencies, again in a strain-specific manner. Together, these results identify the set of TFs whose overexpression induces the white-opaque switch and reveal that both strain background and environmental factors can alter TF roles in cell fate determination.

## RESULTS

### Generation and screening of a tetracycline-inducible *C. albicans* TF library

**Fig 1:**
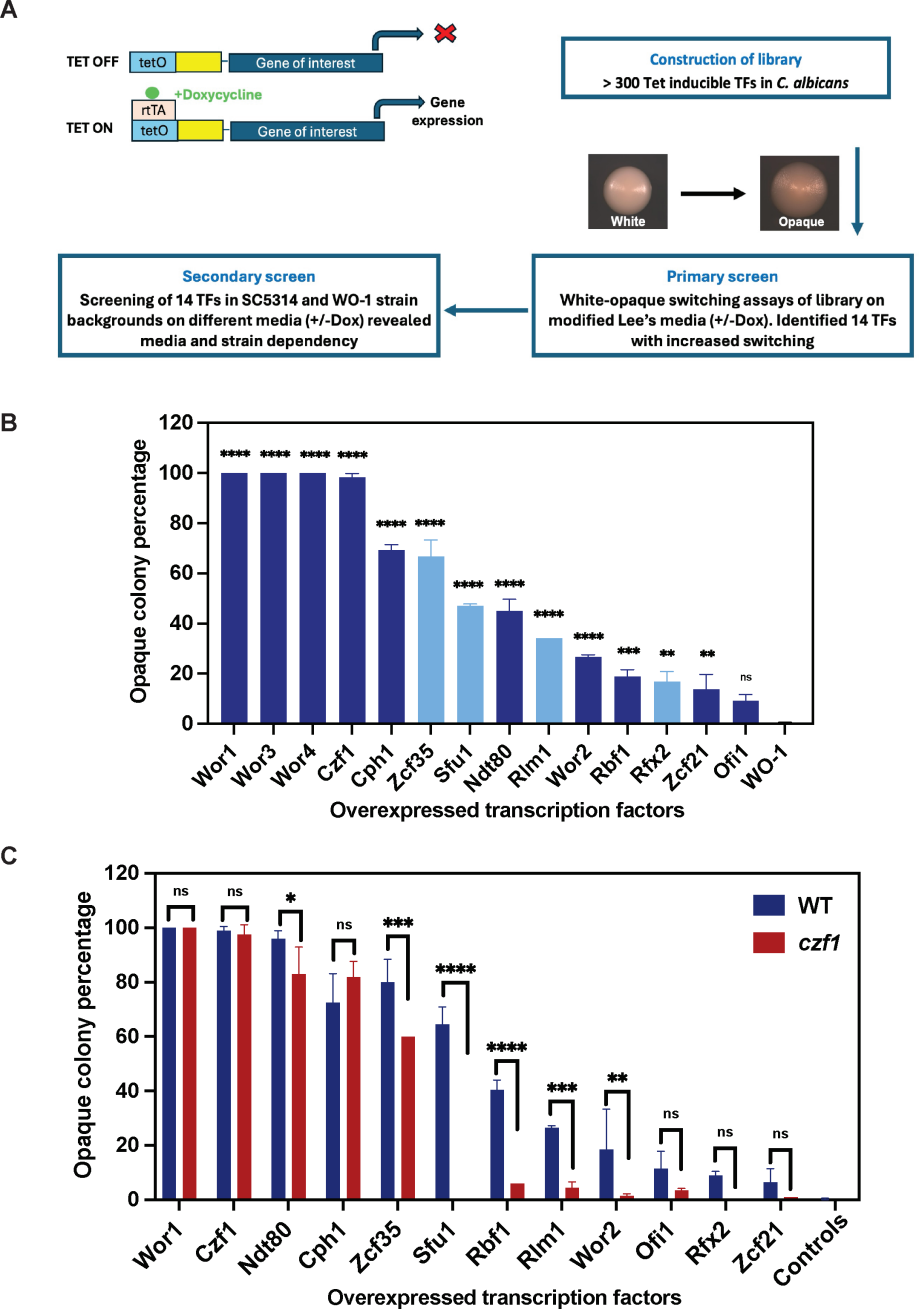
An overexpression screen identifies TFs that induce white-to-opaque switching in *C. albicans* WO-1. (**A**) Schematic of the genome-wide screen for TFs whose overexpression induces white-to-opaque switching. The primary screen identified 14 TFs that significantly increase switching in *C. albicans* strain WO-1 in Lee’s^M^ medium (data in Tables S3 and S8). A secondary screen subsequently examined the role of the 14 TFs in SC5314 and WO-1 under different media conditions. (**B**) The percentage of opaque colonies (including mixed white/opaque) for two independently generated strains is shown in each case. Established regulators of white-opaque switching (*WOR1*, *WOR2, WOR3, WOR4, CZF1, NDT80, CPH1, RBF1, ZCF21,* and *OFI1*) are shown in dark blue, while regulators identified in this study (*SFU1, RFX2, RLM1,* and *ZCF35*) are shown in light blue (Table S3). Significant differences between wild-type WO-1 and TF-expressing strains were calculated using Dunnett’s multicomparison test: ns, *P* > 0.05; **, *P* < 0.01; ***, *P* < 0.001; ****, *P* < 0.0001. (**C**) White-opaque switching frequency of strains expressing the indicated TFs under the control of the Tet promoter in wild-type WO-1 (dark blue bars) or two independently generated *czf1*Δ/Δ derivatives of WO-1 (red bars) (Table S5). Controls include wild-type WO-1 and *czf1*Δ/Δ strains without TFs. Strains were pre-grown in liquid Lee’s^M^ medium in the presence of doxycycline, diluted, and plated on Lee’s^M^ medium without doxycycline to determine the percentage of opaque colonies (including mixed white/opaque colonies). In each case, two independent transformants were tested. Statistical significance was calculated using Tukey’s multicomparison test: ns, *P* > 0.05; *, *P* < 0.05; **, *P* < 0.01; ***, *P* < 0.001; ****, *P* < 0.0001.

Multiple TFs, including the master regulator Wor1, have been identified as regulators of white-opaque switching based on their expression patterns and the phenotypes of the corresponding deletion mutants. To perform a systematic search for TFs that can induce the white-to-opaque switch, we cloned the genes encoding known or putative TFs of *C. albicans* into a tetracycline-inducible expression cassette (see Materials and Methods and Fig. 1A). The resulting library of >300 Tet-inducible TFs (Tables S1 and S2) was integrated into the genome of WO-1, an *MTL*α/α strain in which white-opaque switching was originally discovered, and which has been widely used to study this developmental program. This library has been provided to the Fungal Genetics Stock Center (https://www.fgsc.net/).

To identify TFs whose expression induces switching to the opaque phase, white cells of the parental strain and strains containing the inducible TF library were grown overnight in liquid medium containing doxycycline and then spread at an appropriate dilution on modified Lee’s (Lee’s^M^) medium plates with doxycycline to allow the formation of colonies from individual cells. As a control, cells were cultivated identically except for the absence of doxycycline. The results of this screen are summarized in Table S3. All strains behaved like the parental strain WO-1 when grown in the absence of doxycycline with only basal levels of spontaneous switching to opaque (<3% opaque or mixed white/opaque colonies). In contrast, doxycycline-induced expression of several TFs resulted in increased switching from the white to the opaque phase as shown in Fig. 1B. Eight TFs were known regulators of white-opaque switching: Wor1, Wor2, Cph1, Czf1, Ndt80, Ofi1, Rbf1, and Zcf21. Although not part of the original screen, we also tested the overexpression of Wor3 and Wor4 in WO-1 and found that they efficiently induce white-to-opaque switching as they do in SC5314 (35, 36). We identified four TFs, however, that represent novel regulators of the white-opaque switch: Rfx2, Rlm1, Sfu1, and Zcf35 (light blue in Fig. 1B). The two independently generated strains expressing a specific TF generally behaved similarly, giving confidence in these results (Table S3).

To confirm the results of the primary screen, selected strains that displayed high white-to-opaque switching were retested. In these experiments, cells grown in the presence of doxycycline in the preculture were spread on plates with or without doxycycline to reveal whether transient TF expression during the growth of white cells in liquid culture was sufficient to induce the switch to opaque. In addition, cells grown without doxycycline in the preculture were plated on solid media with and without doxycycline to observe their phenotype when TF expression was induced only during colony development. The results of these experiments are summarized in Table S4 and confirm that ectopic expression of the TFs identified in the primary screen induces white-to-opaque switching. In addition, *OFI1* was found to stimulate switching more strongly in the second round of testing and has been reported in other studies to be a positive regulator of the white-to-opaque switch (42, 43). In contrast, strains overexpressing *RLM1* did not switch to the opaque phase in the second set of experiments. Additional tests showed that Rlm1-induced switching was variable, although two independently constructed strains behaved similarly in a given experiment, suggesting that the ability of this TF to promote switching was influenced by fluctuations in experimental conditions, as expanded on below.

### Dependency of white-opaque switching on the regulators Wor1, Wor2, and Czf1

To investigate if TF induction of white-opaque switching depended on the core regulators Wor1, Wor2, and Czf1, we overexpressed target TFs in *wor1*Δ, *wor2*Δ, and *czf1*Δ mutants of WO-1, respectively. To aid in phenotypic analysis, we generated derivatives of these mutants which expressed *GFP* or *RFP* from the opaque phase-specific *OP4* promoter to determine whether opaque-like colonies contained *bona fide* opaque cells. None of the TFs could promote switching to the opaque state when expressed in white cells in the absence of *WOR1* or *WOR2*, while *CZF1* was dispensable for the induction of switching by a subset of TFs. Like the master regulator Wor1, expression of Cph1, Ndt80, and Zcf35 stimulated white-opaque switching in *czf1*Δ mutants with similar efficiencies to that in the wild-type strain (Fig. 1C; Table S5). In contrast, Sfu1 could not induce switching in the absence of Czf1. For the other tested TFs (Ofi1, Rbf1, Rfx2, Rlm1, Wor2, and Zcf21), which caused a moderate increase in switching in wild-type WO-1, the switching frequency was also lower in the *czf1*Δ background than in the parental strain (Fig. 1C). These results establish that several TFs induce switching in a Czf1-dependent manner, whereas others promote the opaque state independently of Czf1, but all of them require the master regulators Wor1 and Wor2 to induce the white-to-opaque switch.

### Analysis of the impact of TF gene deletions on white-opaque switching

**Fig 2:**
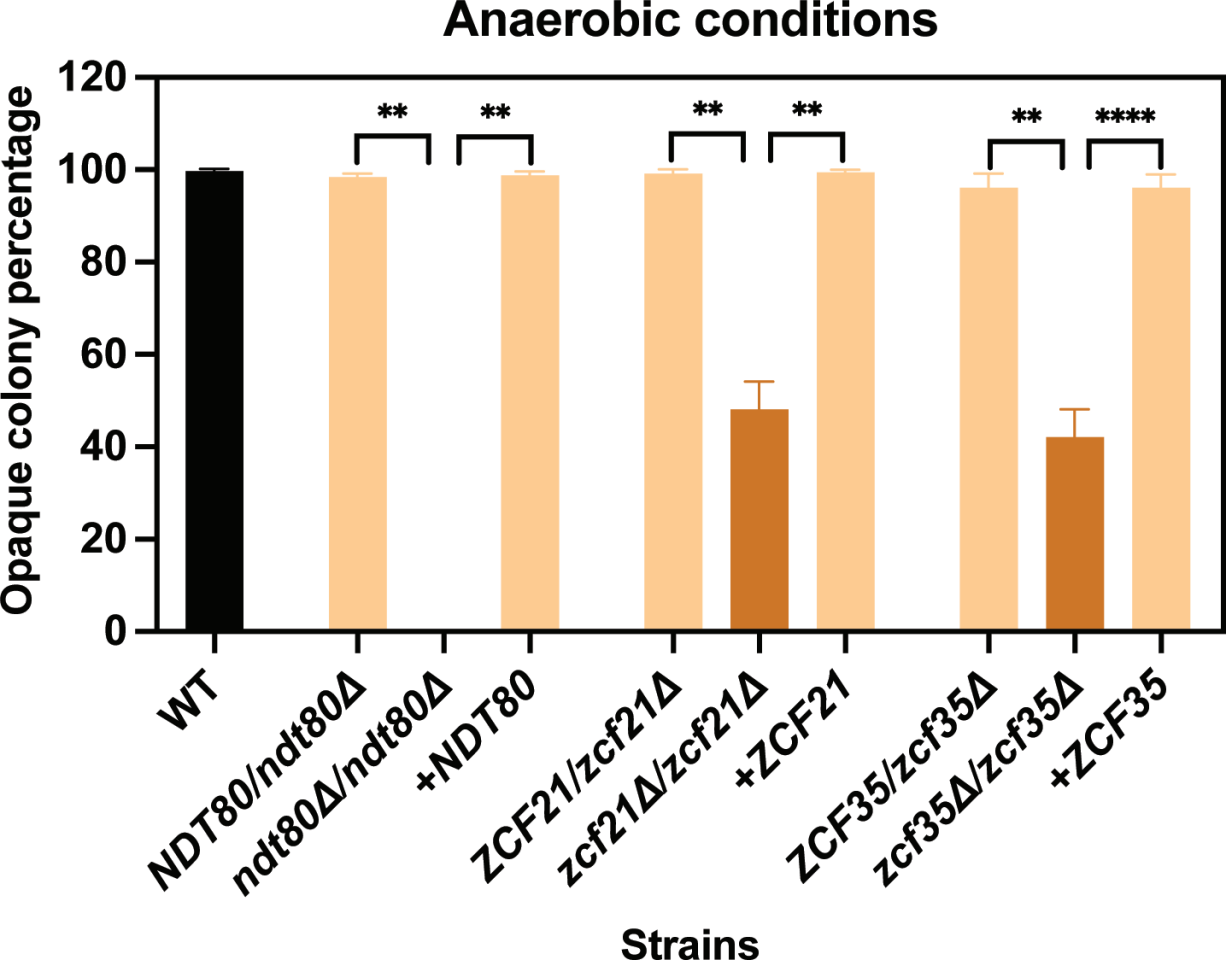
Impact of ectopic TF expression in an anaerobic chamber. White cells from a YPD preculture were diluted 10^−5^, plated on Lee’s^M^ medium, and incubated for 2 days at 22°C in an anaerobic jar, followed by incubation under aerobic conditions at 22°C for an additional 7 days to allow colony development. Heterozygous and homozygous *ndt80*∆, *zcf21*∆, and *zcf35*∆ mutants, along with their corresponding complemented strains and wild-type WO-1, were compared for switching frequencies (data in Table S7). Two independently generated mutants were analyzed for each strain, and the percentage of opaque colonies (including mixed white/opaque colonies) was determined. The means and standard deviations from three biological replicates are shown for each strain. Significant differences between the pair of means were calculated using a Mann-Whitney *U*-test: **, *P* < 0.01.

Several of the TFs identified in our screen, including Wor1, Wor2, Cph1, and Czf1, were previously deleted in WO-1, and the corresponding mutants shown to exhibit reduced white-to-opaque switching (26, 29, 44). Here, we successfully generated *ndt80*Δ, *rfx2*Δ, *rlm1*Δ, *sfu1*Δ, *zcf21*Δ, *zcf35*Δ, and *ofi1*Δ mutants in WO-1, whereas a homozygous mutant was not obtained for *RBF1* (Table S6). Incubation of white cells under certain environmental conditions such as in an anaerobic chamber induces WO-1 cells to switch to the opaque state (26). Strikingly, induction of opaque formation in such a chamber was abolished in *ndt80*Δ mutants, while *zcf21*Δ and *zcf35*Δ mutants exhibited a reduced switching frequency. Switching was restored to wild-type levels upon reintroduction of a functional copy of the respective wild-type gene into *ndt80*Δ, *zcf21*Δ, and *zcf35*Δ mutants (Fig. 2; Table S7). All other mutants tested (*rfx2*Δ, *rlm1*Δ, *sfu1*Δ, and *ofi1*Δ) behaved like the wild-type strain and efficiently switched to the opaque phase after incubation in an anaerobic chamber.

These experiments reveal that switching under these conditions is strictly dependent on *NDT80* as it is on *WOR1* (44). Switching is also partially dependent on Zcf21 and Zcf35, while the other TFs are dispensable for switching in WO-1 as tested here, indicating they may have a redundant function or could be necessary for switching to opaque in response to other environmental signals.

### Transcription factor overexpression induces switching in a media-dependent fashion

The ability of the set of 14 opaque-inducing TFs to induce switching in WO-1 was compared between Lee’s and synthetic complete dextrose (SCD) medium, as well as with a hybrid medium that consisted of an equal mix of Lee’s and SCD components. Interestingly, ectopic expression of TFs induced white-to-opaque switching in a media-specific manner (Table S8), and we therefore classified TFs as “SCD-specific,” “Lee’s-specific,” or “general,” with the latter describing TFs that induce switching independent of the media used. Control assays showed that no induction of white-opaque switching was observed in the absence of doxycycline (Fig. S1), and colonies formed on each media in the presence of doxycycline are shown in Fig. S2. We note that we used a basic Lee’s (Lee’s^B^) medium for these experiments that differs from the modified Lee’s medium used in the earlier experiments (primarily the absence of zinc supplementation, see Materials and Methods and Fig. S3).

**Fig 3:**
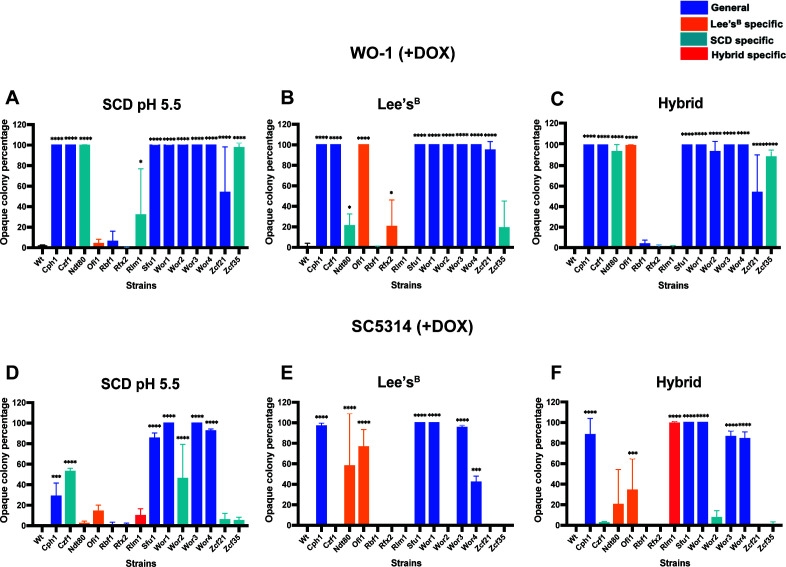
Effect of media and strain background on white-opaque switching. White-opaque switching frequencies were compared on SCD, Lee’s^B^, and hybrid media for WO-1 (**A–C**) and SC5314 (**D–F**) strains expressing different TFs under the doxycycline-inducible promoter (data in Table S8). The percentage of opaque colonies (including mixed white/opaque colonies) is shown for each gene. TFs that induced switching on SCD medium are highlighted in light blue, those that induced switching on Lee’s^B^ medium are shown in orange, and those that induced switching on hybrid medium are shown in red. TFs that induced switching on both SCD and Lee’s^B^ media are represented in dark blue. Data represent switching frequencies measured in the presence of doxycycline for two independent transformants. Asterisks indicate significance compared to the respective wild-type WO-1 or SC5314 controls. Significant differences between the wild-type and each TF-expressing strain were calculated using Dunnett’s multicomparison test: ***, *P* < 0.001; ****, *P* < 0.0001.

The “general” category of opaque-inducing TFs included Cph1, Czf1, Sfu1, Wor1-Wor4, and Zcf21, which induced switching at frequencies ranging from 50% to 100% (Fig. 3A and B). Notably, however, strains overexpressing Ndt80 or Zcf35 resulted in >90% switching on SCD but <30% switching on Lee’s^B^ (Fig. 3A and B). The opposite media dependence was observed when ectopically expressing Ofi1, as this factor induced 100% switching on Lee’s^B^ but less than 5% switching on SCD (Fig. 3A and B). Overexpression of Rlm1 and Rfx2 also showed variable switching; Rlm1 only induced switching on SCD (32% switching), whereas Rfx2 induced switching only on Lee’s^B^ (20% switching), although the increase in switching was not significant compared to the control. Note that experiments were performed concurrently establishing that media-specific differences were responsible for the different phenotypes. Some of these switching frequencies differed from those shown earlier, with these differences likely due to the use of Lee’s^B^ versus Lee’s^M^ media, a point returned to in a subsequent section.

Analysis of Ndt80 and Zcf35 indicated that these TFs induced high switching frequencies on hybrid medium as they did on SCD but not on Lee’s^B^ media (Fig. 3C). Ofi1 also induced high switching on hybrid medium (~100%), similar to overexpression on Lee’s^B^ medium and contrasting with the low switching on SCD (Fig. 3C). On the other hand, Rbf1, Rlm1, and Rfx2 showed no significant induction of switching on the hybrid medium.

Together, these experiments reveal a complex interrelationship between TF overexpression, media dependency, and white-opaque switching. While multiple TFs induced efficient switching to opaque independent of media composition, Ndt80, Ofi1, and Zcf35 showed a strong media dependency for induction of switching. Media mixing experiments implicate specific components in SCD and Lee’s media as impacting switching, as investigated below.

### Transcription factors induce white-opaque switching in a strain-dependent manner

To explore whether strain background impacts TF-induced switching, we overexpressed the set of 14 TFs that induce switching in WO-1 in an *MTL***a**/**a** derivative of SC5314, the standard reference strain of *C. albicans*. Several TFs induced high switching frequencies in SC5314 as in WO-1, including Sfu1, Wor1, Wor3, and Wor4, which typically induced switching in 80%–100% of colonies (Fig. 3D through F). In contrast, Zcf21 and Zcf35 induced efficient switching in WO-1 on one or more media conditions but did not induce switching in SC5314 on any of the three media tested (Fig. 3D through F). TFs again showed a medium-specific capacity to induce switching when overexpressed in SC5314 (Table S8). For example, Czf1 or Wor2 induced high levels of switching in WO-1 on all three media, yet these factors efficiently induced switching in SC5314 only on SCD medium (~50% switching), with little activity observed on Lee’s^B^ or hybrid media (Fig. 3D through F). Ndt80 and Ofi1 also showed media-dependent switching in SC5314 as these TFs only induced high (>90%) switching on Lee’s^B^ medium (Fig. 3D through F). Notably, Ndt80 showed opposite media-specific dependencies between SC5314 and WO-1; this factor induced 100% switching in WO-1 on SCD medium but only ~20% switching on Lee’s^B^, whereas this trend was reversed in SC5314 with 2% switching on SCD and 67% switching on Lee’s^B^ (Fig. 3A thorugh E). A similar result was obtained with SC5314 overexpressing Rlm1, as this TF only induced switching on hybrid SCD/Lee’s^B^ medium (Fig. 3D through F) and did not induce switching in WO-1 (Fig. 3C). Rlm1 therefore represents an example of both a media- and strain-dependent switching factor. Overall, these experiments establish that both strain background and media-dependent cues impact the frequency of TF-induced white-to-opaque switching.

### Media components that impact TF-induced switching

**Fig 4:**
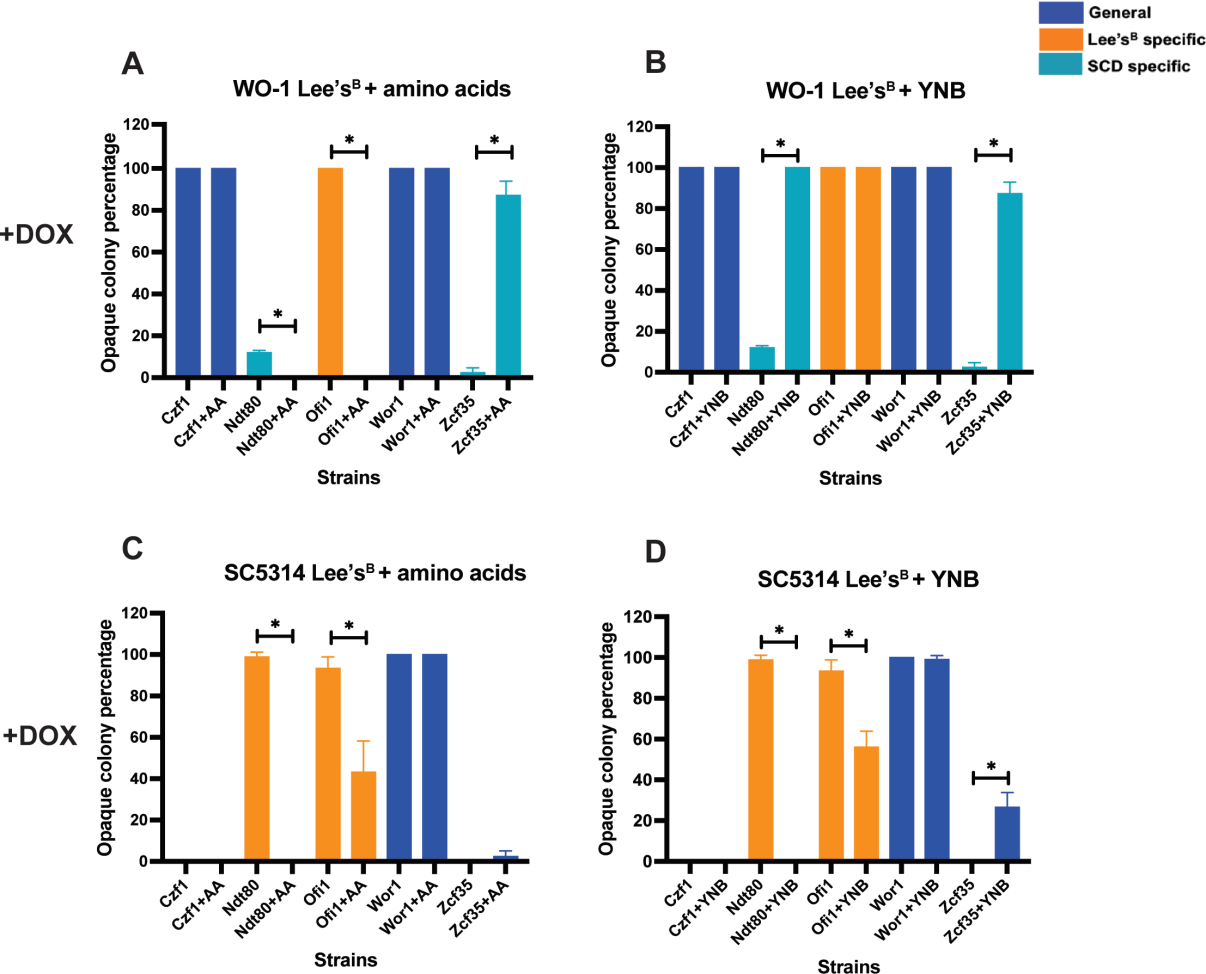
Impact of amino acids and YNB on TF-induced white-opaque switching. Switching assays were performed on Lee’s^B^ medium with or without supplementation of amino acids and YNB for WO-1 (**A, B**) and SC5314 (**C, D**) strains ectopically expressing Czf1, Ndt80, Ofi1, Wor1, or Zcf35 (data in Tables S9A and B). TFs that induced switching on SCD medium or Lee’s^B^ medium are highlighted in light blue or orange, respectively. TFs that induced switching on both media are shown in dark blue. Significant differences between the pair of means were calculated using the Mann-Whitney *U*-test: *, *P* < 0.05.

We further examined the media-specific factors influencing the ability of Czf1, Ndt80, Ofi1, and Zcf35 to induce switching. SCD and Lee’s^B^ media differ in composition with Lee’s^B^ containing lower levels of amino acids and yeast nitrogen base (YNB) than SCD (Fig. S3), and we therefore tested the impact of these components on switching (see Tables S9A and B). In the WO-1 background, supplementation of Lee’s^B^ medium with amino acids suppressed switching by Ndt80 and Ofi1 (100% to 0% in the latter), yet increased switching by Zcf35 (from 2% to 100%) (Fig. 4A; Table S9A). For Ofi1 and Zcf35, these results are consistent with the switching differences seen between SCD (high amino acids) and Lee’s^B^ medium (low amino acids). However, they are contrary to the phenotype expected with Ndt80, which shows high switching on SCD but not on Lee’s^B^ medium, so that higher amino acids would be expected to increase switching if this component was driving the phenotype. The addition of amino acids to Lee’s^B^ medium did not alter switching by Czf1 or Wor1 in WO-1.

Supplementation of Lee’s^B^ medium with YNB significantly increased WO-1 switching frequencies mediated by “SCD-specific” Ndt80 and Zcf35 (100% switching for both in the presence of YNB) (Fig. 4B; Table S9A), but did not change switching induced by Ofi1, Czf1, or Wor1. The enhancement of switching by YNB for Ndt80/Zcf35 is in line with the differences observed between Lee’s^B^ medium (low switching/low YNB) and SCD medium (high switching/high YNB) for these TFs expressed in WO-1.

Switching frequencies were similarly analyzed for TF overexpression in SC5314. Here, we observed that the addition of amino acids or YNB to Lee’s^B^ medium led to decreased switching by the “Lee’s-specific” factors Ndt80 and Ofi1 (Fig. 4C and D; Table S9B), consistent with the suppression of switching on SCD being due to the higher levels of YNB present relative to Lee’s^B^ medium. Unexpectedly, however, Zcf35 demonstrated a significant increase in switching upon addition of YNB to Lee’s^B^ medium (Fig. 4D), even though this TF induced low switching on both SCD and (non-supplemented) Lee’s^B^ media. No switching was induced by Czf1 on Lee’s^B^ medium under either condition, indicating induction of switching by Czf1 on SCD is not due to the higher levels of YNB or amino acids (at least when these components are added individually). Wor1 induced high switching frequencies in SC5314 independent of the conditions used.

These results show that the addition of amino acids or YNB can substantially alter TF-induced switching in both WO-1 and SC5314. Moreover, these components can enhance or suppress switching depending on the TF, the media, and the strain background. This is exemplified by Ndt80 for which the addition of YNB to Lee’s^B^ medium enhanced Ndt80-induced switching in WO-1 but suppressed it in SC5314.

### Effect of pH on TF-induced switching

**Fig 5:**
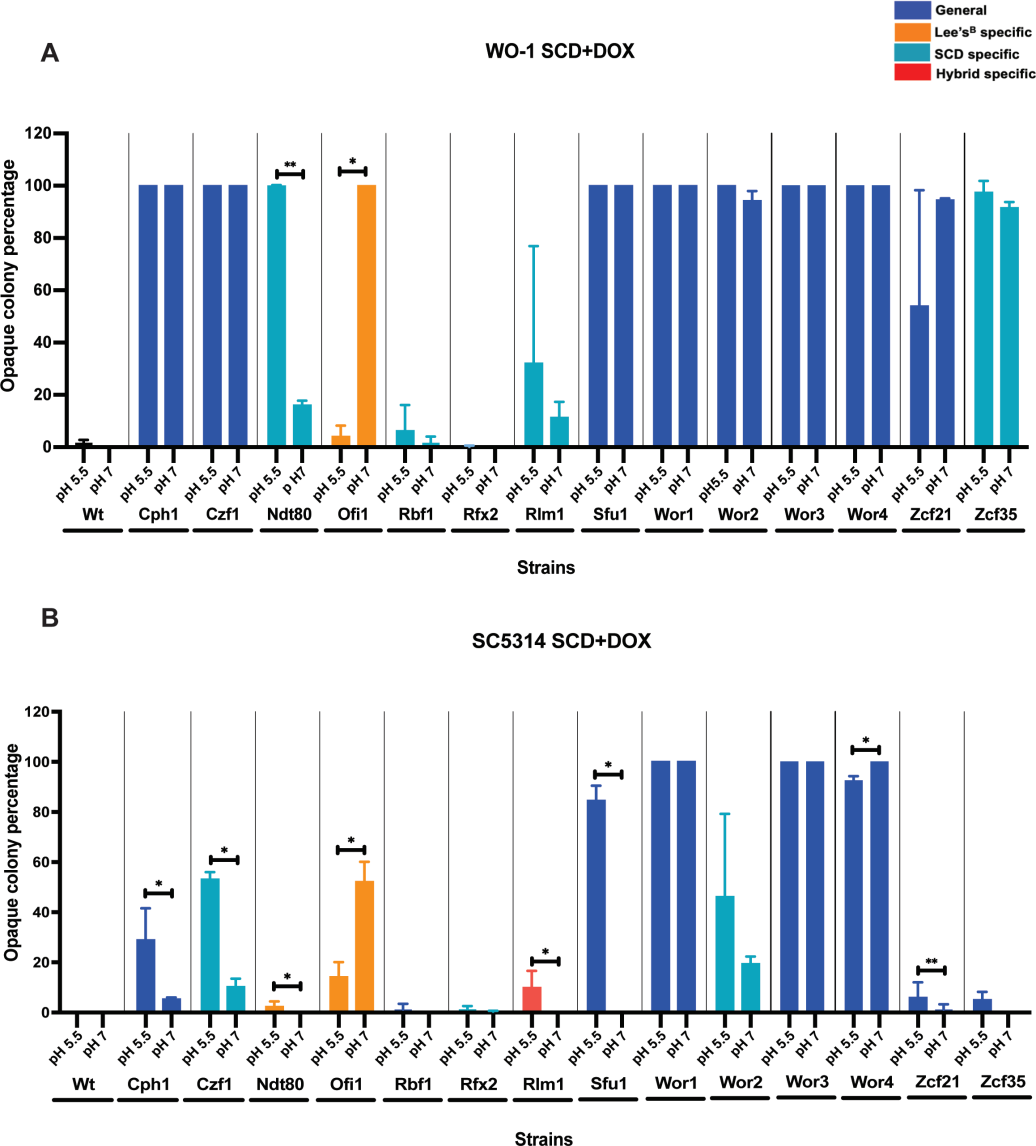
Impact of pH on TF-induced white-opaque switching. Switching assays were performed on SCD medium at pH 5.5 and pH 7 for different inducible TFs expressed in (**A**) WO-1 or (**B**) SC5314 (data in Table S8). The percentage of opaque colonies (including mixed white/opaque colonies) is shown for each gene. TFs that induced switching on SCD are highlighted in light blue, while those inducing switching on Lee’s^B^ or hybrid media are denoted by orange and red, respectively. TFs that induced switching on both SCD and Lee’s^B^ media are shown in dark blue. Significant differences between pairs of means were calculated using a Mann-Whitney *U*-test: *, *P* < 0.05; **, *P* < 0.01.

Changes in pH are known to alter white-to-opaque switching*,* with an acidic pH linked to increased switching (45). Given differences in the pH of SCD (pH 5.5), Lee’s (pH 7.2), and hybrid (pH 6.4) media, we investigated the effect of pH on TF-induced switching by comparing SCD at pH 5.5 to that adjusted to pH 7 (Table S8). In WO-1, a significant decrease in switching was observed at pH 7 compared to pH 5.5 when expressing the “SCD-specific” TF Ndt80 (16% versus 100%). Conversely, expression of Ofi1, a “Lee’s-specific” TF, exhibited a marked increase in switching at pH 7 (100% switching) compared to pH 5.5 (3% switching) (Fig. 5A). These results are in line with what would be expected based on the results of switching on SCD that has an acidic pH (where Ndt80 induces high switching and Ofi1 low switching) versus Lee’s medium that has a neutral pH (where these effects are reversed). In contrast to Ndt80 and Ofi1, we did not observe a significant effect of pH on switching induced by any other TF expressed in WO-1 (Fig. 5A).

In the SC5314 background, expression of Cph1, Czf1, Ndt80, Rlm1, Sfu1, and Zcf35 showed significantly lower switching on SCD at pH 7 than at pH 5.5 (Fig. 5B). Interestingly, similar to observations in WO-1, the “Lee’s-specific” TF Ofi1 showed increased switching in SC5314 at pH 7 compared to pH 5.5 (Fig. 5B). Wor4 also induced more switching at pH 7 than at pH 5.5 (Fig. 5B; Fig. S2). Ofi1 and Wor4 therefore induce more switching at a neutral pH than at an acidic pH, unlike the other TFs tested. These data further highlight the interdependency of TF-induced switching with the media conditions used, including an effect of pH.

### Zinc-dependent regulation of TF-induced switching

Our initial TF overexpression screen was performed using modified Lee’s medium (10, 46–48) that includes zinc supplementation, whereas subsequent experiments used Lee’s^B^ that lacks zinc supplementation. In parallel, we fortuitously noted that Ndt80 overexpression produced distinct results when using Lee’s^B^ prepared with two different water sources. Analysis revealed that one water source (WS1) contained 0.52 µM zinc, whereas the second (WS2) contained 1.65 µM zinc. A commercial water source (Gibco) was also tested that had zinc levels below detectable limits. In comparison, SCD medium is contains 2.5 µM zinc sulfate as standard.

**Fig 6:**
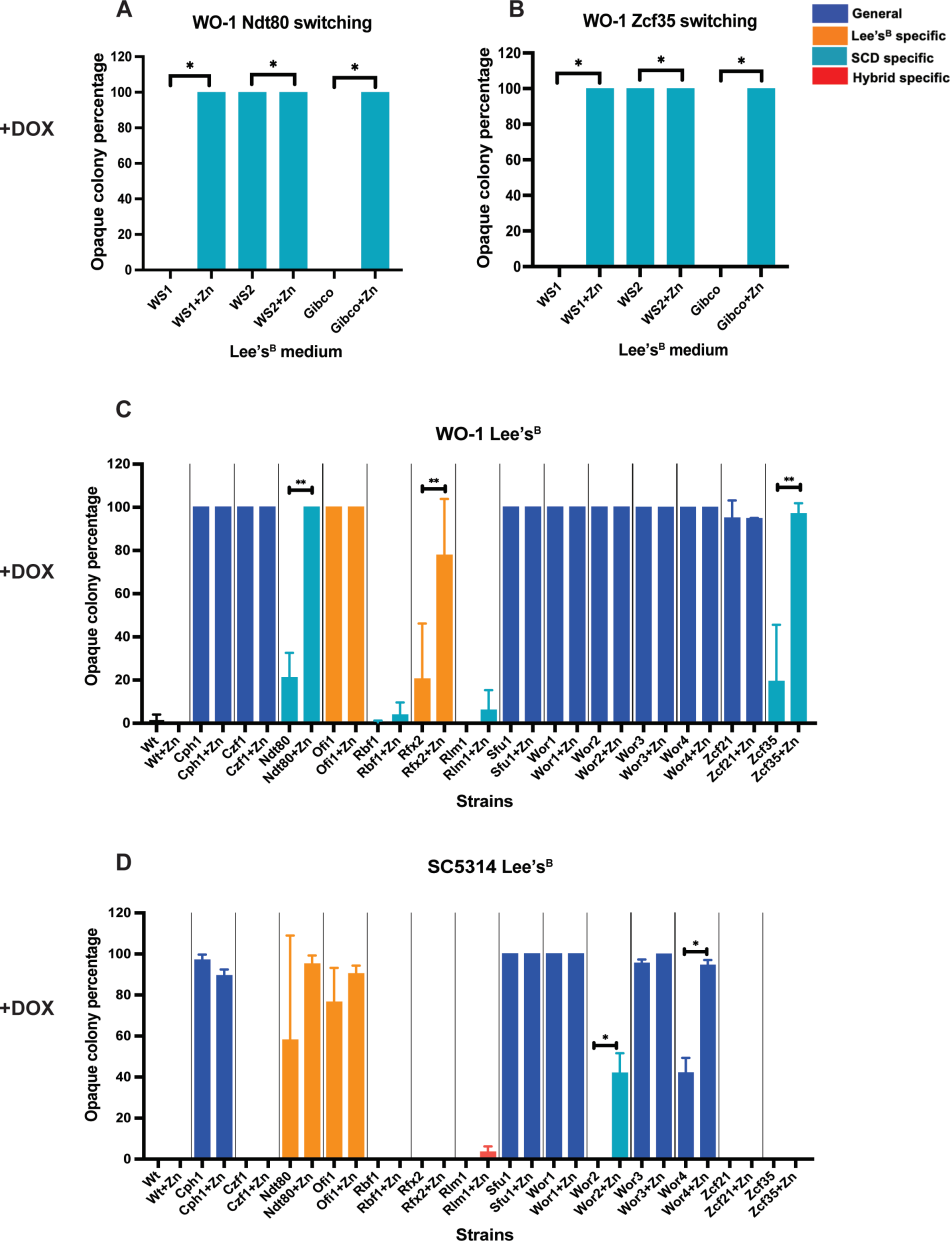
Impact of water sources and zinc concentrations on TF-induced white-opaque switching. Switching assays were performed with Ndt80 (**A**) or Zcf35 (**B**) expressed in WO-1 on Lee’s^B^ medium prepared with three different water sources (WS1, WS2, or Gibco) (data in Table S9C). Assays were also performed with or without additional zinc supplementation (1.4 µM) for WO-1 (**C**) and SC5314 (**D**) strains expressing target TFs (data in Table S8). The percentage of colonies with opaque (or mixed white/opaque colonies) phenotypes is provided for each gene. TFs that induce switching only on SCD medium are highlighted in light blue, while those that induce switching only on Lee’s^B^ or hybrid media are denoted by orange and red, respectively. TFs that induce switching on both SCD and Lee’s^B^ media are shown in dark blue. Significance between pairs of means was calculated using a Mann-Whitney *U*-test: *, *P* < 0.05; **, *P* < 0.01.

To determine if zinc concentrations impact TF-induced switching, we repeated Ndt80 and Zcf35 assays using Lee’s^B^ medium made with WS1, WS2, or Gibco water with/without supplementation with 1.4 µM zinc sulfate. Notably, Ndt80 and Zcf35 did not induce switching in WO-1 using Lee’s^B^ medium made with Gibco or WS1 water but induced 100% switching in medium prepared with WS2 water. Furthermore, zinc supplementation resulted in Ndt80 and Zcf35 inducing 100% switching in media made with all three water sources (Fig. 6A and B; Table S9C), establishing that zinc promotes the induction of switching by these TFs.

We also examined the impact of zinc supplementation on white-opaque switching for the remaining set of TFs by comparing Lee’s^B^ medium (made with WS1 water) ± zinc supplementation. Ndt80 and Zcf35 were repeated and showed the same strong enhancement of switching with zinc supplementation (Fig. 6C and D). Rfx2 expression in WO-1 and Wor2/Wor4 expression in SC5314 also induced higher levels of switching in the presence of extra zinc. Rfx2 induced 78% switching in WO-1 on Lee’s^B+Zn^ compared to only 20% switching on Lee’s^B^ lacking added zinc (Fig. 6C and D), while Wor2/Wor4 induced 41%/94% switching in SC5314 in medium containing zinc versus 0%/42% switching without supplementation (Fig. 6D). These experiments establish that the capacity of a subset of TFs to induce white-opaque switching is strongly dependent on the presence of zinc both in WO-1 and SC5314.

### Impact of the expression of target TFs on cellular growth

Previously, strains with slower growth rates were reported to exhibit an increased frequency of white-to-opaque switching (23). We therefore examined the growth rates of TF overexpression strains in SCD and Lee’s^B+Zn^ media containing 0, 25, or 50 µg/mL doxycycline. In SCD, strains generally reached an OD_600_ of ~1.4 within 48 h, while growth was slower in Lee’s^B+Zn^ medium reaching an OD_600_ of ~1.0 in 72 h (Fig. S4A; Tables S10 and S11). In the SC5314 background, six TF-expressing strains showed substantially reduced growth rates (Cph1, Czf1, Ndt80, Ofi1, Rbf1, and Rfx2) in both SCD and Lee’s^B+Zn^ media, with the growth reduction in Lee’s^B+Zn^ medium observed even in the absence of doxycycline (Fig. S4A). In WO-1, induction of a subset of TFs also reduced growth rates; three TFs (Cph1, Ofi1, and Rfx2) slowed growth by more than 20% in SCD medium and nine TFs (Cph1, Ndt80, Ofi1, Rbf1, Rfx2, Wor1, Wor2, Wor3, and Wor4) did so in Lee’s^B+Zn^ medium (Fig. S4A). For both SC5314 and WO-1, growth rates for TFs were generally slower at the higher doxycycline concentration than at the lower concentration (Fig. S4A).

Next, we compared the growth rates and switching frequencies of TF overexpressing strains in the two media conditions. In total, the set of 14 TFs were evaluated in 56 different strain/media combinations (SC5314 vs WO-1 and SCD vs Lee’s^B+Zn^). Of the 56 combinations, 27 led to significantly slower growth rates than the no-doxycycline control, and 18 of these 27 showed elevated switching frequencies (Fig. S4B; Table S11). Conversely, 21 out of the 56 combinations resulted in elevated switching frequencies without a significant change in the growth rate. We also examined the correlations between growth rates and switching frequencies across experiments. No clear correlation was observed between slower growth rates and switching frequencies in SC5314 or WO-1 upon ectopic TF expression (Fig. S4B). Overall, it is evident that slower growth rates do not consistently result in increased switching, and a similar conclusion was reached from a large-scale analysis of TF deletion mutants (34).

### Impact of strain and media conditions on TF expression levels

Given that media and strain background differences can impact TF-induced switching, we directly examined the expression levels of two TFs, Ndt80 and Ofi1, using strains expressing TF-mNeonGreen fusion proteins. We note that mNeonGreen-tagged strains showed similar switching frequencies to those of untagged strains (Fig. S5; Table S12A), and that doxycycline-induced expression of Ndt80 and Ofi1 was observed under all test conditions in both WO-1 and SC5314 (Fig. S5). For Ndt80, expression varied by ~3-fold between experiments and was highest in SC5314 cells grown in Lee’s^B^ medium (Fig. S5A and B; Table S12B), even though switching frequencies were highest in WO-1 cells (with 100% switching observed in SCD pH 5.5 and Lee’s^B+Zn^) (Fig. S5A and B; Table S12A). For Ofi1, expression levels also varied by ~3-fold between conditions, with expression in SCD pH 7 medium two- to threefold higher than that in SCD pH 5.5 medium for both WO-1 and SC5314 (Fig. S5C and D; Table S12B). This result is in line with differences in switching, as both strain backgrounds showed higher switching with Ofi1 overexpression at pH 7 than at pH 5.5 (Fig. S5C and D; Table S12A). Taken together, these results suggest that certain differences in switching frequencies could be due, in part, to differences in TF overexpression levels, although this cannot account for all the differences observed, including those for Ndt80-mediated switching where expression levels did not correlate with switching frequencies.

### Impact of ectopic TF expression on core TF regulators of white-opaque switching

The white-opaque switch is regulated by a core network of eight TFs: Ahr1, Czf1, Efg1, Ssn6, Wor1, Wor2, Wor3, and Wor4. To test if ectopic expression of other opaque-inducing TFs alters the levels of core network TFs, a Nanostring analysis of gene expression was performed. WO-1 strains expressing Ndt80, Rfx2, and Zcf35 and the parental isolate were grown in Lee’s^B+Zn^ medium ± doxycycline for 12 h. We found that ectopic expression of these TFs generally did not alter expression of any of the core network TFs, although Zcf35 resulted in a modest 1.6-fold increase in *WOR1* expression (Fig. S6; Table S13). These findings indicate that ectopic expression of three target TFs has little direct effect on the expression of the core white-opaque network TFs and therefore influence switching independently of the core TFs.

## DISCUSSION

Transcriptional regulation of the *C. albicans* white-opaque switch has been extensively studied since its discovery in strain WO-1 in 1987 (10). Eight TFs are now known to form the core opaque network (33, 49), with these TFs binding both to their own promoters and to each other’s promoters forming multiple positive feedback loops (31–33). Wor1 is considered the most important opaque regulator, and its expression levels are tightly linked to the formation and stability of the opaque state (27, 30, 50). In addition to the core TF network, a set of 108 “auxiliary” TFs impact white-opaque switching as identified by screening of gene deletions in SC5314 (51).

### Transcriptional regulators of the white-opaque switch

In this work, we further examined the regulation of white-opaque switching by constructing and screening a TF overexpression library in the WO-1 strain background. We show that 14 TFs can induce white-to-opaque switching, including 10 TFs that were previously identified as positive regulators of the switch: Wor1-Wor4, Cph1, Czf1, Ndt80, Ofi1, Rbf1, and Zcf21. Four novel white-opaque regulators were found: Rfx2, Rlm1, Sfu1, and Zcf35. Interestingly, those TFs with the strongest ability to induce switching did so independently of Czf1, whereas those with weaker abilities induced switching in a Czf1-dependent manner. This suggests that the latter TFs must cooperate with Czf1 to induce the white-to-opaque switch. We also found that induction of three opaque-inducing TFs (Ndt80, Rfx2, and Zcf35) did not have a substantial effect on expression of core white-opaque network TFs, indicating that they do not induce switching by directly altering the expression of core TFs.

In all cases, induction of white-to-opaque switching required Wor1 and Wor2, consistent with these TFs being central to opaque cell formation. This is in line with chromatin immunoprecipitation data showing that Wor1 and Wor2 play prominent roles in recruiting other TFs to the promoters of genes in opaque cells (32, 33). Wor1 is required both for switching to opaque and for maintenance of this state, whereas Wor2 is thought to be essential for propagation of the opaque state but dispensable for the white-to-opaque transition. This model is based on studies where *WOR2* overexpression did not increase white-to-opaque switching, but deletion of the gene destabilized the opaque state (31). We similarly observed that *WOR2* overexpression generally failed to induce white-to-opaque switching in SC5314, and yet it effectively induced the opaque state in WO-1. Such differences could reflect the higher propensity of WO-1 to undergo white-to-opaque switching than SC5314, making WO-1 more sensitive to *WOR2* levels than SC5314. These experiments establish that *WOR2* can promote switching to the opaque state in addition to stabilization of this state once formed.

### Environmental factors and strain background impact white-opaque switching

**Fig 7:**
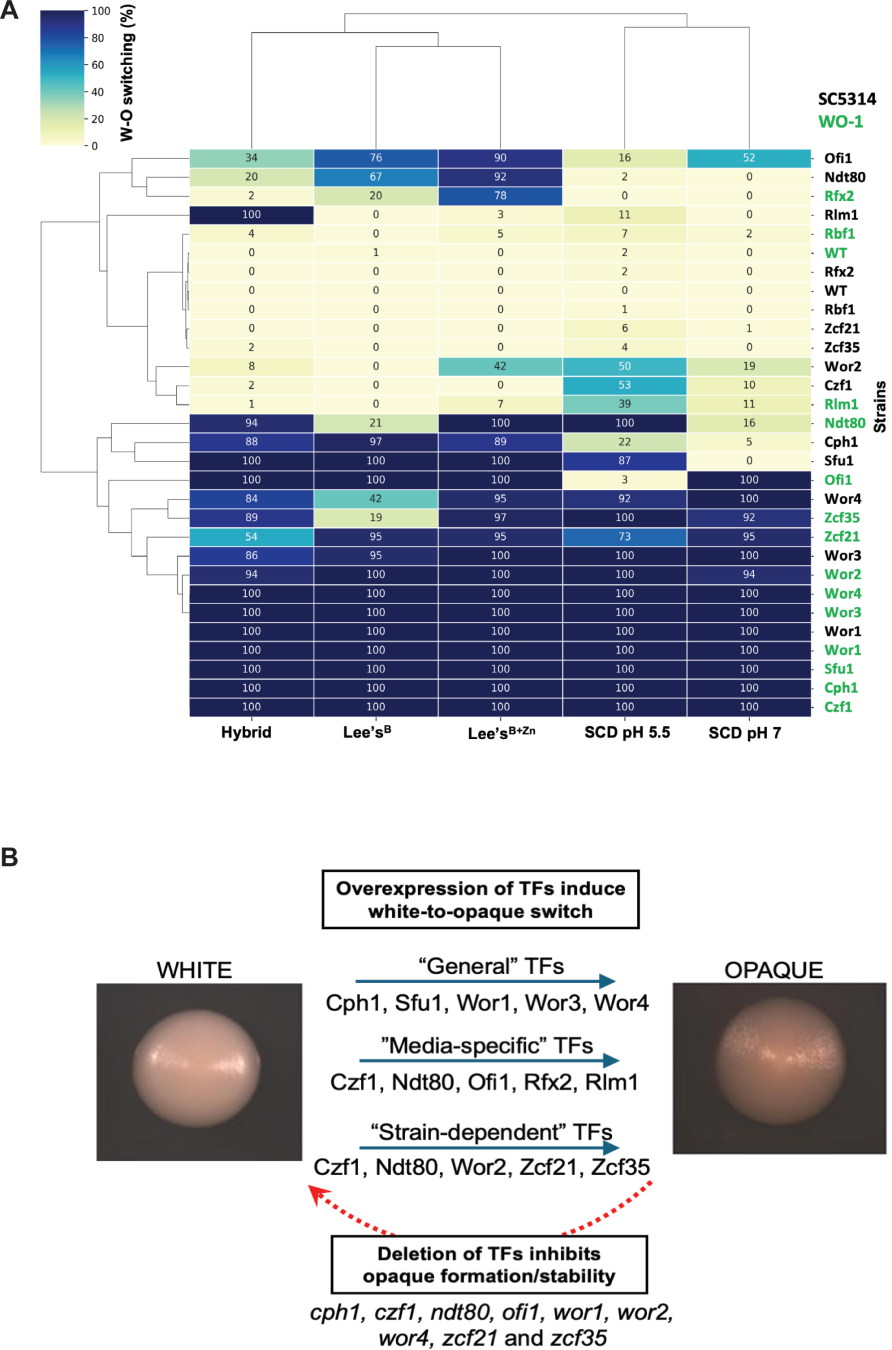
Summary of the effect of TF overexpression on white-opaque switching. (**A**) Heat map from hierarchical clustering of the switching frequencies of doxycycline-induced TFs in SC5314 and WO-1 strains across different media conditions. The color scale represents white-to-opaque switching frequencies ranging from 0 to 100%. (**B**) The set of 14 TFs whose overexpression induces the white-to-opaque switch are grouped into three categories: (i) “general” TFs that induce switching independent of strain background and media, (ii) “media-specific” TFs that induce switching only in certain media conditions, and (iii) “strain-specific” TFs that preferentially induce switching in SC5314 or WO-1 but not in both strains. Solid blue lines indicate switching induced by TF overexpression. The red dotted line represents those TFs (out of the set of 14) whose deletion causes a reduction in opaque formation. Representative images of white and opaque colonies are the same as those used in Fig. 1.

A key finding from the current study was the extreme sensitivity of TF function to genetic and environmental factors. These include differences between strain backgrounds (WO-1 vs SC5314) and between different media, as summarized in Fig. 7. There was also a complex interplay between media- and strain-specific factors, as certain chemical cues supported the function of a given TF in one strain background but not in the other. In particular, pH, zinc, amino acids, and YNB strongly affected white-opaque switching frequencies induced by Ndt80, Ofi1, and Zcf35. For example, Ndt80 and Zcf35 induced no switching in WO-1 on Lee’s^B^ medium, yet both TFs induced 100% switching on the same medium supplemented with zinc. Strain-specific differences were also notable with Ndt80-induced switching enhanced by YNB in WO-1 but suppressed by YNB in SC5314. Similarly, Zcf35 induced 100% switching in WO-1 on Lee’s medium containing amino acids but did not induce switching in SC5314 under the same conditions. Such differences were not due to differences in TF overexpression levels between strains or culture conditions. These results highlight that TF function is exquisitely sensitive to both environmental conditions and strain background, and that a complex interplay exists so that changes in one can alter the impact of the other.

We also evaluated TF function in an anaerobic jar, an oxygen-free environment containing 18% CO_2_ that efficiently induces white-to-opaque switching (26). Deletion of Ndt80 blocked switching in these conditions, as does loss of Wor1 or Czf1 (44), whereas *rfx2*Δ, *rlm1*Δ, *sfu1*Δ, and *ofi1*Δ mutants showed normal switching. Ndt80 activity appears particularly sensitive to assay conditions, suggesting that this factor acts as a sensor of environmental cues, including those involving changes in oxygen/CO_2_ levels. These observations also have implications for the environmental control of drug resistance and biofilm formation as Ndt80 is implicated in regulating both of these processes in *C. albicans* (52, 53).

### Strain background impacts TFs regulating white-opaque switching

Interestingly, increased white-to-opaque switching in an anaerobic jar occurs in WO-1 but not in other *C. albicans* isolates (26), highlighting strain-specific differences in behavior. Other studies have also revealed strain-specific properties related to the white-opaque switch, including a recent study that showed that SC5314 *MTL***a**/α cells are prevented from undergoing switching as they express four TFs at a higher level than switching-competent *MTL***a**/α isolates (51). Increased expression of Ofi1 in certain clinical *MTL***a**/α isolates has also been linked to their ability to undergo white-opaque switching (43). Moreover, some *C. albicans* isolates are naturally *efg1* null and thus exhibit increased switching to opaque (54, 55). Strain-specific differences similarly exist in filamentation and biofilm formation and for which the regulatory TFs overlap with those controlling white-opaque switching (18, 49, 51, 52, 56–59). Interestingly, Wor1 cooperates with Efg1 to drive biofilm formation (60) and yet Wor1 and Efg1 are antagonistic to one another in white-opaque switching, providing a striking example of how even the directionality of TF interactions can differ between different developmental programs. The current study further highlights how strain-specific differences can have striking effects on TF-induced phenotypes.

### Impact of pH and zinc on TF-induced white-opaque switching

pH levels have important consequences for *C. albicans* biology, with acidic pH previously shown to promote white-to-opaque switching via the Rim101 pH sensing pathway (45). In line with this, we observed that several TFs (Cph1, Czf1, Ndt80, and Sfu1) induced higher switching to opaque at an acidic pH than at a neutral pH, consistent with cells being primed to switch at the lower pH. Notably, however, the effect was TF specific, as Ofi1 induced more white-to-opaque switching at neutral pH than at acidic pH, both in WO-1 and in SC5314.

The fact that zinc concentrations can impact *C. albicans* properties has long been recognized, with micromolar amounts shown to promote growth to a higher cell density but to suppress filamentation (46, 61, 62). The importance of zinc has led to the frequent use of modified Lee’s medium in which zinc sulfate is included as a supplement (47, 48). We found that Lee’s supplemented with zinc often produced larger colonies than non-supplemented medium (Fig. S2) as well as enhancing the function of a subset of TFs. We note that YNB also contains zinc, and thus, some of the effects of YNB supplementation could be due to changes in zinc concentrations, although YNB and zinc did not have interchangeable effects. In addition, doxycycline can act as a divalent metal ion chelator (63) and thus could potentially deplete the availability of zinc to *C. albicans* cells. In the future, analysis of TFs using different inducible promoter systems could therefore further help define the role of zinc in TF function. More broadly, it is evident that zinc plays a pivotal role in both filamentation and white-opaque switching in *C. albicans*, and generally exerts opposite effects on these programs. This mirrors previous studies that showed that the factors promoting white-to-opaque switching and filamentation are often antagonistic to one another (51).

### Conclusions

This study expands the repertoire of TFs that regulate white-opaque switching and highlights the sensitivity of TF function to environmental cues and strain background. The extreme sensitivity of the switch to even relatively small changes in assay conditions may reflect TFs having to undergo phase separation to be functional, as this process is highly sensitive to changes in protein concentration and environmental factors (64, 65). Our experiments also provide a striking example of how experimental differences can arise due to “differences in the water” as differences in zinc concentrations between water sources significantly impacted TF function. Finally, we note that the TF overexpression library developed here will provide an important tool for further addressing the function of TFs in *C. albicans*, an important human pathobiont.

## MATERIALS AND METHODS

### Strains and growth conditions

*C. albicans* strains used in this study are listed in Table S6 and were stored as frozen stocks with 15% glycerol at −80°C. Strains were routinely grown in liquid YPD medium (1% yeast extract, 2% peptone and 2% dextrose) at 30°C in a shaking incubator. For the selection of nourseothricin-resistant transformants, 200 µg/mL nourseothricin (Werner Bioagents, Jena, Germany) was added to YPD agar plates. To obtain nourseothricin-sensitive derivatives in which the *SAT1* flipper cassette was excised by FLP-mediated recombination, transformants were grown overnight in YCB-BSA-YE medium (23.4 g yeast carbon base, 4 g bovine serum albumin, 2 g yeast extract per liter, pH 4.0) without selective pressure to induce the *SAP2* promoter controlling *caFLP* expression.

### Construction of the Tet-inducible transcription factor library

To generate a library containing known or putative transcription factors of *C. albicans*, we compiled genes that were annotated with the GO term “transcription factor activity” in the *Candida* Genome Database (CGD; http://www.candidagenome.org) (66). Additional candidate genes with related functions as well as known hyperactive alleles of some transcription factors were also included, producing a list of 343 confirmed or potential transcription factors (Tables S1 and S6). The plate number and well position of each strain are available in Table S3. Most genes were amplified from the genomic DNA of strain SC5314 by PCR with primers that introduced a SalI site in front of the start codon and a BglII site behind the stop codon. For genes with internal SalI or BglII sites, primers containing compatible XhoI and/or BamHI sites were used. The PCR products were appropriately digested and cloned in place of the *GFP* reporter gene in the SalI/BglII-digested vector pNIM6 (26). Several genes were already present in our collection from previous work and cloned in the vector pNIM1, which is identical to pNIM6 but contains the *ACT1* transcription termination sequence instead of the *TEF3* terminator behind *GFP* (67). All cloned genes were completely sequenced and compared with the *C. albicans* genome sequence (Table S2). Silent polymorphisms were accepted even when they were not found in CGD. Polymorphisms that resulted in amino acid substitutions that had not been previously described were confirmed by sequencing of a second clone from an independent PCR reaction.

### Other plasmid constructions

For deletion of *NDT80*, *RBF1*, *RFX2*, *RLM1*, *SFU1*, *ZCF21*, *ZCF35*, and *OFI1* genes, the upstream and downstream regions of these genes were amplified as SacI-SacII and XhoI-ApaI fragments, respectively, and cloned on both sides of the *SAT1* flipper cassette in plasmid pSFS5, a derivative of plasmid pSFS2 (68) in which the *caFLP* gene is placed under the control of the *SAP2* promoter instead of the *MAL2* promoter (69) to result in pNDT80M3, pRBF1M3, pRFX2M3, pRLM1M1, pSFU1M3, pWOR2M3, pZCF21M2, pZCF35M3, and p4972M3, respectively. Deletion of *WOR2* was performed as previously described (44). For complementation of the *ndt80*Δ, *rfx2*Δ, *sfu1*Δ, *zcf21*Δ, and *zcf35*Δ mutants, the complete coding region and flanking sequences of these genes were cloned as SacI–SacII fragments and inserted in place of the upstream flanking region of the corresponding deletion cassettes, generating plasmids pNDT80K1, pRFX2K1, pSFU1K1, pZCF21K1, and pZCF35K1.

### Construction of mNeonGreen-tagged strains

Strains expressing inducible *NDT80* (CAY13154/CAY13166) and *OFI1* (CAY12501/CAY 12525) genes were tagged with mNeonGreen, as described in Frazer et al. (64). Briefly, primer sets with flanking sequence of target genes were used to amplify the mNeonGreen cassette from the plasmid pRB2174. Transformants were selected on YPD medium supplemented with hygromycin (1 mg/mL) and sodium molybdate hydrate (600 µg/mL), and PCR genotyping was performed to confirm correct integration of the construct. A Zeiss fluorescence microscope was used to image the cells, and the fluorescence intensity of ~100 cells was measured using ImageJ.

### Media and growth conditions for switching assays

To induce gene expression from the Tet promoter, white cells of strains containing the TF library were grown overnight at 30°C in YPD medium, with or without 50 µg/mL doxycycline. The cultures were then diluted 10^−5^ and spread on modified Lee’s medium with or without 50 µg/mL doxycycline (recipe below). After incubation for 7 days at 22°C, colonies were analyzed using a stereoscope, and white and opaque (including mixed opaque/white colonies) were recorded to quantify switching frequencies for each strain. Induction of white-opaque switching by environmental signals was performed as described in the legend for Fig. 2.

Lee’s medium was prepared as previously described (48), using commercial Lee’s medium powder (Formedium, LEES0500) (see also Fig. S3). Three versions of Lee’s medium were used, as mentioned in Fig. S3. For screening of the TF library in WO-1 (Fig. 1), Lee’s^M^ medium was used, which included 70 mg/L arginine, 0.1 µM ZnSO_4_ (47), and 5 µg/mL phloxine B (70). For media-specific assays (Fig. 3), Lee’s^B^ medium was used, which included 45 mg/L arginine. For zinc supplementation assays, zinc sulfate heptahydrate stock solution in water (100 mg/mL) (Fisher Scientific, catalog: Z68-500) was added to the basic Lee’s medium (Lee’s^B^) to a final concentration of 400 µg/L (1.4 µM). Briefly, basic Lee’s medium was prepared by autoclaving 950 mL H_2_O with 20 g of agar and 17.3 g of Lee’s media powder from Formedium, followed by addition of 31.25 mL of 40% glucose, 5 mL of 1% histidine solution, 4.5 mL of 1% arginine solution, and 4 mL of 1.25% leucine solution. The pH of basic Lee’s medium was ~7.2.

SCD medium was prepared by autoclaving 850 mL H_2_O alongside 20 g of agar and 7 g of YNB (Difco, DF0919-07-3), followed by the addition of filter-sterilized solution containing 50 mL of 40% glucose, 4.5 mL of 1% uracil solution, 10.8 mL of 1.25% leucine solution, 4.5 mL of 1% histidine solution, 4.5 mL of 1% arginine solution, 2.5 mL of 10 mg/mL uridine solution, 1.7 g of powdered amino acid mix, and 75 mL of H_2_O. The pH of SCD medium was ~5.5 (see also Fig. S3).

Hybrid medium containing a 50:50 ratio of Lee’s and SCD media was prepared by autoclaving 850 mL H_2_O alongside 20 g of agar, 3.5 g of YNB, and 8.65 g of Lee’s medium powder (Formedium, LEES0500), followed by the addition of a filter-sterilized solution containing 40.625 mL of 40% glucose, 2.25 mL of 1% uracil solution, 7.4 mL of 1.25% leucine solution, 4.75 mL of 1% histidine solution, 2.25 mL of 1% arginine solution, 1.25 mL of 10 mg/mL uridine solution, 0.85 g of amino acid dropout powder, and 93.3 mL of H_2_O. The pH of the hybrid medium was ~6.4. Doxycycline (50 µg/mL) was added to plates for assays where TF expression was induced.

Incubation under anaerobic conditions was performed in an anaerobic jar (Anaerocult, Merck KGaA, Darmstadt, Germany) that generates an oxygen-free milieu in a CO_2_-rich atmosphere (18% CO_2_) within 1 h. In some experiments, an automatic counter (ProtoCOL 2 Count, Synbiosis, Cambridge, UK) was used to determine the frequency of white and opaque (including mixed white/opaque) colonies. The primary screening of the WO-1 TF library was performed in the Morschhäuser lab, except for Wor3 and Wor4, which were tested in the Bennett lab.

### *C. albicans* transformations

The *C. albicans* WO-1 library was generated by electroporation of cells (68) with the gel-purified inserts from the plasmids described above. The cassettes from the Tet-inducible transcription factor library were separated from the plasmid backbone by digestion with SacII/ApaI or SacII/KpnI (in some cases, a partial digest was required). Gene deletion and reinsertion cassettes were excised from the corresponding plasmids by SacI/ApaI digestion. Nourseothricin-resistant transformants were selected on YPD agar plates containing 200 µg/mL nourseothricin (Werner Bioagents, Jena, Germany) as described previously (68). The correct integration of each construct was confirmed by Southern hybridization using the flanking sequences as probes. In each case, two independent series of strains were generated and used for further analysis. The TF overexpression constructs introduced into the SC5314 background were transformed using the lithium acetate/polyethylene glycol (PEG) transformation protocol (64).

### Growth assays

Planktonic growth assays were performed in SCD pH 5.5 and Lee’s^B+Zn^ media (with and without doxycycline). White cells of each strain were inoculated into YPD medium and grown overnight at 30°C. Cultures were then diluted to 10^5^ cells/mL in the selective media, and 200 µL of each culture was inoculated in triplicate into a sterile, flat bottom, polystyrene microtiter plate. The growth rates of each strain were determined at 22°C by optical density measurements (OD_600_) at 15 min intervals for 48 h (SCD medium) and 72 h (Lee’s^B+Zn^ medium) using a Biotek Epoch 2 microtiter plate reader. The growth curves and statistical test were performed using GraphPad Prism 10.

### Nanostring analysis of gene expression

Briefly, cells were grown in triplicate in Lee’s^B+Zn^ medium, with or without doxycycline (50 µg/mL), for 12 h at 22°C. Cells were collected, and RNA was purified using the MasterPure Yeast RNA Purification Kit (LGC Biosearch Technologies) according to the manufacturer’s instructions. RNA samples were analyzed using the nCounter SPRINT Profiler at the genome core facility at Brown University, using a gene expression TagSet targeting 12 genes, including four housekeeping genes. For each Nanostring assay, 50 ng of RNA was hybridized with the Nanostring CodeSet and probes for each gene (A and B) at 67°C for 18 h in a preheated thermocycler. The hybridized samples were then transferred into an nCounter SPRINT cartridge, following the manufacturer’s instructions, and loaded into a nCounter SPRINT Profiler. The raw data files (.RCC) obtained were analyzed using nSolver software 4.0.

## SUPPLEMENTAL MATERIAL

Supplemental figures mbio.00581-25-s0001.pdf Figures S1 to S6.

Tables S1 and S2 mbio.00581-25-s0002.doc TF library.

Tables S3 to S5 mbio.00581-25-s0003.xlsx Switching data.

Table S6 mbio.00581-25-s0004.docx Strains used.

Tables S7 to S13 mbio.00581-25-s0005.xlsx Switching and growth data.

ASM does not own the copyrights to Supplemental Material that may be linked to, or accessed through, an article. The authors have granted ASM a non-exclusive, world-wide license to publish the Supplemental Material files. Please contact the corresponding author directly for reuse.
