# Supplementary material for: Transcriptional control of C. albicans white-opaque switching and modulation by environmental cues and strain background
Source: mBio. 2025 Apr 9;16(5):e00581-25. doi: 10.1128/mbio.00581-25 (PMC12077150; doi:10.1128/mbio.00581-25)
Supplement: Supplemental figures — Figures S1 to S6. [file mbio.00581-25-s0001.pdf]

Supplemental Figures

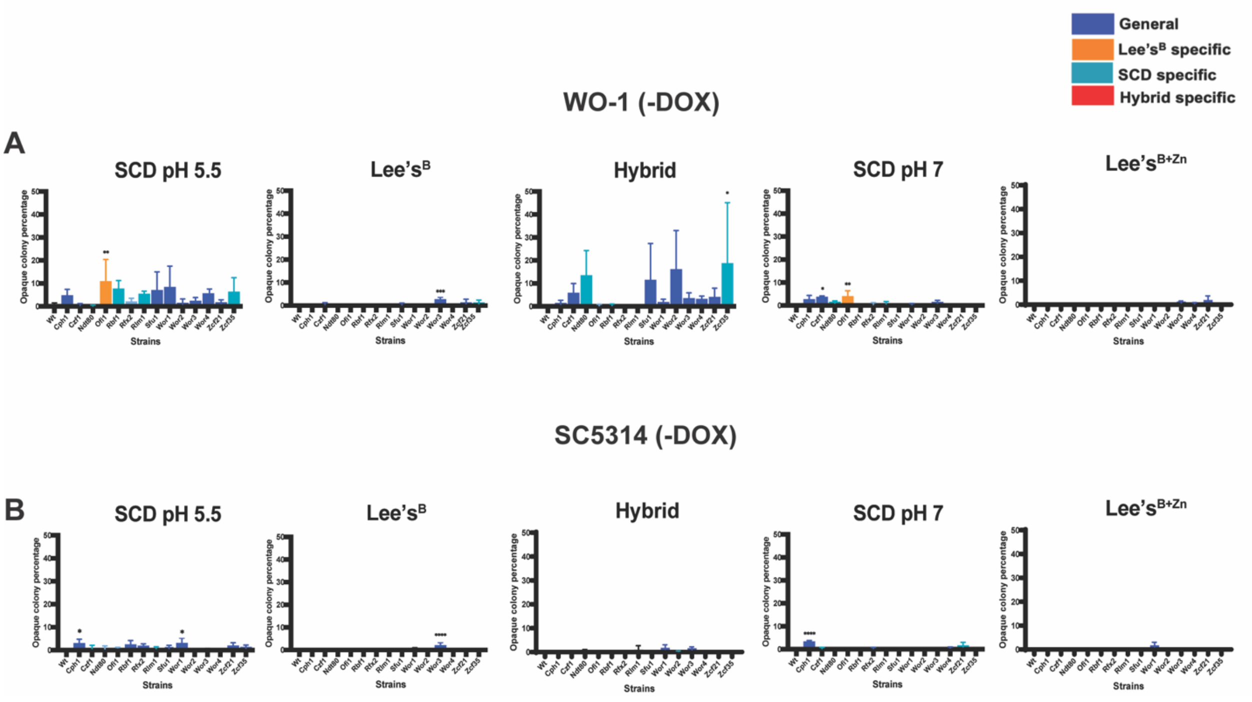

Figure S1. Effect of media and strain background on white-opaque switching White-opaque switching on SCD (pH 5.5 and pH 7), Lee's^B (+/-Zn) or hybrid media for WO-1 (**A-C**) and SC5314 (**D-F**) strains containing target TFs under non-inducing (no doxycycline) conditions (data in **Table S8**). The percentage of colonies with opaque (or mixed white/opaque colonies) phenotypes is provided. Overexpressed TFs that induced switching on SCD medium (when overexpressed) are highlighted in light blue, those that induced switching on Lee's^B are orange and those that induced switching on hybrid medium are red. Overexpressed TFs that induced switching on both Lee's^B and SCD media are shown in dark blue (**Figure 3**). Significant differences between the wild- type and each TF-expressing strains were calculated using a Dunnett's multicomparison test: *, P <0.05, **, P <0.01.

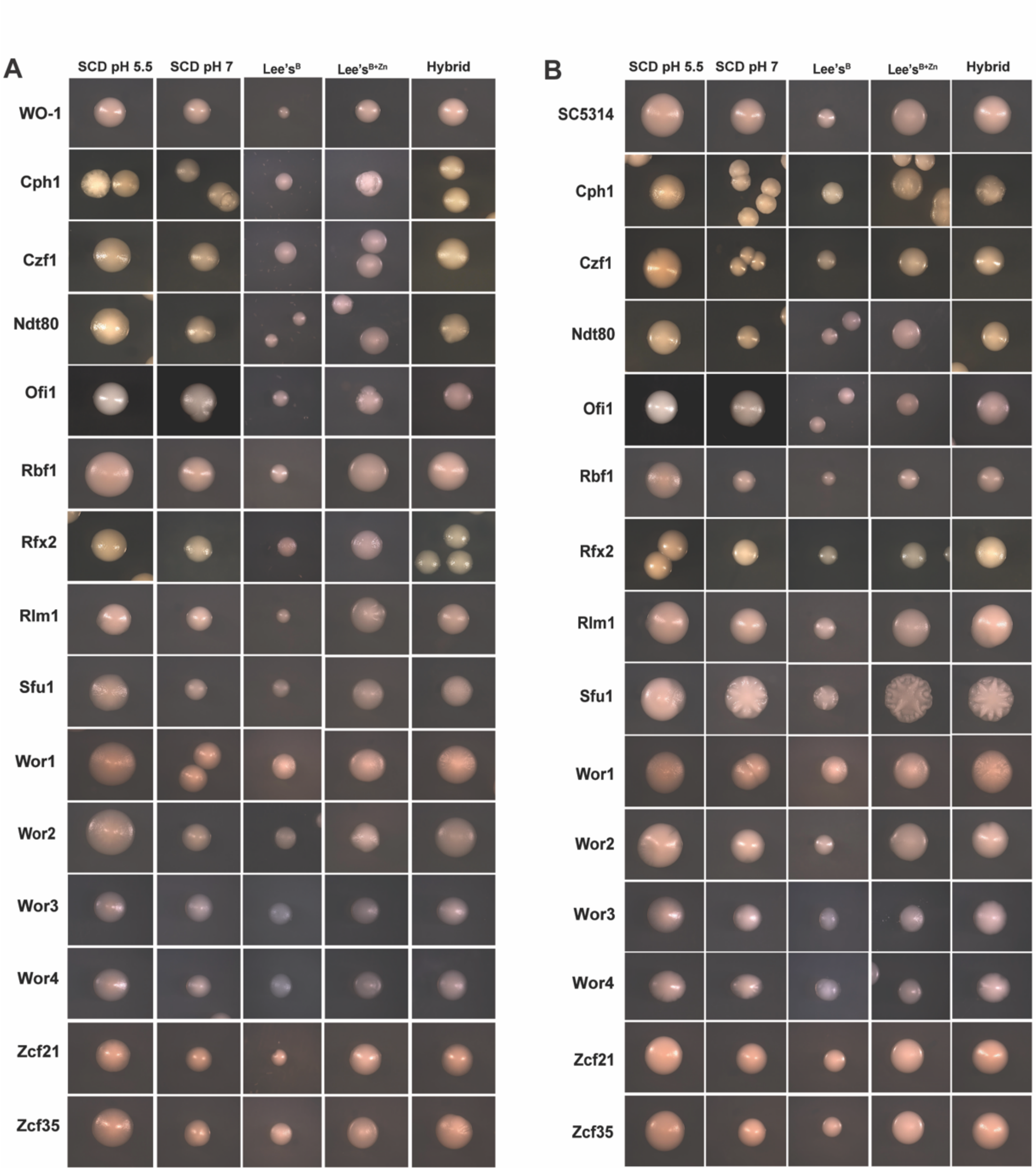

Figure S2. Colony morphologies under different media conditions. Colony phenotypes of WO-1 **(A)** and SC5314 **(B)** strains expressing target TFs (data in **Table S8**). Colonies were cultured with doxycycline present in the liquid precultures and subsequently incubated on agar plates containing doxycycline. Photographs were taken 7 days after incubation at 22°C. Some differences in coloration due to photographs taken on different days.

Lee's^{B} medium	SCD medium
LEES POWDER	***YNB POWDER***
Ammonium sulfate 5 g/L	Ammonium sulfate 5 g/L
Biotin 1000 μg/L	Biotin 2 μg/L
Magnesium sulfate 0.2 g/L	Magnesium sulfate 0.5 g/L
Potassium phosphate 0.2 g/L	Potassium phosphate 1 g/L
Sodium chloride 5 g/L	Sodium chloride 0.1 g/L
N/A	Calcium chloride 0.1 g/L
N/A	Calcium pantothenate 400 μg/L
N/A	Folic acid 2 μg/L
N/A	Inositol 2000 μg/L
N/A	Niacin 440 μg/L
N/A	p-Aminobenzoic Acid 200 μg/L
N/A	Pyridoxine hydrochloride 400 μg/L
N/A	Riboflavin 200 μg/L
N/A	Thiamine hydrochloride 400 μg/L
N/A	Boric acid 500 μg/L
N/A	Copper sulfate 40.0 μg/L
N/A	Potassium iodide 100.0 μg/L
N/A	Ferric chloride 200.0 μg/L
N/A	Manganese sulfate 400.0 μg/L
N/A	Sodium molybdate 200.0 μg/L
N/A	Zinc sulfate 400.0 μg/L
POWDERED AMINO ACIDS	***POWDERED AMINO ACIDS***
L-Methionine 0.1 g/L	L-Methionine 0.044 g/L
L-Lysine-HCl 0.1 g/L	L-Lysine-HCl 0.067 g/L
L-Phenylalanine 0.5 g/L	L-Phenylalanine 0.11 g/L
L-Threonine 0.5 g/L	L-Threonine 0.44 g/L
L-Leucine 1.3 g/L	N/A
L-Ornithine 0.0714 g/L	N/A
L-Proline 0.5 g/L	N/A
L-Alanine 0.5 g/L	N/A
N/A	L-Adenine-HCl 0.044 g/L
N/A	L-Trvotoohan 0.089 g/L
N/A	L-Tyrosine 0.067 g/L
N/A	L-Isoleucine 0.067 g/L
N/A	L-Valine 0.33 g/L
N/A	L-Serine 0.44 g/L
LIQUID INGREDIENTS	***LIQUID INGREDIENTS***
N/A	4.5 mL 1% Uracil (45 mg/L)
4 mL 1.25 % Leucine (50 mg/L)	10.8 mL 1.25% Leucine (135 mg/L)
5 mL 1 % Histidine (50 mg/L)	4.5 mL 1 % Histidine (45 mg/L)
4.5 mL 1 % Arginine (45 mg/L)	4.5 mL 1 % Arginine (45 mg/L)
N/A	2.5 mL Uridine (10 mg/mL)
950 mL H2O	925 mL H2O

Differences in Lee's media

- **Lee's^{M} (Modified Lee's):** Contains 70 mg/L of arginine and 0.1 μM zinc.
- **Lee's^{B} (Basic Lee's):** Contains 45 mg/L of arginine with no zinc added.
- **Lee's^{B+Zn} (Basic Lee's+ Zinc):** Contains 45 mg/L of arginine with 1.4 μM of zinc.

Figure S3. SCD and Lee's media recipes. The list of components in the SCD and Lee's^{B} media used for switching assays.

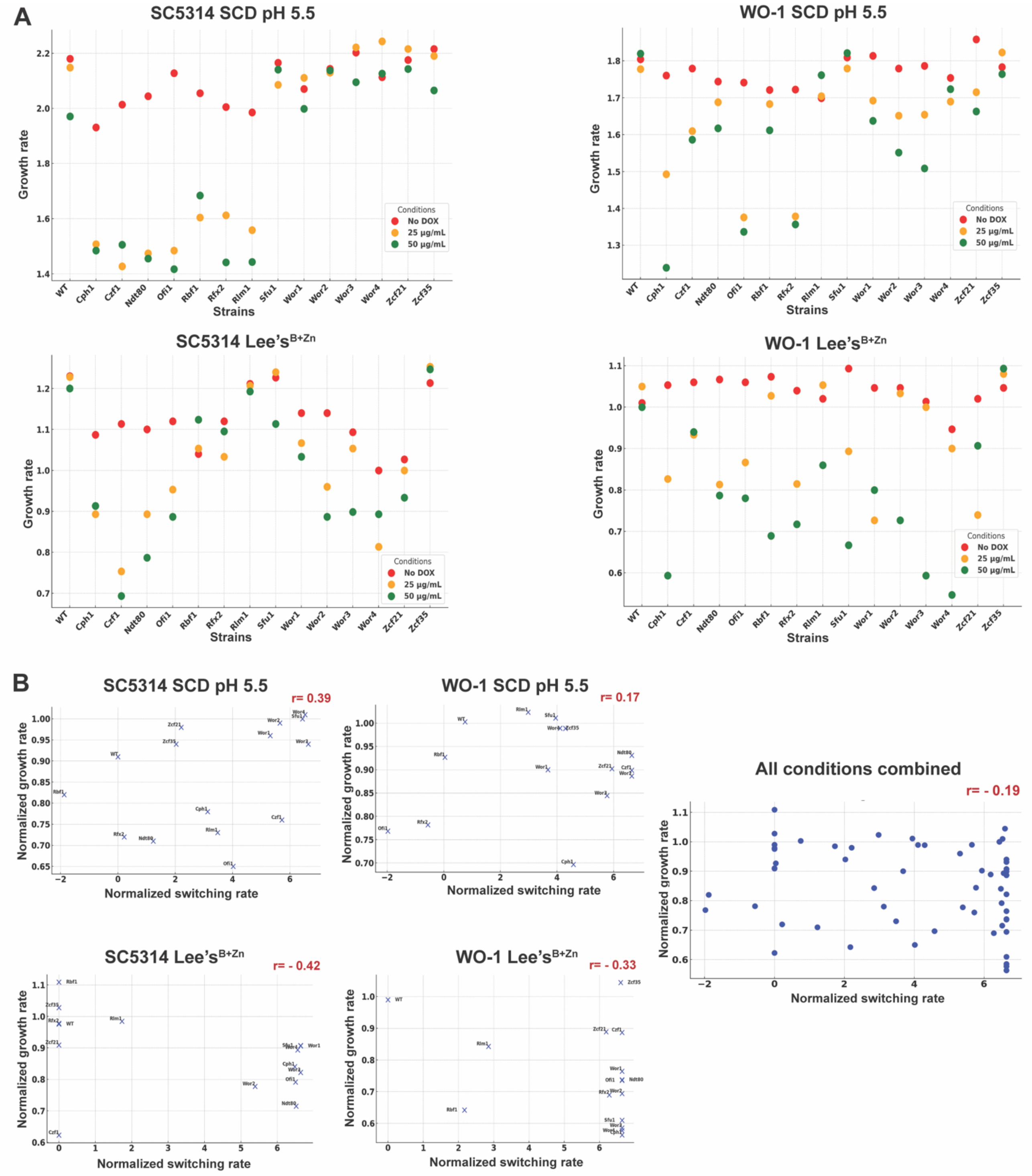
A
SC5314 SCD pH 5.5
WO-1 SCD pH 5.5
SC5314 Lee's B+Zn
WO-1 Lee's B+Zn
Growth rate
Strains
Conditions
No DOX
25 µg/mL
50 µg/mL
WT
Cph1
Czf1
Ndt80
Ofi1
Rbf1
Rfx2
Rlm1
Sfu1
Wor1
Wor2
Wor3
Wor4
Zcf21
Zcf35
B
r= 0.39
r= 0.17
r= - 0.42
r= - 0.33
All conditions combined
r= - 0.19
Normalized growth rate
Normalized switching rate

Figure S4. Analysis of growth rates and switching frequencies in TF-expressing strains

(A) Growth rates (Maximum velocity values) of control and TF expressing strains are shown in SCD and Lee's^{B+Zn} medium with and without doxycycline (25 or 50 μg/mL). Pink, yellow and green dots represent no doxycycline, 25 and 50 μg/mL of doxycycline respectively (data in **Table S11**).

(B) Comparison of normalized growth rates (**Table S8**) and switching rates (**Table S11**) of control and TF-overexpressing strains in SCD and Lee's^{B+Zn} medium with and without 50 μg/mL doxycycline. The values were normalized to the no doxycycline control. The x axis represents the normalized switching rates in Log_2 scale and y axis represents the normalized growth rates in linear scale. The Pearson correlation (r) values are indicated in each panel in red color.

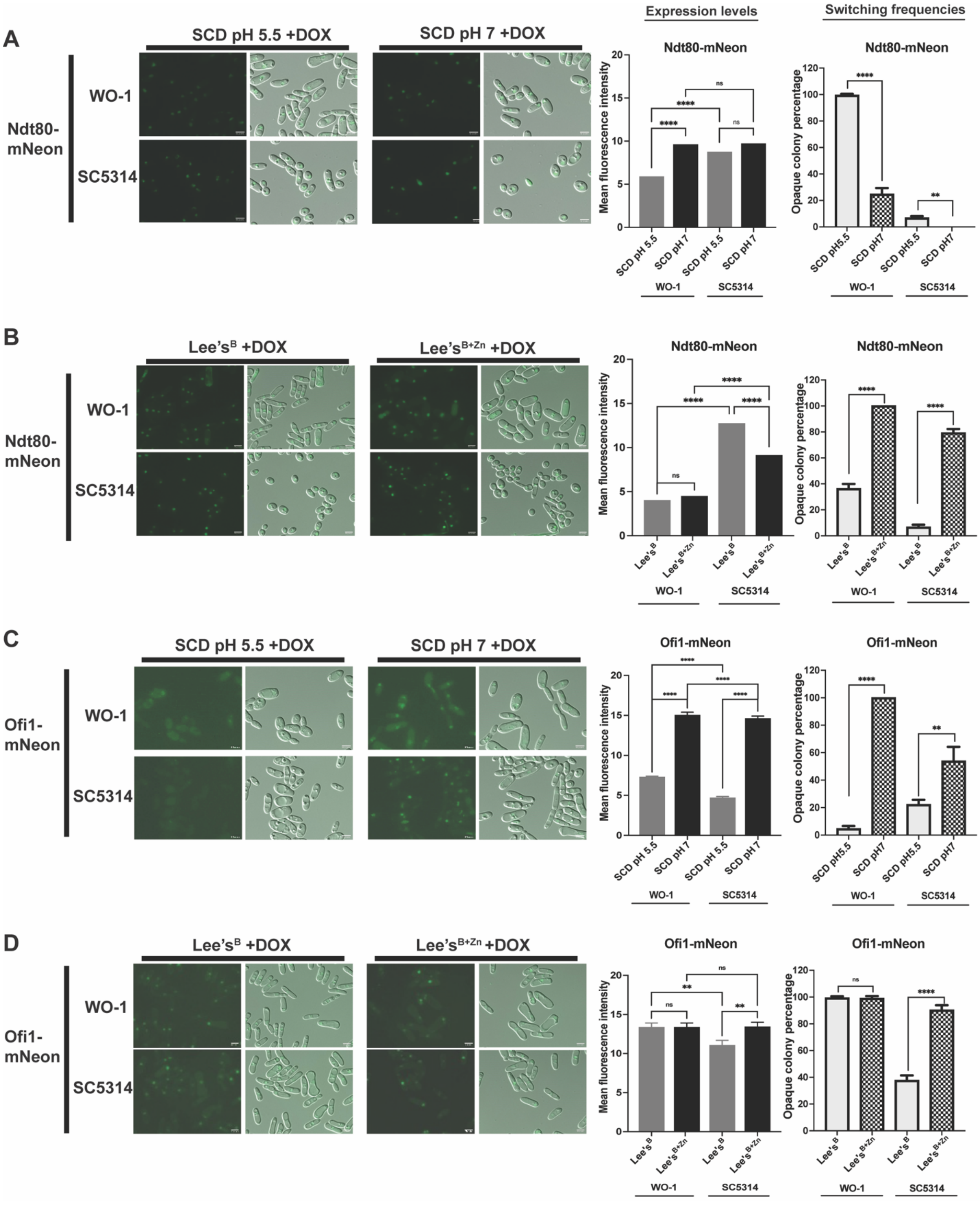
A
SCD pH 5.5 +DOX
SCD pH 7 +DOX
Ndt80-mNeon
WO-1
SC5314
Expression levels
Switching frequencies
Ndt80-mNeon
Mean fluorescence intensity
Opaque colony percentage
ns
****
**
SCD pH 5.5
SCD pH 7
SCD pH5.5
SCD pH7
B
Lee's^{B} +DOX
Lee's^{B+Zn} +DOX
Lee's^{B}
Lee's^{B+Zn}
C
Ofi1-mNeon
D

Figure S5. Effect of strain and media conditions on induced TF expression. Switching assays were performed on SCD medium (pH 5.5 or pH 7) and Lee's^{B} medium (+/-Zn) for strains ectopically expressing Tet-induced Ndt80-mNeon (**A** and **B**) and Tet-induced Ofi1-mNeon (**C** and **D**). After 7 days, cells were imaged to measure fluorescence intensity in 100 cells per strain using ImageJ. In **A** and **C**, the gray and black bars represent the mean fluorescence intensity in the SCD medium at pH 5.5 and pH 7, respectively. In **B** and **D**, the gray and black bars represent the mean fluorescence intensity in the Lee's^{B} and Lee's^{B+Zn} media, respectively (**Table S12B**). For switching assays, solid white bars represent the switching frequencies in SCD pH 5.5 and Lee's^{B} media, while patterned white bars represent switching frequencies in SCD pH 7 and Lee's^{B+Zn} media. Significant differences between pair of means were calculated using Tukey's multicomparison test: ns, P >0.05, **, P <0.01. ***, P <0.001, ****, P <0.0001.

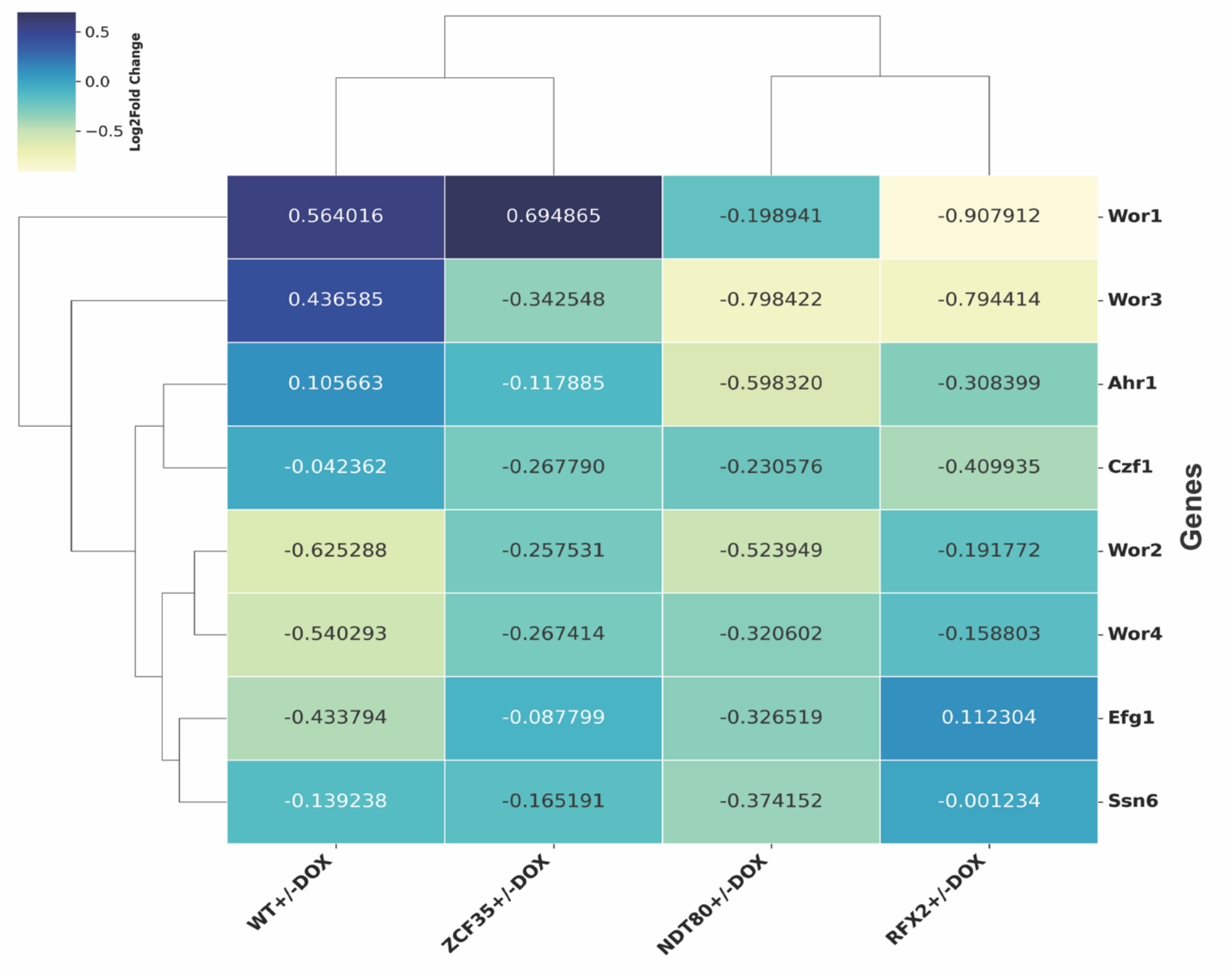

Figure S6. Impact of Tet-induced TFs on the expression of core white-opaque network TFs. RNA expression levels of core network TF genes were quantified in control and Tet-induced TFs strains (Ndt80, Rfx2 and Zcf35). White cells were cultured for 12 h in Lee's^{B+Zn} medium with or without 50 µg/mL doxycycline at 22°C. The heat map shows hierarchal clustering of gene expression data. Fold-change expression values were calculating by dividing the normalized expression values of each strain grown with doxycycline by those grown without the doxycycline (**Table S14**). The data is representative of three replicates for each strain. Upper (blue) and lower bounds (yellow) correspond to $\log_2$ fold change values of 1 and -1, respectively.
