# Supplementary material for: Transcriptional control of C. albicans white-opaque switching and modulation by environmental cues and strain background
Source: mBio. 2025 Apr 9;16(5):e00581-25. doi: 10.1128/mbio.00581-25 (PMC12077150; doi:10.1128/mbio.00581-25)
Supplement: Tables S1 and S2 — TF library. [file mbio.00581-25-s0002.doc]

**Table S1. Tet-inducible *Candida albicans* transcription factor library**

| **No.** | **orf19 no. ^a^** | **CGD name** | **Library name ^b^** | **Length (bp)** | **Difference from current annotation ^c^** | **Vector** |
| --- | --- | --- | --- | --- | --- | --- |
| 1 | orf19.6124 | *ACE2* |  | 2352 |  | pNIM6 |
| 2 | orf19.2331 | *ADA2* |  | 1338 |  | pNIM6 |
| 3 | orf19.2752 | *ADR1* |  | 4260 |  | pNIM6 |
| 4 | orf19.4766 | *ARG81* |  | 3300 |  | pNIM6 |
| 5 | orf19.2748 | *ARG83* |  | 2925 |  | pNIM6 |
| 6 | orf19.3012 | *ARO80* |  | 3198 |  | pNIM6 |
| 7 | orf19.3027 | *ARP7* | *ARP7* | 1287 | N-terminal 150 bp not included in assembly 21 | pNIM6 |
| 8 | orf19.2507 | *ARP9* |  | 1455 |  | pNIM6 |
| 9 | orf19.5343 | *ASH1* |  | 1350 |  | pNIM6 |
| 10 | orf19.3964 | *ASH2* |  | 1401 |  | pNIM6 |
| 11 | orf19.4043 |  | *ASK10* | 2259 |  | pNIM6 |
| 12 | orf19.173 |  | *AZF1* | 3303 |  | pNIM6 |
| 13 | orf19.3809 | *BAS1* |  | 2217 |  | pNIM6 |
| 14 | orf19.723 | *BCR1* |  | 2202 |  | pNIM6 |
| 15 | orf19.978 | *BDF1* |  | 2130 |  | pNIM6 |
| 16 | orf19.976 | *BRE1* |  | 2046 |  | pNIM6 |
| 17 | orf19.6649 | *BRF1* |  | 1662 |  | pNIM6 |
| 18 | orf19.1623 | *CAP1* |  | 1500 |  | pNIM6 |
| 19 | orf19.1135 | *CAS1* |  | 2436 |  | pNIM6 |
| 20 | orf19.4670 | *CAS5* |  | 2466 |  | pNIM6 |
| 21 | orf19.5097 | *CAT8* |  | 3171 |  | pNIM6 |
| 22 | orf19.2876 | *CBF1* |  | 672 | N-terminal extension with intron in assembly 21 | pNIM6 |
| 23 | orf19.4542 |  | *CCL1* | 1188 |  | pNIM6 |
| 24 | orf19.4433 | *CPH1* | *CPH1*^K652N^ | 1965 |  | pNIM6 |
| 25 | orf19.1187 | *CPH2* |  | 2562 |  | pNIM6 |
| 26 | orf19.7359 | *CRZ1* |  | 2196 |  | pNIM6 |
| 27 | orf19.2356 | *CRZ2* |  | 1554 |  | pNIM6 |
| 28 | orf19.3794 | *CSR1* |  | 2124 | N-terminal 285 bp not included in assembly 21 | pNIM6 |
| 29 | orf19.5467 | *TLO7* |  | 510 |  | pNIM6 |
| 30 | orf19.4647 | *HAP3* | *HAP3*^1109^ | 1109 |  | pNIM6 |
| 31 | orf19.632 |  | *CTA2* | 1212 | merged with orf19.1808 in assembly 21 | pNIM6 |
| 32 | orf19.1032 | *SKO1* |  | 1841 |  | pNIM6 |
| 33 | orf19.1166 | *CTA3* |  | 3993 |  | pNIM6 |
| 34 | orf19.3102 | *CTA6* |  | 642 |  | pNIM6 |
| 35 | orf19.3315 | *CTA9* |  | 1284 |  | pNIM6 |
| 36 | orf19.7127 | *TLO16* |  | 528 |  | pNIM6 |
| 37 | orf19.3401 | *CTA1* |  | 657 |  | pNIM6 |
| 38 | orf19.1925 | *TLO5* |  | 531 |  | pNIM6 |
| 39 | orf19.4054 | *CTA24* |  | 759 |  | pNIM6 |
| 40 | orf19.4590 | *RFX2* |  | 3336 |  | pNIM6 |
| 41 | orf19.7680 | *CTA26* |  | 822 |  | pNIM6 |
| 42 | orf19.6337 | *TLO13* |  | 945 | only N-terminal 531 bp and no intron in assembly 21 | pNIM6 |
| 43 | orf19.7374 | *CTA4* |  | 3051 |  | pNIM6 |
| 44 | orf19.4775 | *CTA8* |  | 2280 |  | pNIM6 |
| 45 | orf19.1499 | *CTF1* |  | 3420 |  | pNIM6 |
| 46 | orf19.5001 | *CUP2* |  | 1776 |  | pNIM6 |
| 47 | orf19.6514 | *CUP9* |  | 1035 |  | pNIM6 |
| 48 | orf19.5849 | *CWT1* |  | 1737 |  | pNIM6 |
| 49 | orf19.3127 | *CZF1* |  | 1158 |  | pNIM1 |
| 50 | orf19.3252 | *DAL81* |  | 2637 |  | pNIM6 |
| 51 | orf19.8243 | *EFG1* |  | 1650 |  | pNIM6 |
| 52 | orf19.5498 | *EFH1* |  | 2163 |  | pNIM6 |
| 53 | orf19.3722 |  | *FAP1* | 2550 |  | pNIM6 |
| 54 | orf19.6817 | *FCR1* |  | 1554 |  | pNIM6 |
| 55 | orf19.2054 | *FGR15* |  | 1557 |  | pNIM6 |
| 56 | orf19.5729 | *FGR17* |  | 1965 |  | pNIM6 |
| 57 | orf19.6680 | *FGR27* |  | 2364 |  | pNIM6 |
| 58 | orf19.5389 | *FKH2* |  | 1581 | C-terminal extension with intron in assembly 21 | pNIM6 |
| 59 | orf19.1093 | *FLO8* |  | 2448 | N-terminal 75 bp not included in assembly 21 | pNIM6 |
| 60 | orf19.5338 | *GAL4* |  | 786 |  | pNIM6 |
| 61 | orf19.1275 | *GAT1* |  | 2067 |  | pNIM1 |
| 62 | orf19.4056 | *BRG1* | *GAT2*^1341^ | 1341 | shorter C-terminus due to frameshift in assembly 21 | pNIM6 |
| 63 | orf19.1358 | *GCN4* |  | 969 |  | pNIM6 |
| 64 | orf19.3182 | *GIS2* |  | 528 |  | pNIM6 |
| 65 | orf19.3912 | *GLN3* |  | 2049 |  | pNIM1 |
| 66 | orf19.1150 |  | *GLN32* | 1419 |  | pNIM6 |
| 67 | orf19.720 | *GST3* |  | 648 |  | pNIM6 |
| 68 | orf19.6393 |  | *GTS1* | 1251 |  | pNIM6 |
| 69 | orf19.2842 | *GZF3* |  | 2133 |  | pNIM6 |
| 70 | orf19.2432 | *HAC1* |  | 1074 | altered C-terminus with intron in assembly 21 | pNIM6 |
| 71 | orf19.3190 | *HAL9* |  | 3033 |  | pNIM6 |
| 72 | orf19.4647 | *HAP3* | *HAP3*^882^ | 882 | N-terminal extension with intron in assembly 21 | pNIM6 |
| 73 | orf19.517 | *HAP31* |  | 318 |  | pNIM6 |
| 74 | orf19.740 | *HAP41* |  | 1146 | N-terminal extension with intron in assembly 21 | pNIM1 |
| 75 | orf19.1481 | *HAP42* |  | 2517 |  | pNIM6 |
| 76 | orf19.681 | *HAP43* |  | 1905 |  | pNIM1 |
| 77 | orf19.1973 | *HAP5* |  | 1047 |  | pNIM6 |
| 78 | orf19.4853 | *HCM1* |  | 1740 |  | pNIM6 |
| 79 | orf19.1715 | *IRO1* |  | 1791 |  | pNIM6 |
| 80 | orf19.3736 | *KAR4* |  | 1110 |  | pNIM6 |
| 81 | orf19.4225 | *LEU3* |  | 2955 |  | pNIM6 |
| 82 | orf19.3505 | *SLM2* | *LIT1* | 2397 |  | pNIM6 |
| 83 | orf19.5548 | *LYS14* |  | 1779 |  | pNIM6 |
| 84 | orf19.4778 | *LYS142* |  | 2070 |  | pNIM6 |
| 85 | orf19.4776 | *LYS143* |  | 2631 |  | pNIM6 |
| 86 | orf19.5380 | *LYS144* |  | 2586 |  | pNIM6 |
| 87 | orf19.7068 | *MAC1* |  | 1296 |  | pNIM6 |
| 88 | orf19.5855 | *MBP1* |  | 2559 |  | pNIM6 |
| 89 | orf19.7025 | *MCM1* |  | 789 |  | pNIM6 |
| 90 | orf19.1706 | *MET18* |  | 3264 |  | pNIM6 |
| 91 | orf19.7046 | *MET28* |  | 522 |  | pNIM6 |
| 92 | orf19.5312 | *MET4* | *MET5* | 1158 |  | pNIM6 |
| 93 | orf19.4318 | *MIG1* |  | 1725 |  | pNIM6 |
| 94 | orf19.5326 | *MIG2* | *MIG2* | 810 |  | pNIM6 |
| 95 | orf19.4752 | *MSN4* |  | 2274 |  | pNIM6 |
| 96 | orf19.6309 | *MSS11* |  | 1908 |  | pNIM6 |
| 97 | orf19.3201 | *MTLA1* |  | 963 |  | pNIM6 |
| 98 | orf19.3200 | *MTLA2* |  | 661 |  | pNIM6 |
| 99 | orf19.10712 | *MTLALPHA1* |  | 582 |  | pNIM6 |
| 100 | orf19.10708 | *MTLALPHA2* |  | 620 |  | pNIM6 |
| 101 | orf19.5825 | *NCB2* | *NCB2* | 450 |  | pNIM6 |
| 102 | orf19.339 | *NDE1* |  | 1725 |  | pNIM6 |
| 103 | orf19.2119 | *NDT80* |  | 1503 |  | pNIM6 |
| 104 | orf19.513 | *RON1* | *NDT802* | 1473 |  | pNIM6 |
| 105 | orf19.3023 | *NGG1* |  | 1884 |  | pNIM6 |
| 106 | orf19.2379 | *NOT4* |  | 1668 | N-terminal extension with intron in assembly 21 | pNIM6 |
| 107 | orf19.7150 | *NRG1* |  | 933 |  | pNIM1 |
| 108 | orf19.5268 | *MED10* | *NUT2* | 513 |  | pNIM6 |
| 109 | orf19.1543 | *OPI1* |  | 1293 |  | pNIM6 |
| 110 | orf19.4863 | *PDC2* |  | 2511 |  | pNIM6 |
| 111 | orf19.4000 | *GRF10* | *PHO2* | 2058 |  | pNIM6 |
| 112 | orf19.1253 | *PHO4* | *PHO4* | 1980 |  | pNIM6 |
| 113 | orf19.3986 | *PPR1* |  | 2595 |  | pNIM6 |
| 114 | orf19.7119 | *RAD3* | *RAD3* | 2298 |  | pNIM6 |
| 115 | orf19.1773 | *RAP1* |  | 1293 |  | pNIM6 |
| 116 | orf19.5558 | *RBF1* |  | 1584 |  | pNIM6 |
| 117 | orf19.2230 |  | *RDS3* | 360 |  | pNIM6 |
| 118 | orf19.2823 | *RFG1* |  | 1806 |  | pNIM6 |
| 119 | orf19.2747 | *RGT1* |  | 3078 |  | pNIM6 |
| 120 | orf19.7247 | *RIM101* | *RIM101*^ΔC462^ | 1389 |  | pNIM6 |
| 121 | orf19.4662 | *RLM1* |  | 1836 |  | pNIM6 |
| 122 | orf19.4438 | *RME1* |  | 1524 |  | pNIM6 |
| 123 | orf19.2743 |  | *RPH1* | 1821 |  | pNIM6 |
| 124 | orf19.1069 | *RPN4* |  | 1611 |  | pNIM6 |
| 125 | orf19.519 |  | *RRN10* | 588 |  | pNIM6 |
| 126 | orf19.718 | *RRN11* |  | 2070 |  | pNIM6 |
| 127 | orf19.1923 | *RRN3* |  | 1671 |  | pNIM6 |
| 128 | orf19.4420 |  | *RRN6* | 2403 |  | pNIM6 |
| 129 | orf19.1589 |  | *RRN7* | 1314 |  | pNIM6 |
| 130 | orf19.4722 | *RTG1* | *RTG1* | 825 |  | pNIM6 |
| 131 | orf19.2315 | *RTG3* | *RTG3* | 1563 |  | pNIM6 |
| 132 | orf19.3753 | *SEF1* |  | 2754 |  | pNIM6 |
| 133 | orf19.1926 | *SEF2* |  | 1986 |  | pNIM6 |
| 134 | orf19.1755 | *SET2* |  | 2535 |  | pNIM6 |
| 135 | orf19.3665 | *SET6* | *SET6* | 1140 |  | pNIM6 |
| 136 | orf19.454 | *SFL1* |  | 2418 |  | pNIM6 |
| 137 | orf19.4869 | *SFU1* |  | 1554 |  | pNIM6 |
| 138 | orf19.6011 | *SIN3* |  | 4639 |  | pNIM6 |
| 139 | orf19.971 | *SKN7* |  | 1680 |  | pNIM6 |
| 140 | orf19.487 | *SPT14* |  | 1359 |  | pNIM6 |
| 141 | orf19.422 | *SPT20* |  | 2244 |  | pNIM6 |
| 142 | orf19.7622 | *SPT3* |  | 987 |  | pNIM6 |
| 143 | orf19.7572 | *SPT7* |  | 3924 |  | pNIM6 |
| 144 | orf19.4312 | *SPT8* | *SPT8* | 2307 |  | pNIM6 |
| 145 | orf19.1457 |  | *SSL1* | 1419 |  | pNIM6 |
| 146 | orf19.6798 | *SSN6* |  | 3243 |  | pNIM6 |
| 147 | orf19.6734 | *TCC1* | *TCC1*^R598H^ | 2211 |  | pNIM6 |
| 148 | orf19.7355 | *SSN8* |  | 1305 |  | pNIM6 |
| 149 | orf19.3308 | *STB5* |  | 1923 |  | pNIM6 |
| 150 | orf19.6173 | *STD1* |  | 1008 |  | pNIM6 |
| 151 | orf19.4961 | *STP2* | *STP2^ΔN100^* | 1449 |  | pNIM6 |
| 152 | orf19.5917 | *STP1* | *STP1*^ΔN61^ | 1131 |  | pNIM1 |
| 153 | orf19.909 | *STP4* |  | 1131 |  | pNIM6 |
| 154 | orf19.3059 | *SUA71* |  | 1035 |  | pNIM6 |
| 155 | orf19.3519 | *SUA72* |  | 1041 |  | pNIM6 |
| 156 | orf19.7319 | *SUC1* |  | 1506 |  | pNIM6 |
| 157 | orf19.5657 | *SWI1* |  | 2964 |  | pNIM6 |
| 158 | orf19.4488 |  | *SWI3* | 2934 |  | pNIM6 |
| 159 | orf19.4545 | *SWI4* |  | 2994 | N-terminal extension with intron in assembly 21 | pNIM6 |
| 160 | orf19.4725 | *SWI6* |  | 2190 |  | pNIM6 |
| 161 | orf19.3188 | *TAC1* |  | 2946 |  | pNIM6 |
| 162 | orf19.6193 | *TAF145* |  | 3777 |  | pNIM6 |
| 163 | orf19.5174 | *TAF19* |  | 498 |  | pNIM6 |
| 164 | orf19.1885 | *TAF4* |  | 1092 |  | pNIM6 |
| 165 | orf19.1693 | *CAS4* |  | 8493 |  | pNIM6 |
| 166 | orf19.1837 | *TBP1* |  | 717 |  | pNIM6 |
| 167 | orf19.6985 | *TEA1* |  | 2703 |  | pNIM6 |
| 168 | orf19.5908 | *TEC1* |  | 2232 |  | pNIM6 |
| 169 | orf19.4851 | *TFA1* |  | 1185 |  | pNIM6 |
| 170 | orf19.4601 |  | *TFC1* | 1518 |  | pNIM6 |
| 171 | orf19.3833 |  | *TFC3* | 4212 |  | pNIM6 |
| 172 | orf19.2325 |  | *TFC6* | 1554 |  | pNIM6 |
| 173 | orf19.4585 | *TFG1* |  | 1827 |  | pNIM6 |
| 174 | orf19.889 | *THI20* |  | 1647 |  | pNIM6 |
| 175 | orf19.4625 | *TOA2* |  | 393 |  | pNIM6 |
| 176 | orf19.668 | *TOS4* | *TOS4* | 1479 |  | pNIM6 |
| 177 | orf19.6109 | *TUP1* |  | 1533 |  | pNIM6 |
| 178 | orf19.4941 | *TYE7* |  | 810 |  | pNIM6 |
| 179 | orf19.7570 | *UGA3* |  | 2412 |  | pNIM6 |
| 180 | orf19.7317 | *UGA33* |  | 1449 |  | pNIM6 |
| 181 | orf19.1822 | *UME6* |  | 2538 |  | pNIM6 |
| 182 | orf19.2745 | *UME7* |  | 1377 |  | pNIM6 |
| 183 | orf19.391 | *UPC2* |  | 2139 |  | pNIM6 |
| 184 | orf19.155 | *URE2* |  | 1035 |  | pNIM6 |
| 185 | orf19.1035 | *WAR1* |  | 2844 |  | pNIM6 |
| 186 | orf19.5210 |  | *XBP1* | 1734 |  | pNIM6 |
| 187 | orf19.3193 | *FCR3* |  | 1200 |  | pNIM6 |
| 188 | orf19.175 |  |  | 1551 |  | pNIM6 |
| 189 | orf19.255 | *ZCF1* |  | 2517 |  | pNIM6 |
| 190 | orf19.2280 | *ZCF10* |  | 2748 |  | pNIM6 |
| 191 | orf19.2423 | *ZCF11* |  | 1785 |  | pNIM6 |
| 192 | orf19.2623 | *ECM22* |  | 2583 |  | pNIM6 |
| 193 | orf19.2646 | *ZCF13* | *HAP1* | 3729 |  | pNIM6 |
| 194 | orf19.2647 | *ZCF14* |  | 1581 |  | pNIM6 |
| 195 | orf19.2753 | *ZCF15* |  | 2835 |  | pNIM6 |
| 196 | orf19.2808 | *ZCF16* |  | 3237 |  | pNIM6 |
| 197 | orf19.3305 | *ZCF17* |  | 2217 |  | pNIM6 |
| 198 | orf19.3405 | *ZCF18* |  | 1593 |  | pNIM6 |
| 199 | orf19.3876 | *ZCF19* |  | 1860 |  | pNIM6 |
| 200 | orf19.431 | *ZCF2* |  | 2328 |  | pNIM6 |
| 201 | orf19.4145 | *ZCF20* |  | 3258 |  | pNIM6 |
| 202 | orf19.4166 | *ZCF21* |  | 1884 |  | pNIM6 |
| 203 | orf19.4251 | *ZCF22* |  | 2100 |  | pNIM6 |
| 204 | orf19.4450 | *ZCF23* |  | 1701 |  | pNIM6 |
| 205 | orf19.4524 | *ZCF24* |  | 2184 |  | pNIM6 |
| 206 | orf19.4568 | *ZCF25* |  | 2418 |  | pNIM6 |
| 207 | orf19.4573 | *ZCF26* |  | 2493 |  | pNIM6 |
| 208 | orf19.4649 | *ZCF27* |  | 3573 |  | pNIM6 |
| 209 | orf19.4767 | *ZCF28* |  | 741 |  | pNIM6 |
| 210 | orf19.5133 | *ZCF29* |  | 3318 |  | pNIM6 |
| 211 | orf19.1168 | *ZCF3* |  | 588 |  | pNIM6 |
| 212 | orf19.5251 | *ZCF30* |  | 2400 |  | pNIM6 |
| 213 | orf19.5924 | *ZCF31* |  | 3393 |  | pNIM6 |
| 214 | orf19.5940 | *ZCF32* |  | 2151 |  | pNIM6 |
| 215 | orf19.5992 | *WOR2* |  | 1341 |  | pNIM6 |
| 216 | orf19.6182 | *MRR2* | *ZCF34* | 2133 |  | pNIM6 |
| 217 | orf19.7371 | *ZCF35* |  | 3309 |  | pNIM6 |
| 218 | orf19.7372 | *MRR1* |  | 3327 |  | pNIM1 |
| 219 | orf19.7381 | *AHR1* | *ZCF37* | 1875 |  | pNIM6 |
| 220 | orf19.7518 | *ZCF38* |  | 1749 |  | pNIM6 |
| 221 | orf19.7583 | *ZCF39* |  | 2046 |  | pNIM6 |
| 222 | orf19.1227 | *ZCF4* |  | 594 |  | pNIM6 |
| 223 | orf19.1255 | *ZCF5* |  | 2916 |  | pNIM6 |
| 224 | orf19.1497 | *ZCF6* |  | 1110 |  | pNIM6 |
| 225 | orf19.1685 | *ZCF7* |  | 1347 |  | pNIM6 |
| 226 | orf19.1718 | *ZCF8* |  | 2748 |  | pNIM6 |
| 227 | orf19.2077 | *ZCF9* |  | 2505 |  | pNIM6 |
| 228 | orf19.3187 | *ZNC1* |  | 2769 |  | pNIM6 |
| 229 | orf19.3300 | *ZPR1* |  | 1530 |  | pNIM6 |
| 230 | orf19.274 | *TFC4* |  | 3180 |  | pNIM6 |
| 231 | orf19.470 | *TAF12L* |  | 2226 |  | pNIM6 |
| 232 | orf19.536 |  |  | 2394 |  | pNIM6 |
| 233 | orf19.567 | *TFB3* |  | 1143 |  | pNIM6 |
| 234 | orf19.735 |  |  | 1467 | merged with orf19.6193 in assembly 21 | pNIM6 |
| 235 | orf19.736 | *SRB8* | 736 | 1671 | C-terminal extension due to frameshift in assembly 21 | pNIM6 |
| 236 | orf19.794 | *SSN3* | 794 | 1815 |  | pNIM6 |
| 237 | orf19.798 | *TAF14* |  | 711 | N-terminal extension with intron in assembly 21 | pNIM6 |
| 238 | orf19.861 | *CAP4* |  | 531 |  | pNIM6 |
| 239 | orf19.2528 | *BDP1* |  | 1989 |  | pNIM6 |
| 240 | orf19.2612 |  |  | 753 |  | pNIM6 |
| 241 | orf19.2674 |  |  | 1029 |  | pNIM6 |
| 242 | orf19.2682 | *TOA1* |  | 828 |  | pNIM6 |
| 243 | orf19.2693 | *GST2* | 2693 | 660 |  | pNIM6 |
| 244 | orf19.2736 | *HFL2* |  | 441 |  | pNIM6 |
| 245 | orf19.2857 | *SSL2* |  | 2532 |  | pNIM6 |
| 246 | orf19.2961 |  |  | 1194 |  | pNIM6 |
| 247 | orf19.3018 | *SPP1* | 3018 | 1221 |  | pNIM6 |
| 248 | orf19.3088 |  |  | 540 |  | pNIM6 |
| 249 | orf19.3121 | *GST1* | 3121 | 738 |  | pNIM6 |
| 250 | orf19.3242 | *TAF10* |  | 735 |  | pNIM6 |
| 251 | orf19.684 | *PCF11* |  | 1548 |  | pNIM6 |
| 252 | orf19.3407 | *RAD18* |  | 1137 |  | pNIM6 |
| 253 | orf19.3683 | *AGE3* | 3683 | 1128 |  | pNIM6 |
| 254 | orf19.3835 |  |  | 1959 | merged with orf19.3833 in assembly 21 | pNIM6 |
| 255 | orf19.3928 |  |  | 1143 |  | pNIM6 |
| 256 | orf19.3969 | *SFL2* | 3969 | 2133 |  | pNIM6 |
| 257 | orf19.4194 | *TFB4* |  | 1092 |  | pNIM6 |
| 258 | orf19.4295 | *HIR2* |  | 3054 |  | pNIM6 |
| 259 | orf19.4342 | *SUT1* |  | 1083 |  | pNIM6 |
| 260 | orf19.4388 | *TAF3* |  | 1095 |  | pNIM6 |
| 261 | orf19.4882 | *TFA2* |  | 855 |  | pNIM6 |
| 262 | orf19.4972 | *OFI1* |  | 1947 |  | pNIM6 |
| 263 | orf19.4998 | *ROB1* | 4998 | 2091 | N-terminal extension with intron in assembly 21 | pNIM6 |
| 264 | orf19.5026 | *ZMS1* |  | 3534 |  | pNIM6 |
| 265 | orf19.5068 | *IRE1* | 5068 | 3666 |  | pNIM6 |
| 266 | orf19.5297 | *TFB1* |  | 1893 |  | pNIM6 |
| 267 | orf19.5388 | *SWD1* | 5388 | 1344 |  | pNIM6 |
| 268 | orf19.5846 |  |  | 1485 |  | pNIM6 |
| 269 | orf19.5853 |  |  | 531 | merged with orf19.3519 in assembly 21 | pNIM6 |
| 270 | orf19.5871 | *SNF5* |  | 2091 |  | pNIM6 |
| 271 | orf19.5953 | *SFP1* |  | 1338 |  | pNIM6 |
| 272 | orf19.5975 | *TRY4* |  | 861 |  | pNIM6 |
| 273 | orf19.6102 | *RCA1* |  | 852 |  | pNIM6 |
| 274 | orf19.6239 | *KIN28* |  | 1032 |  | pNIM6 |
| 275 | orf19.6694 |  |  | 1980 |  | pNIM6 |
| 276 | orf19.6719 | *HIR3* |  | 4796 | N-terminal extension with intron in assembly 21 | pNIM6 |
| 277 | orf19.6781 | *ZFU2* |  | 1104 | N-terminal extension with intron in assembly 21 | pNIM6 |
| 278 | orf19.6820 | *TAF12* |  | 1548 |  | pNIM6 |
| 279 | orf19.6845 |  |  | 1440 |  | pNIM6 |
| 280 | orf19.6850 | *RMD5* |  | 1734 |  | pNIM6 |
| 281 | orf19.6874 | *BAS1* |  | 1824 |  | pNIM6 |
| 282 | orf19.6923 | *TAF11* |  | 1032 |  | pNIM6 |
| 283 | orf19.1007 | *KCS1* |  | 1059 |  | pNIM6 |
| 284 | orf19.1111 | *TAF9* |  | 681 |  | pNIM6 |
| 285 | orf19.1178 |  |  | 606 |  | pNIM6 |
| 286 | orf19.1228 | *HAP2* |  | 1095 |  | pNIM6 |
| 287 | orf19.1496 |  |  | 1356 |  | pNIM6 |
| 288 | orf19.1451 | *SRB9* |  | 4998 |  | pNIM6 |
| 289 | orf19.1476 | *IME4* |  | 1632 |  | pNIM6 |
| 290 | orf19.1526 | *SNF2* |  | 5079 |  | pNIM6 |
| 291 | orf19.1528 | *MTF1* |  | 1056 |  | pNIM6 |
| 292 | orf19.1565 | *DUS3* |  | 1842 |  | pNIM6 |
| 293 | orf19.1574 | *TAF7* |  | 1770 |  | pNIM6 |
| 294 | orf19.1577 |  |  | 1329 |  | pNIM6 |
| 295 | orf19.1729 |  |  | 1008 |  | pNIM6 |
| 296 | orf19.1757 | *MET32* |  | 1755 |  | pNIM6 |
| 297 | orf19.1826 | *MDM34* |  | 1869 |  | pNIM6 |
| 298 | orf19.1864 |  |  | 2160 |  | pNIM6 |
| 299 | orf19.2064 |  |  | 3324 |  | pNIM6 |
| 300 | orf19.2099 | *HIR1* |  | 2562 | N-terminal extension with intron in assembly 21 | pNIM6 |
| 301 | orf19.2111 | *TFG2* |  | 1146 |  | pNIM6 |
| 302 | orf19.2135 | *TSM1* |  | 4278 |  | pNIM6 |
| 303 | orf19.2236 | *FHL1* | 2236 | 3459 |  | pNIM6 |
| 304 | orf19.2260 |  |  | 1089 |  | pNIM6 |
| 305 | orf19.2272 | *AFT2* | 2272 | 2385 |  | pNIM6 |
| 306 | orf19.2393 |  |  | 705 | N-terminal extension with 2 introns in assembly 21 | pNIM6 |
| 307 | orf19.2399 | *JJJ1* |  | 1746 |  | pNIM6 |
| 308 | orf19.2458 | *SIP5* |  | 1542 |  | pNIM6 |
| 309 | orf19.653 |  |  | 327 |  | pNIM6 |
| 310 | orf19.1129 |  |  | 588 | merged with orf19.5871 in assembly 21 | pNIM6 |
| 311 | orf19.1694 |  |  | 1581 | merged with orf19.1693 in assembly 21 | pNIM6 |
| 312 | orf19.4125 | *PZF1* |  | 1239 |  | pNIM6 |
| 313 | orf19.6921 |  |  | 1821 | N-terminal extension in assembly 21 | pNIM6 |
| 314 | orf19.7098 |  |  | 645 |  | pNIM6 |
| 315 | orf19.4433 | *CPH1* |  | 1971 |  | pNIM6 |
| 316 | orf19.7454 | *TAF60* |  | 1560 |  | pNIM6 |
| 317 | orf19.1751 | *SPT23* |  | 2232 |  | pNIM6 |
| 318 | orf19.4884 | *WOR1* |  | 2358 |  | pNIM6 |
| 319 | orf19.391 | *UPC2* | *UPC2*^G648D^ | 2139 |  | pNIM6 |
| 320 | orf19.7372 | *MRR1* | *MRR1*^P683S^ | 3327 |  | pNIM1 |
| 321 | orf19.7539 | *INO2* |  | 1140 |  | pNIM6 |
| 322 | orf19.837.1 | *INO4* |  | 477 |  | pNIM6 |
| 323 | orf19.1623 | *CAP1* | *CAP1*^ΔC333^ | 1002 |  | pNIM6 |
| 324 | orf19.1604 | *RHA1* |  | 3205 |  | pNIM6 |
| 325 | orf19.4056 | *BRG1* | *GAT2*^1272^ | 1272 |  | pNIM6 |
| 326 | orf19.801 | *TBF1* |  | 2661 |  | pNIM6 |
| 327 | orf19.3188 | *TAC1* | *TAC1*^G980E^ | 2946 |  | pNIM6 |
| 328 | orf19.6121 | *MNL1* |  | 2718 |  | pNIM6 |
| 329 | orf19.4288 | *CTA7* |  | 2505 |  | pNIM6 |
| 330 | orf19.6203 | *PUT3* |  | 2937 |  | pNIM6 |
| 331 | orf19.6038 | *UGA32* |  | 1752 |  | pNIM6 |
| 332 | orf19.4046 |  |  | 462 |  | pNIM6 |
| 333 | orf19.166 | *ASG1* |  | 2973 |  | pNIM6 |
| 334 | orf19.6888 | *ZFU3* |  | 1439 |  | pNIM6 |
| 335 | orf19.1084 | *CDC39* |  | 6051 |  | pNIM6 |
| 336 | orf19.2545 | *DOT6* |  | 1803 |  | pNIM6 |
| 337 | orf19.2847 | *RPC82* |  | 1788 |  | pNIM6 |
| 338 | orf19.3294 | *MBF1* |  | 456 |  | pNIM6 |
| 339 | orf19.3865 | *RFX1* |  | 2556 |  | pNIM6 |
| 340 | orf19.7017 | *YOX1* |  | 1008 |  | pNIM6 |
| 341 | orf19.7436 | *AAF1* |  | 1839 |  | pNIM6 |
| 342 | orf19.5031 | *SSK1* |  | 2025 |  | pNIM6 |
| 343 | orf19.2884 | *CDC68* |  | 3183 |  | pNIM6 |

^a^ Except for orf19.8243 (clone no. 51, *EFG1*), the orf19 numbers correspond to the systematic name in CGD.

^b^ Unless indicated otherwise, the names of the genes in our library are identical to the standard names or the orf19 number of the genes in CGD. When alleles with different lengths were cloned (for *HAP3* and *BRG1*), the ORF length is given in superscript. *RIM101*^ΔC462^, *STP2*^ΔN100^, *STP1*^ΔN61^, *UPC2*^G648D^, *MRR1*^P683S^, *CAP1*^ΔC433^, and *TAC1*^G980E^ are hyperactive alleles of these genes that have been described in the literature. The K652N mutation in Cph1 (clone no. 24) was introduced by an error in the primer used for amplification of the gene. The correct *CPH1* was also obtained at a later stage (clone no. 315) and had the same phenotypic effect as the *CPH1*^K652N^ allele when it was expressed from the Tet promoter. The R598H mutation in Tcc1 (clone 147) is most likely caused by a PCR error, and a correct *TCC1* clone is missing in the library.

^c^ Alterations that were made in the annotation of the genes in CGD at a later stage. The cloned genes are based on previous annotations in these cases.

All the CGD names are based on *Candida albicans* SC5314 assembly 21

**Table S2. Polymorphisms in cloned Tet-inducible transcription factors**

| **No.** | **orf19 no.** | **Gene name** | **Length (bp)** | **SNPs** | | | | | | | | | | **Indels** |
| --- | --- | --- | --- | --- | --- | --- | --- | --- | --- | --- | --- | --- | --- | --- |
| 1 | orf19.6124 | *ACE2* | 2352 | A1213T | T1281C |  |  |  |  |  |  |  |  |  |
|  |  |  |  | **I405F** |  |  |  |  |  |  |  |  |  |  |
| 2 | orf19.2331 | *ADA2* | 1338 |  |  |  |  |  |  |  |  |  |  |  |
| 3 | orf19.2752 | *ADR1* | 4260 | G156A | A402G | A514G |  |  |  |  |  |  |  | ins273ACA |
|  |  |  |  |  |  | **T172A** |  |  |  |  |  |  |  | **ins91T** |
| 4 | orf19.4766 | *ARG81* | 3300 | A275C |  |  |  |  |  |  |  |  |  | AAT355-357Δ |
|  |  |  |  | **Q92P** |  |  |  |  |  |  |  |  |  | **N119Δ** |
| 5 | orf19.2748 | *ARG83* | 2925 |  |  |  |  |  |  |  |  |  |  |  |
| 6 | orf19.3012 | *ARO80* | 3198 | A219G | G301A | G312A | A534G | G946A | A1389G | C1458T |  |  |  |  |
|  |  |  |  |  | **A101T** |  |  | **G316S** |  |  |  |  |  |  |
| 7 | orf19.3027 | *ARP7* | 1287 |  |  |  |  |  |  |  |  |  |  |  |
| 8 | orf19.2507 | *ARP9* | 1455 | T885C |  |  |  |  |  |  |  |  |  |  |
| 9 | orf19.5343 | *ASH1* | 1350 |  |  |  |  |  |  |  |  |  |  |  |
| 10 | orf19.3964 | *ASH2* | 1401 |  |  |  |  |  |  |  |  |  |  |  |
| 11 | orf19.4043 | *ASK10* | 2259 | G246A | A375G | G549A | T606C | G831A | C1041T | C1077G | C1111T | C1656T | T1686C |  |
|  |  |  |  |  |  |  |  |  |  | **D359E** | **H371Y** |  |  |  |
|  |  |  |  | C2214T | T2226A |  |  |  |  |  |  |  |  |  |
| 12 | orf19.173 | *AZF1* | 3303 | A48T | A51T | A60G | A310G | A652T | C680G | C684T | A703G | A717G | C795T | CAA67-69Δ |
|  |  |  |  | **Q17H** | **Q18H** |  | **T104A** | **T218S** | **T227S** |  | **S235G** |  |  | **Q23Δ** |
|  |  |  |  | A804G | T825C | C837T | A873G | A930G | A1049C | T1275C | C1518T | C1532T | C1550A | ins1539CAG |
|  |  |  |  |  |  |  |  |  | **N350T** |  |  | **S511L** | **P517Q** | **ins513Q** |
|  |  |  |  | T1629A | A1836G |  |  |  |  |  |  |  |  | CAA1888-1890Δ |
|  |  |  |  |  |  |  |  |  |  |  |  |  |  | **Q630Δ** |
|  |  |  |  |  |  |  |  |  |  |  |  |  |  | ins2127CAGCAACAGCAA |
|  |  |  |  |  |  |  |  |  |  |  |  |  |  | **ins709QQQQ** |
| 13 | orf19.3809 | *BAS1* | 2217 | C183T | G247A | G248A | G761A | G768A | A861C | G987A | C1047G | A1224G | A1275G |  |
|  |  |  |  |  | **G83N** | **G83N** | **G254E** |  | **Q287H** |  |  |  |  |  |
|  |  |  |  | A1498G |  |  |  |  |  |  |  |  |  |  |
|  |  |  |  | **T500A** |  |  |  |  |  |  |  |  |  |  |

| 14 | orf19.723 | *BCR1* | 2202 | T1455C | A1753G | T1889C | A1946T | T2049A |  |  |  |  |  | AATAATAAT1774-1782Δ |
| --- | --- | --- | --- | --- | --- | --- | --- | --- | --- | --- | --- | --- | --- | --- |
|  |  |  |  |  | **N585D** | **L630S** | **N649I** |  |  |  |  |  |  | **NNN592-594Δ** |
|  |  |  |  |  |  |  |  |  |  |  |  |  |  | ins2028AAT |
|  |  |  |  |  |  |  |  |  |  |  |  |  |  | **ins676N** |
|  |  |  |  |  |  |  |  |  |  |  |  |  |  | ACAACAAATACAAAT2125-2139Δ |
|  |  |  |  |  |  |  |  |  |  |  |  |  |  | **TTNTN709-713Δ** |
| 15 | orf19.978 | *BDF1* | 2130 |  |  |  |  |  |  |  |  |  |  |  |
| 16 | orf19.976 | *BRE1* | 2046 |  |  |  |  |  |  |  |  |  |  |  |
| 17 | orf19.6649 | *BRF1* | 1662 | C1314T |  |  |  |  |  |  |  |  |  |  |
| 18 | orf19.1623 | *CAP1* | 1500 | C972T | A1212G | A1362G |  |  |  |  |  |  |  |  |
| 19 | orf19.1135 | *CAS1* | 2436 | T136C | T138G | T165A | C222T |  |  |  |  |  |  |  |
|  |  |  |  | **S46P** |  | **F55L** |  |  |  |  |  |  |  |  |
| 20 | orf19.4670 | *CAS5* | 2466 | C168T | T2346C |  |  |  |  |  |  |  |  |  |
| 21 | orf19.5097 | *CAT8* | 3171 |  |  |  |  |  |  |  |  |  |  |  |
| 22 | orf19.2876 | *CBF1* | 672 |  |  |  |  |  |  |  |  |  |  |  |
| 23 | orf19.4542 | *CCL1* | 1188 | A378T |  |  |  |  |  |  |  |  |  |  |
| 24 | orf19.4433 | *CPH1*^K652N^ | 1965 | G1122A | A1164G | G1353A | A1528G | T1880A | A1956C |  |  |  |  | CAACAG1732-1737Δ |
|  |  |  |  |  |  |  | **T510A** | **V627D** | **K652N** |  |  |  |  | **QQ578-579Δ** |
| 25 | orf19.1187 | *CPH2* | 2562 | C147T | T396C | A693T |  |  |  |  |  |  |  |  |
| 26 | orf19.7359 | *CRZ1* | 2196 |  |  |  |  |  |  |  |  |  |  |  |
| 27 | orf19.2356 | *CRZ2* | 1554 | A63T | A76G | C84T | G168C | A172G | C191T | A193G | T258C | A447G | G450A | ins465CAACAACAA |
|  |  |  |  |  | **T26A** |  |  | **T58A** | **T64I** | **S65G** |  |  |  | **ins155QQQ** |
|  |  |  |  | G474A | T480C | G535A | T612C | G831A | C963A | T984C | T1000G |  |  |  |
|  |  |  |  |  |  | **A179T** |  |  |  |  | **S334A** |  |  |  |
| 28 | orf19.3794 | *CSR1* | 2124 | G27A | C315T | T807C | C1701T | G1956A | A1971G | G2007A | C2046T | C2115T |  | GGCAGCATGAGCAACAGA1064-1081Δ |
|  |  |  |  | **V9I** |  |  |  |  |  |  |  |  |  | **G355E, QHEQQK356-361Δ** |
| 29 | orf19.5467 | *TLO7* | 510 |  |  |  |  |  |  |  |  |  |  |  |
| 30 | orf19.4647 | *HAP3* | 1109 |  |  |  |  |  |  |  |  |  |  |  |
| 31 | orf19.632 | *CTA2* | 1212 | C773T | C1056A |  |  |  |  |  |  |  |  |  |
|  |  |  |  | **S258F** | **H352Q** |  |  |  |  |  |  |  |  |  |
| 32 | orf19.1032 | *SKO1* | 1841 | G650T | G794A | T871A | G881A | G890A | A894G | T939C | C963A | A1727G |  | CCACAA570-575Δ |
|  |  |  |  |  |  | **L254Q** |  |  | **T262A** | **S277P** | **H285N** |  |  | **PQ154-155Δ** |

| 33 | orf19.1166 | *CTA3* | 3993 | T1425C | C1449T | G1462T | G1482A | C1563T | G1779A | C1795A | A1802G | A1842G | C2186T |  |
| --- | --- | --- | --- | --- | --- | --- | --- | --- | --- | --- | --- | --- | --- | --- |
|  |  |  |  |  |  | **A482S** |  |  |  | **L599I** | **K601R** |  | **T729I** |  |
|  |  |  |  | T2595A | C2607T | A3245G | G3596A | C3742A |  |  |  |  |  |  |
|  |  |  |  | **N865K** |  | **N1082S** | **G1199E** | **S1248M** |  |  |  |  |  |  |
| 34 | orf19.3102 | *CTA6* | 642 |  |  |  |  |  |  |  |  |  |  |  |
| 35 | orf19.3315 | *CTA9* | 1284 | C753A | G764C | C777T |  |  |  |  |  |  |  |  |
|  |  |  |  |  | **G265A** |  |  |  |  |  |  |  |  |  |
| 36 | orf19.7127 | *TLO16* | 528 |  |  |  |  |  |  |  |  |  |  |  |
| 37 | orf19.3401 | *CTA1* | 657 | T69C | A108G |  |  |  |  |  |  |  |  |  |
| 38 | orf19.1925 | *TLO5* | 531 |  |  |  |  |  |  |  |  |  |  |  |
| 39 | orf19.4054 | *CTA24* | 759 | C181A |  |  |  |  |  |  |  |  |  |  |
|  |  |  |  | **H61N** |  |  |  |  |  |  |  |  |  |  |
| 40 | orf19.4590 | *RFX2* | 3336 | G2135A |  |  |  |  |  |  |  |  |  |  |
|  |  |  |  | R712Q |  |  |  |  |  |  |  |  |  |  |
| 41 | orf19.7680 | *CTA26* | 822 |  |  |  |  |  |  |  |  |  |  |  |
| 42 | orf19.6337 | *TLO13* | 945 |  |  |  |  |  |  |  |  |  |  |  |
| 43 | orf19.7374 | *CTA4* | 3051 |  |  |  |  |  |  |  |  |  |  |  |
| 44 | orf19.4775 | *CTA8* | 2280 | T217C | A224T | G490A | C816T | T1443C | G1454A | A1701C | A1734T | G2064T | G2119A | CGA697-699Δ |
|  |  |  |  | **Y73H** | **Q75L** | **E164K** |  |  | **S485N** |  |  | **E688D** | **G707S** | **R233Δ** |
|  |  |  |  | C2142T | T2151C | A2165G | A2174G | A2187G | A2230G | G2253A | A2259G |  |  | AGT1636-1638Δ |
|  |  |  |  |  |  | **N722S** | **D725G** |  | **N744D** |  |  |  |  | **S546Δ** |
| 45 | orf19.1499 | *CTF1* | 3420 | A500C | G1294A | A1480G | T1728C |  |  |  |  |  |  | AACAACAACAACAAC673-687Δ |
|  |  |  |  | **E167A** | **A432T** | **I494V** |  |  |  |  |  |  |  | **NNNNN225-229Δ** |
| 46 | orf19.5001 | *CUP2* | 1776 |  |  |  |  |  |  |  |  |  |  |  |
| 47 | orf19.6514 | *CUP9* | 1035 |  |  |  |  |  |  |  |  |  |  |  |
| 48 | orf19.5849 | *CWT1* | 1737 |  |  |  |  |  |  |  |  |  |  |  |
| 49 | orf19.3127 | *CZF1* | 1158 |  |  |  |  |  |  |  |  |  |  |  |
| 50 | orf19.3252 | *DAL81* | 2637 |  |  |  |  |  |  |  |  |  |  |  |
| 51 | orf19.8243 | *EFG1* | 1650 |  |  |  |  |  |  |  |  |  |  |  |
| 52 | orf19.5498 | *EFH1* | 2163 |  |  |  |  |  |  |  |  |  |  |  |
| 53 | orf19.3722 | *FAP1* | 2550 |  |  |  |  |  |  |  |  |  |  |  |
| 54 | orf19.6817 | *FCR1* | 1554 |  |  |  |  |  |  |  |  |  |  |  |
| 55 | orf19.2054 | *FGR15* | 1557 |  |  |  |  |  |  |  |  |  |  |  |
| 56 | orf19.5729 | *FGR17* | 1965 | G84C | G450A | A738T | G915A | C1639G | G1671A | A1902G | T1932C |  |  |  |
|  |  |  |  |  |  |  |  | **P547A** |  |  |  |  |  |  |
| 57 | orf19.6680 | *FGR27* | 2364 |  |  |  |  |  |  |  |  |  |  |  |
| 58 | orf19.5389 | *FKH2* | 1581 |  |  |  |  |  |  |  |  |  |  |  |
| 59 | orf19.1093 | *FLO8* | 2448 | A969G | G1009A | C1105T | T1152C | C1736A | T1863G | A2040G | G2043A | T2406C |  | CAG871-873Δ |
|  |  |  |  |  | **A337T** | **P369S** |  | **P579Q** |  |  |  |  |  | **Q291Δ** |
|  |  |  |  |  |  |  |  |  |  |  |  |  |  | CAA1177-1179Δ |
|  |  |  |  |  |  |  |  |  |  |  |  |  |  | **Q393Δ** |
| 60 | orf19.5338 | *GAL4* | 786 |  |  |  |  |  |  |  |  |  |  |  |
| 61 | orf19.1275 | *GAT1* | 2067 |  |  |  |  |  |  |  |  |  |  |  |
| 62 | orf19.4056 | *BRG1* | 1341 | A125G | A283T |  |  |  |  |  |  |  |  |  |
|  |  |  |  | **N42S** | **T95S** |  |  |  |  |  |  |  |  |  |
| 63 | orf19.1358 | *GCN4* | 969 | G215T |  |  |  |  |  |  |  |  |  | GCT439-441Δ |
|  |  |  |  | **G72V** |  |  |  |  |  |  |  |  |  | **A147Δ** |
| 64 | orf19.3182 | *GIS2* | 528 | A456T | T477A |  |  |  |  |  |  |  |  |  |
| 65 | orf19.3912 | *GLN3* | 2049 |  |  |  |  |  |  |  |  |  |  |  |
| 66 | orf19.1150 | *GLN32* | 1419 | C93T | G276A |  |  |  |  |  |  |  |  |  |
| 67 | orf19.720 | *GST3* | 648 | C234T | G535C | G622A |  |  |  |  |  |  |  |  |
|  |  |  |  |  | **V179L** | **V208I** |  |  |  |  |  |  |  |  |
| 68 | orf19.6393 | *GTS1* | 1251 |  |  |  |  |  |  |  |  |  |  |  |
| 69 | orf19.2842 | *GZF3* | 2133 |  |  |  |  |  |  |  |  |  |  |  |
| 70 | orf19.2432 | *HAC1* | 1074 |  |  |  |  |  |  |  |  |  |  |  |
| 71 | orf19.3190 | *HAL9* | 3033 |  |  |  |  |  |  |  |  |  |  |  |
| 72 | orforf19.4647 | *HAP3*^882^ | 882 |  |  |  |  |  |  |  |  |  |  |  |
| 73 | orf19.517 | *HAP31* | 318 |  |  |  |  |  |  |  |  |  |  |  |
| 74 | orf19.740 | *HAP41* | 1146 |  |  |  |  |  |  |  |  |  |  |  |
| 75 | orf19.1481 | *HAP42* | 2517 | C671T | T1359C | C1554T | A1578G | C1724T | T1730C | T1905C | T2064C | A2085G | C2184A |  |
|  |  |  |  | **T224I** |  |  |  | **A575V** | **I577T** |  |  |  |  |  |
|  |  |  |  | A2200G | A2207G | A2411G |  |  |  |  |  |  |  |  |
|  |  |  |  | **N734D** | **D736G** | **Q804R** |  |  |  |  |  |  |  |  |
| 76 | orf19.681 | *HAP43* | 1905 |  |  |  |  |  |  |  |  |  |  |  |
| 77 | orf19.1973 | *HAP5* | 1047 | G49A | G57A | T152A | A567T |  |  |  |  |  |  |  |
|  |  |  |  | **A17T** |  | **V51E** |  |  |  |  |  |  |  |  |
| 78 | orf19.4853 | *HCM1* | 1740 |  |  |  |  |  |  |  |  |  |  |  |
| 79 | orf19.1715 | *IRO1* | 1791 |  |  |  |  |  |  |  |  |  |  |  |
| 80 | orf19.3736 | *KAR4* | 1110 |  |  |  |  |  |  |  |  |  |  |  |

| 81 | orf19.4225 | *LEU3* | 2955 | G118C | A132G | C135T | T140C | A141G | G261A | A293G | C348T | A381G | T430A |  |
| --- | --- | --- | --- | --- | --- | --- | --- | --- | --- | --- | --- | --- | --- | --- |
|  |  |  |  | **A40P** |  |  | **I47T** | **I47T** |  | **Q98R** |  | **I127M** | **S144T** |  |
|  |  |  |  | A468G | T490A | C522T | G532C | T903C | A969G | A978C | A1059G | T1434C | C1603T |  |
|  |  |  |  |  | **Y164N** |  | **A178P** |  |  |  |  |  |  |  |
|  |  |  |  | G1704A | T1734C | A1814G | C1821G | A1827G | G2688A | T2713C | T2714A | T2832C | G2844A |  |
|  |  |  |  |  |  | **N605S** |  |  |  | **L905Q** | **L905Q** |  |  |  |
|  |  |  |  | G2868A |  |  |  |  |  |  |  |  |  |  |
| 82 | orf19.3505 | *SLM2* | 2397 |  |  |  |  |  |  |  |  |  |  |  |
| 83 | orf19.5548 | *LYS14* | 1779 | C1698T |  |  |  |  |  |  |  |  |  |  |
| 84 | orf19.4778 | *LYS142* | 2070 | C120T | C819G | T933A |  |  |  |  |  |  |  |  |
| 85 | orf19.4776 | *LYS143* | 2631 | G1779C | C1942T | T2016C | T2043C | T2108C | C2111T | G2251C | A2346G |  |  |  |
|  |  |  |  |  |  |  |  | **V703A** | **T704I** | **G751R** |  |  |  |  |
| 86 | orf19.5380 | *LYS144* | 2586 | G779A | T1218C | C1333T | C1854T | A2407G |  |  |  |  |  |  |
|  |  |  |  | **G260E** |  |  |  | **S803G** |  |  |  |  |  |  |
| 87 | orf19.7068 | *MAC1* | 1296 |  |  |  |  |  |  |  |  |  |  |  |
| 88 | orf19.5855 | *MBP1* | 2559 | A1218T | T1269C | G1332A | T1803C | C1922A |  |  |  |  |  |  |
|  |  |  |  |  |  |  |  | **T641K** |  |  |  |  |  |  |
| 89 | orf19.7025 | *MCM1* | 789 |  |  |  |  |  |  |  |  |  |  |  |
| 90 | orf19.1706 | *MET18* | 3264 | T28C | T57C | T186G | C308T | A903G | G918A | G1101A | G1890A | C1902T | G1924A | ACA94-96Δ |
|  |  |  |  | **L10S** |  |  | **S103L** |  |  |  |  |  | **V642I** | **T32Δ** |
|  |  |  |  | T2333C | A2540G | C2663A | C2676T | G2738T | T2810A | T2887G |  |  |  | GAT2359-2361Δ |
|  |  |  |  | **I778T** | **R847L** | **A888D** |  | **R913L** | **I937K** | **Y963D** |  |  |  | **D787Δ** |
| 91 | orf19.7046 | *MET28* | 522 |  |  |  |  |  |  |  |  |  |  |  |
| 92 | orf19.5312 | *MET4* | 1158 |  |  |  |  |  |  |  |  |  |  |  |
| 93 | orf19.4318 | *MIG1* | 1725 | T174C | A186G | G339A | A558C | T699C | T781C | T1446C | T1468C | A1620G |  | ins543CATCAGCAACAG |
|  |  |  |  |  |  |  | **G186H** |  | **S261P** |  | **F490L** |  |  | **ins181HQQQ** |
|  |  |  |  |  |  |  |  |  |  |  |  |  |  | TAC553-555Δ |
|  |  |  |  |  |  |  |  |  |  |  |  |  |  | **Y185Δ** |
|  |  |  |  |  |  |  |  |  |  |  |  |  |  | ins798CAA |
|  |  |  |  |  |  |  |  |  |  |  |  |  |  | **ins266Q** |
| 94 | orf19.5326 | *MIG2* | 810 |  |  |  |  |  |  |  |  |  |  |  |
| 95 | orf19.4752 | *MSN4* | 2274 | T90C | T128C | G169A | G609A | A1332G | A1384C | C1491T |  |  |  | CCA349-351Δ |
|  |  |  |  |  | **L43S** | **D57N** |  |  | **N462H** |  |  |  |  | **P117Δ** |

| 96 | orf19.6309 | *MSS11* | 1908 | G863A | C972T | G994A | A1292C | A1293C | A1296G | T1486C | G1488A |  |  | CAA1288-1290Δ |
| --- | --- | --- | --- | --- | --- | --- | --- | --- | --- | --- | --- | --- | --- | --- |
|  |  |  |  | **R288K** |  | **E332K** | **Q431P** | **Q431P** |  | **S496P** |  |  |  | **Q430Δ** |
|  |  |  |  |  |  |  |  |  |  |  |  |  |  | CAA1309-1311Δ |
|  |  |  |  |  |  |  |  |  |  |  |  |  |  | **Q437Δ** |
| 97 | orf19.3201 | *MTLA1* | 963 | C766T |  |  |  |  |  |  |  |  |  |  |
| 98 | orf19.3200 | *MTLA2* | 661 |  |  |  |  |  |  |  |  |  |  |  |
| 99 | orf19.10712 | *MTLALPHA1* | 582 |  |  |  |  |  |  |  |  |  |  |  |
| 100 | orf19.10708 | *MTLALPHA2* | 620 |  |  |  |  |  |  |  |  |  |  |  |
| 101 | orf19.5825 | *NCB2* | 450 |  |  |  |  |  |  |  |  |  |  |  |
| 102 | orf19.339 | *NDE1* | 1725 | C432T |  |  |  |  |  |  |  |  |  |  |
| 103 | orf19.2119 | *NDT80* | 1503 | C72T | A246G |  |  |  |  |  |  |  |  | CAGCAGCAACAG226-237Δ |
|  |  |  |  |  |  |  |  |  |  |  |  |  |  | **QQQQ76-79Δ** |
| 104 | orf19.513 | *RON1* | 1473 | T906A |  |  |  |  |  |  |  |  |  |  |
| 105 | orf19.3023 | *NGG1* | 1884 | G122A | G1077A |  |  |  |  |  |  |  |  |  |
|  |  |  |  | **S41N** |  |  |  |  |  |  |  |  |  |  |
| 106 | orf19.2379 | *NOT4* | 1668 | A1635G |  |  |  |  |  |  |  |  |  | ins1320CAACAA |
|  |  |  |  |  |  |  |  |  |  |  |  |  |  | **ins440QQ** |
| 107 | orf19.7150 | *NRG1* | 933 |  |  |  |  |  |  |  |  |  |  |  |
| 108 | orf19.5268 | *MED10* | 513 |  |  |  |  |  |  |  |  |  |  |  |
| 109 | orf19.1543 | *OPI1* | 1293 |  |  |  |  |  |  |  |  |  |  | CAACAACTG1033-1041Δ |
|  |  |  |  |  |  |  |  |  |  |  |  |  |  | **QQS345-347Δ** |
| 110 | orf19.4863 | *PDC2* | 2511 | A24G | G1173A | C1303A |  |  |  |  |  |  |  |  |
|  |  |  |  |  |  | **P435T** |  |  |  |  |  |  |  |  |
| 111 | orf19.4000 | *GRF10* | 2058 | T1486A |  |  |  |  |  |  |  |  |  |  |
|  |  |  |  | **Y496N** |  |  |  |  |  |  |  |  |  |  |
| 112 | orf19.1253 | *PHO4* | 1980 | A90G | A149G | G429A | T437A | A441G | C476G |  |  |  |  |  |
|  |  |  |  |  | **Q50R** |  | **L146Q** |  | **S159C** |  |  |  |  |  |
| 113 | orf19.3986 | *PPR1* | 2595 | T2268C | A2329G | G2358A | C2457T |  |  |  |  |  |  | ins2283AATAATAATAAT |
|  |  |  |  |  | **S777G** |  |  |  |  |  |  |  |  | **ins761NNNN** |
| 114 | orf19.7119 | *RAD3* | 2298 |  |  |  |  |  |  |  |  |  |  |  |
| 115 | orf19.1773 | *RAP1* | 1293 | C81T | A171G | A190T | A192T | G233A | T1011A |  |  |  |  | ins681CCT |
|  |  |  |  |  |  | **T64S** | **T64S** | **R78Q** |  |  |  |  |  | **ins227P** |

| 116 | orf19.5558 | *RBF1* | 1584 | A228T | T231A | A234T | T258A | A273G | G282A | A285G | G948A | G1071A | T1182G | GCACAGGCTCAA355-366Δ |
| --- | --- | --- | --- | --- | --- | --- | --- | --- | --- | --- | --- | --- | --- | --- |
|  |  |  |  |  |  |  |  |  |  |  |  |  |  | **AQAQ119-122Δ** |
|  |  |  |  | C1306G |  |  |  |  |  |  |  |  |  | CAGCAACAA1060-1068Δ |
|  |  |  |  | **P436A** |  |  |  |  |  |  |  |  |  | **QQQ354-356Δ** |
| 117 | orf19.2230 | *RDS3* | 360 |  |  |  |  |  |  |  |  |  |  |  |
| 118 | orf19.2823 | *RFG1* | 1806 | T1043A | T1071A | T1569G | T1776A |  |  |  |  |  |  | ins1773CAA |
|  |  |  |  | **L348Q** | **H357Q** |  | **H592Q** |  |  |  |  |  |  | **ins591Q** |
| 119 | orf19.2747 | *RGT1* | 3078 | T1407A | G1437T | G1767A |  |  |  |  |  |  |  | CAA1372-1374Δ |
|  |  |  |  |  | **M479I** |  |  |  |  |  |  |  |  | **Q458Δ** |
|  |  |  |  |  |  |  |  |  |  |  |  |  |  | CAACTACAA1384-1392Δ |
|  |  |  |  |  |  |  |  |  |  |  |  |  |  | **QAQ462-464Δ** |
| 120 | orf19.7247 | *RIM101*^ΔC462^ | 1389 |  |  |  |  |  |  |  |  |  |  |  |
| 121 | orf19.4662 | *RLM1* | 1836 | G81A | C1235T |  |  |  |  |  |  |  |  |  |
|  |  |  |  |  | **A412V** |  |  |  |  |  |  |  |  |  |
| 122 | orf19.4438 | *RME1* | 1524 | G243A | A1365T |  |  |  |  |  |  |  |  |  |
| 123 | orf19.2743 | *RPH1* | 1821 |  |  |  |  |  |  |  |  |  |  |  |
| 124 | orf19.1069 | *RPN4* | 1611 | G564A |  |  |  |  |  |  |  |  |  |  |
| 125 | orf19.519 | *RRN10* | 588 |  |  |  |  |  |  |  |  |  |  |  |
| 126 | orf19.718 | *RRN11* | 2070 | T385C | G428A | T494C | A1310G |  |  |  |  |  |  | GATTTT2043-2058Δ |
|  |  |  |  |  | **S143N** | **L165R** | **K437R** |  |  |  |  |  |  | **DF685-686Δ** |
| 127 | orf19.1923 | *RRN3* | 1671 | C1554T |  |  |  |  |  |  |  |  |  |  |
| 128 | orf19.4420 | *RRN6* | 2403 | A768G | C802T | G955A | G971T | G988A | C1694T | C1722T | G1831A | A1886G | G2020A |  |
|  |  |  |  |  | **P268S** | **V319I** | **R324L** | **V330I** | **S565F** |  | **V611M** | **K629R** | **D674N** |  |
|  |  |  |  | C2029A | G2110A | G2181T | G2193A | G2351A |  |  |  |  |  |  |
|  |  |  |  | **Q677K** | **D704N** | **K727N** |  | **R784K** |  |  |  |  |  |  |
| 129 | orf19.1589 | *RRN7* | 1314 |  |  |  |  |  |  |  |  |  |  |  |
| 130 | orf19.4722 | *RTG1* | 825 |  |  |  |  |  |  |  |  |  |  |  |
| 131 | orf19.2315 | *RTG3* | 1563 | T57C | C147T | T795C | C983T | G1375A |  |  |  |  |  |  |
|  |  |  |  |  |  |  | **T328I** | **D459N** |  |  |  |  |  |  |
| 132 | orf19.3753 | *SEF1* | 2754 | C2646T |  |  |  |  |  |  |  |  |  |  |
| 133 | orf19.1926 | *SEF2* | 1986 | C1185T | C1299T | G1351T | T1719G |  |  |  |  |  |  |  |
|  |  |  |  |  |  | **V451L** |  |  |  |  |  |  |  |  |
| 134 | orf19.1755 | *SET2* | 2535 |  |  |  |  |  |  |  |  |  |  |  |
| 135 | orf19.3665 | *SET6* | 1140 | T291A | C415G | T558C | C750T | C798T | C870T | C873T |  |  |  |  |
|  |  |  |  |  | **P139A** |  |  |  |  |  |  |  |  |  |
| 136 | orf19.454 | *SFL1* | 2418 |  |  |  |  |  |  |  |  |  |  |  |
| 137 | orf19.4869 | *SFU1* | 1554 | C806A |  |  |  |  |  |  |  |  |  |  |
|  |  |  |  | **T269N** |  |  |  |  |  |  |  |  |  |  |
| 138 | orf19.6011 | *SIN3* | 4639 |  |  |  |  |  |  |  |  |  |  |  |
| 139 | orf19.971 | *SKN7* | 1680 |  |  |  |  |  |  |  |  |  |  |  |
| 140 | orf19.487 | *SPT14* | 1359 |  |  |  |  |  |  |  |  |  |  |  |
| 141 | orf19.422 | *SPT20* | 2244 | A156G | G924A | A1062T | A1458C | T1563C | G1666A | C1667G | A2055G | A2067G | A2076G | CAACAGCAACAA2053-2064Δ |
|  |  |  |  |  |  |  |  |  | **A556S** | **A556S** |  |  |  | **QQQQ685-688Δ** |
|  |  |  |  | T2130C |  |  |  |  |  |  |  |  |  |  |
| 142 | orf19.7622 | *SPT3* | 987 |  |  |  |  |  |  |  |  |  |  |  |
| 143 | orf19.7572 | *SPT7* | 3924 |  |  |  |  |  |  |  |  |  |  |  |
| 144 | orf19.4312 | *SPT8* | 2307 | T212A | A357T | A855G | A927T | T1911C |  |  |  |  |  | GAAGAA349-354Δ |
|  |  |  |  | **V71E** | **E119D** |  |  |  |  |  |  |  |  | **EE117-118Δ** |
| 145 | orf19.1457 | *SSL1* | 1419 |  |  |  |  |  |  |  |  |  |  |  |
| 146 | orf19.6798 | *SSN6* | 3243 |  |  |  |  |  |  |  |  |  |  |  |
| 147 | orf19.6734 | *TCC1*^R598H^ | 2211 | G1793A |  |  |  |  |  |  |  |  |  |  |
|  |  |  |  | **R598H** |  |  |  |  |  |  |  |  |  |  |
| 148 | orf19.7355 | *SSN8* | 1305 |  |  |  |  |  |  |  |  |  |  |  |
| 149 | orf19.3308 | *STB5* | 1923 | A47C | T1521C |  |  |  |  |  |  |  |  |  |
|  |  |  |  | **H16P** |  |  |  |  |  |  |  |  |  |  |
| 150 | orf19.6173 | *STD1* | 1008 |  |  |  |  |  |  |  |  |  |  |  |
| 151 | orf19.4961 | *STP2*^ΔN100^ | 1449 | C540T | C570T |  |  |  |  |  |  |  |  | ins514TTAAAC |
|  |  |  |  |  |  |  |  |  |  |  |  |  |  | **E172VKQ** |
|  |  |  |  |  |  |  |  |  |  |  |  |  |  | CATCAACAACATGTA1330-1344Δ |
|  |  |  |  |  |  |  |  |  |  |  |  |  |  | **HQQHV444-448Δ** |
| 152 | orf19.5917 | *STP1*^ΔN61^ | 1131 |  |  |  |  |  |  |  |  |  |  |  |
| 153 | orf19.909 | *STP4* | 1131 | G556A | G654A |  |  |  |  |  |  |  |  |  |
|  |  |  |  | **D186N** |  |  |  |  |  |  |  |  |  |  |
| 154 | orf19.3059 | *SUA71* | 1035 | G618A | T687C |  |  |  |  |  |  |  |  |  |
| 155 | orf19.3519 | *SUA72* | 1041 | C171T | C541A | T552C | T738C | T814C | A819T |  |  |  |  |  |
| 156 | orf19.7319 | *SUC1* | 1506 |  |  |  |  |  |  |  |  |  |  |  |
| 157 | orf19.5657 | *SWI1* | 2964 | T1932C | A2331G |  |  |  |  |  |  |  |  |  |
| 158 | orf19.4488 | *SWI3* | 2934 | C61A | A140G | A242C | T369C | A406G | C407T | C485T | G493T | A509G | A828T | ins78CAACAACAACAACAA |
|  |  |  |  | **P21T** | **N47S** | **E81S** |  | **T136V** | **T136V** | **A162V** | **A165S** | **E170G** |  | **ins26QQQQQ** |
|  |  |  |  | G1114A | A1932T | T1986A | A2208C | G2211A | T2235C | A2285G |  |  |  | ins882GAA |
|  |  |  |  | **D372N** |  | **N662K** |  | **M737I** |  | **N762S** |  |  |  | **ins294E** |
| 159 | orf19.4545 | *SWI4* | 2994 | C2364T |  |  |  |  |  |  |  |  |  | TCACTC2233-2238Δ |
|  |  |  |  |  |  |  |  |  |  |  |  |  |  | **SL745-746Δ** |
| 160 | orf19.4725 | *SWI6* | 2190 | G672A | T673C | C753T | G1146A |  |  |  |  |  |  |  |
|  |  |  |  |  | **F225L** |  |  |  |  |  |  |  |  |  |
| 161 | orf19.3188 | *TAC1* | 2946 | T139A | T140A | T1257C | T1305C | T1743C | A2202G | T2214C | T2316A | G2326A | G2485C |  |
|  |  |  |  | **L47K** | **L47K** |  |  |  |  |  | **N772K** | **D776N** | **E829Q** |  |
| 162 | orf19.6193 | *TAF145* | 3777 | C1257A | A2241T | G2691A | A3171G | C3351T | T3465A |  |  |  |  | CAA3127-3129Δ |
|  |  |  |  | **S419R** |  |  |  |  |  |  |  |  |  | **Q1043Δ** |
|  |  |  |  |  |  |  |  |  |  |  |  |  |  | CAACAA3163-3168Δ |
|  |  |  |  |  |  |  |  |  |  |  |  |  |  | **QQ1055-1056Δ** |
| 163 | orf19.5174 | *TAF19* | 498 | G422A |  |  |  |  |  |  |  |  |  | GAT376-378Δ |
|  |  |  |  | **G141E** |  |  |  |  |  |  |  |  |  | **D126Δ** |
| 164 | orf19.1885 | *TAF4* | 1092 | G79A | C267T |  |  |  |  |  |  |  |  |  |
|  |  |  |  | **E27K** |  |  |  |  |  |  |  |  |  |  |
| 165 | orf19.1693 | *CAS4* | 8493 | C366T | T397C | C403T | T469C | G552A | T1404C | T2811A | C2817A | A2982G | C3084T |  |
|  |  |  |  |  | **S133P** | **P135S** | **S157P** |  |  | **D937E** |  |  |  |  |
|  |  |  |  | T3126C | G3128A | T3306A | C3372T | T3453A | T3837A | T3861C | G4068A | A4107T | T4122A |  |
|  |  |  |  |  | **S1043N** |  |  |  |  |  |  |  |  |  |
|  |  |  |  | T4137C | T4140C | G4146A | A4194G | T4200C | G4203T | T4206C | G4233A | A4341G | C4452G |  |
|  |  |  |  |  |  |  |  |  |  |  |  |  | **I1484M** |  |
|  |  |  |  | A4692G | G5035A | T5091C | A5211G | T5215C | G5475C | T5526C | C5537T | T5601C | G5663A |  |
|  |  |  |  |  | **V1679I** |  | **I1737M** | **S1739P** | **L1825F** |  | **T1846I** |  | **S1888N** |  |
|  |  |  |  | T5781C | G5954A | A6126G | G6189A | G6237A | G6474A | A6519G | T6552C | T6768A | G6789A |  |
|  |  |  |  |  | **R1985K** |  |  |  |  |  |  |  |  |  |
|  |  |  |  | C6886A | C7091T | C7098T | C7734T | T8043C | G8058A | T8127C |  |  |  |  |
|  |  |  |  |  | **S2364L** |  |  |  |  |  |  |  |  |  |
| 166 | orf19.1837 | *TBP1* | 717 |  |  |  |  |  |  |  |  |  |  |  |
| 167 | orf19.6985 | *TEA1* | 2703 |  |  |  |  |  |  |  |  |  |  |  |
| 168 | orf19.5908 | *TEC1* | 2232 |  |  |  |  |  |  |  |  |  |  |  |
| 169 | orf19.4851 | *TFA1* | 1185 |  |  |  |  |  |  |  |  |  |  |  |
| 170 | orf19.4601 | *TFC1* | 1518 | C1146T |  |  |  |  |  |  |  |  |  |  |
| 171 | orf19.3833 | *TFC3* | 4212 | T2037A | A2556G | C2559T | T2583C | G2641A | C2737A | A2740G | G3782A | G3861A | T3873G | ins708GAGAATGATGAA |
|  |  |  |  |  |  |  |  | **A881T** | **P913T** | **I914V** | **R1261K** |  |  | **ins236ENDE** |
|  |  |  |  | C3876A | A3895G |  |  |  |  |  |  |  |  |  |
|  |  |  |  |  | **N1299D** |  |  |  |  |  |  |  |  |  |
| 172 | orf19.2325 | *TFC6* | 1554 |  |  |  |  |  |  |  |  |  |  |  |
| 173 | orf19.4585 | *TFG1* | 1827 | A291G |  |  |  |  |  |  |  |  |  |  |
| 174 | orf19.889 | *THI20* | 1647 | G499A | A703G | C723T |  |  |  |  |  |  |  |  |
|  |  |  |  | **D167N** | **N235D** |  |  |  |  |  |  |  |  |  |
| 175 | orf19.4625 | *TOA2* | 393 |  |  |  |  |  |  |  |  |  |  |  |
| 176 | orf19.668 | *TOS4* | 1479 | C747T | C943G | C1047T | A1060G | G1077A | G1125A | T1347C |  |  |  |  |
|  |  |  |  |  | **L315V** |  | **I354V** |  |  |  |  |  |  |  |
| 177 | orf19.6109 | *TUP1* | 1533 | A111G | T255C | G309A | C362T | T573C | C615T | T938C | C1299T | T1373C |  | CAA337-339Δ |
|  |  |  |  |  |  |  | **A121V** |  |  | **V313A** |  | **V458A** |  | **Q113Δ** |
| 178 | orf19.4941 | *TYE7* | 810 |  |  |  |  |  |  |  |  |  |  |  |
| 179 | orf19.7570 | *UGA3* | 2412 |  |  |  |  |  |  |  |  |  |  |  |
| 180 | orf19.7317 | *UGA33* | 1449 |  |  |  |  |  |  |  |  |  |  |  |
| 181 | orf19.1822 | *UME6* | 2538 | A142T | G782A | A813G | G1199A | T1200C | A1719T |  |  |  |  | ins243CAACAA |
|  |  |  |  | **M48L** | **S261N** | **I271M** | **S400N** | **S400N** | **K537N** |  |  |  |  | **ins81QQ** |
| 182 | orf19.2745 | *UME7* | 1377 | T1040A | C1048A | C1127G |  |  |  |  |  |  |  |  |
|  |  |  |  | **I347K** | **P350T** | **T376R** |  |  |  |  |  |  |  |  |
| 183 | orf19.391 | *UPC2* | 2139 | T1338C | C1392T | C1410A | C1539T |  |  |  |  |  |  |  |
| 184 | orf19.155 | *URE2* | 1035 | C678T | T897C |  |  |  |  |  |  |  |  |  |
| 185 | orf19.1035 | *WAR1* | 2844 | T819G | T1638C |  |  |  |  |  |  |  |  |  |
|  |  |  |  | **F273L** |  |  |  |  |  |  |  |  |  |  |
| 186 | orf19.5210 | *XBP1* | 1734 | A1611G | C1650T | A1672G |  |  |  |  |  |  |  |  |
|  |  |  |  | **I537M** |  | **I558V** |  |  |  |  |  |  |  |  |
| 187 | orf19.3193 | *FCR3* | 1200 |  |  |  |  |  |  |  |  |  |  |  |
| 188 | orf19.175 |  | 1551 | G633A | A645G |  |  |  |  |  |  |  |  |  |
| 189 | orf19.255 | *ZCF1* | 2517 | C1344T | A1455T | A1572G | T1578C | A1594G | G1680A | C1689T | T1692C | A1696G | T1725C |  |
|  |  |  |  |  |  |  |  | **I532V** |  |  |  | **T566A** |  |  |
|  |  |  |  | A1746G | A1878G |  |  |  |  |  |  |  |  |  |
| 190 | orf19.2280 | *ZCF10* | 2748 | C390T | A425G | A436T | C449T | G589A | G607A | C2693T |  |  |  | GCAGCAACAGCAGCAACA589-606Δ |
|  |  |  |  |  | **Q142R** | **T146S** | **T150I** | **A197T** | **A203T** | **S898L** |  |  |  | **AATAAT197-202Δ** |
| 191 | orf19.2423 | *ZCF11* | 1785 | C1035G | G1038C | A1041G | G1521A | G1689T |  |  |  |  |  |  |
|  |  |  |  |  | **E313D** |  |  |  |  |  |  |  |  |  |
| 192 | orf19.2623 | *ECM22* | 2583 | C1116T |  |  |  |  |  |  |  |  |  |  |
| 193 | orf19.2646 | *ZCF13* | 3729 | G3141A | G3149A | G3153T | C3258T | G3273A | C3345T | C3527T | G3550A |  |  |  |
|  |  |  |  |  | **G1050E** |  |  |  |  | **T1176I** | **V1184I** |  |  |  |
| 194 | orf19.2647 | *ZCF14* | 1581 | A1314G | T1337A | A1388G | A1461G |  |  |  |  |  |  | TCGCAT1441-1446Δ |
|  |  |  |  |  | **M446K** | **N463S** |  |  |  |  |  |  |  | **SH483-484Δ** |
| 195 | orf19.2753 | *ZCF15* | 2835 |  |  |  |  |  |  |  |  |  |  |  |
| 196 | orf19.2808 | *ZCF16* | 3237 | A35C | G296A | A414T | T1155C | C1198T | T2655A | G2658T | A3018G |  |  |  |
|  |  |  |  | **N12T** | **S99N** |  |  |  | **D885E** | **E886D** |  |  |  |  |
| 197 | orf19.3305 | *ZCF17* | 2217 | G521A |  |  |  |  |  |  |  |  |  |  |
|  |  |  |  | **S174N** |  |  |  |  |  |  |  |  |  |  |
| 198 | orf19.3405 | *ZCF18* | 1593 |  |  |  |  |  |  |  |  |  |  |  |
| 199 | orf19.3876 | *ZCF19* | 1860 | C1443T | A1495G |  |  |  |  |  |  |  |  |  |
|  |  |  |  |  | **I499V** |  |  |  |  |  |  |  |  |  |
| 200 | orf19.431 | *ZCF2* | 2328 | C1029T | G1074A | A1947G |  |  |  |  |  |  |  |  |
| 201 | orf19.4145 | *ZCF20* | 3258 | C1889T | C1890T | G1986A | T2055C | T2109C | T2163C | G2190A | A2229G | A2253C | C2274T |  |
|  |  |  |  | **T630I** | **T630I** |  |  |  |  |  |  | **Q751H** |  |  |
|  |  |  |  | T2275C | C2307G | G2391A | G2461A | C2472T | T2502C | C2517T | T2577G | C2600A | T2634A |  |
|  |  |  |  |  | **I769M** |  | **V821I** |  |  |  |  | **T867N** |  |  |
|  |  |  |  | C2661T | C2730T | G2801C |  |  |  |  |  |  |  |  |
|  |  |  |  |  |  | **R934P** |  |  |  |  |  |  |  |  |
| 202 | orf19.4166 | *ZCF21* | 1884 | G75A | A396G | G715A | C906T | A1021G |  |  |  |  |  | ACTACT202-207Δ |
|  |  |  |  |  |  | **A239T** |  | **T341A** |  |  |  |  |  | **TT68-69Δ** |
| 203 | orf19.4251 | *ZCF22* | 2100 | G93A | A171T | A457G | G514A | A540G | T594A | T630C | G648A | A652G | T736C |  |
|  |  |  |  |  |  | **K153E** | **V172I** | **L180S** |  |  |  | **T218A** | **Y246H** |  |
|  |  |  |  | C804T | C821T | A953C | A954G | C999T | C1026G | A1077T | T1254C | G1301A | G1416A |  |
|  |  |  |  |  | **A274V** | **E318A** | **E318A** |  |  |  |  | **R434K** |  |  |
|  |  |  |  | C1473T | C1521T | G1969A |  |  |  |  |  |  |  |  |
|  |  |  |  |  |  | **D657N** |  |  |  |  |  |  |  |  |
| 204 | orf19.4450 | *ZCF23* | 1701 | G57C | A187G | G592A |  |  |  |  |  |  |  |  |
|  |  |  |  |  | **S63G** | **E198K** |  |  |  |  |  |  |  |  |
| 205 | orf19.4524 | *ZCF24* | 2184 | T144C | G186A | G207C | G286A | C1614T | G1653A | G1656A | A2061C | T2109C |  |  |
|  |  |  |  |  |  |  | **A70T** |  |  |  |  |  |  |  |
| 206 | orf19.4568 | *ZCF25* | 2418 |  |  |  |  |  |  |  |  |  |  |  |
| 207 | orf19.4573 | *ZCF26* | 2493 |  |  |  |  |  |  |  |  |  |  |  |
| 208 | orf19.4649 | *ZCF27* | 3573 | A1513G | G1954A |  |  |  |  |  |  |  |  | CAACAG223-228Δ |
|  |  |  |  | **N505D** | **A652T** |  |  |  |  |  |  |  |  | **QQ75-76Δ** |
|  |  |  |  |  |  |  |  |  |  |  |  |  |  | CCAGTACAACAA598-609Δ |
|  |  |  |  |  |  |  |  |  |  |  |  |  |  | **PVQQ200-203Δ** |
| 209 | orf19.4767 | *ZCF28* | 741 |  |  |  |  |  |  |  |  |  |  |  |
| 210 | orf19.5133 | *ZCF29* | 3318 | G1738A | T3289C |  |  |  |  |  |  |  |  |  |
|  |  |  |  | **V580I** | **F1097L** |  |  |  |  |  |  |  |  |  |
| 211 | orf19.1168 | *ZCF3* | 588 | G51A | T87C | G93C | G312A |  |  |  |  |  |  |  |
| 212 | orf19.5251 | *ZCF30* | 2400 |  |  |  |  |  |  |  |  |  |  |  |
| 213 | orf19.5924 | *ZCF31* | 3393 |  |  |  |  |  |  |  |  |  |  |  |
| 214 | orf19.5940 | *ZCF32* | 2151 |  |  |  |  |  |  |  |  |  |  |  |
| 215 | orf19.5992 | *WOR2* | 1341 |  |  |  |  |  |  |  |  |  |  |  |
| 216 | orf19.6182 | *MRR2* | 2133 |  |  |  |  |  |  |  |  |  |  |  |
| 217 | orf19.7371 | *ZCF35* | 3309 |  |  |  |  |  |  |  |  |  |  |  |
| 218 | orf19.7372 | *MRR1* | 3327 |  |  |  |  |  |  |  |  |  |  |  |
| 219 | orf19.7381 | *AHR1* | 1875 |  |  |  |  |  |  |  |  |  |  |  |
| 220 | orf19.7518 | *ZCF38* | 1749 |  |  |  |  |  |  |  |  |  |  |  |
| 221 | orf19.7583 | *ZCF39* | 2046 |  |  |  |  |  |  |  |  |  |  |  |
| 222 | orf19.1227 | *ZCF4* | 594 |  |  |  |  |  |  |  |  |  |  | ins177ACC |
|  |  |  |  |  |  |  |  |  |  |  |  |  |  | **ins59T** |
| 223 | orf19.1255 | *ZCF5* | 2916 | A1320G | G1362A | A1956G | T1959C |  |  |  |  |  |  | CAG964-966Δ |
|  |  |  |  |  |  |  |  |  |  |  |  |  |  | **Q322Δ** |
| 224 | orf19.1497 | *ZCF6* | 1110 |  |  |  |  |  |  |  |  |  |  | AGC388-390Δ |
|  |  |  |  |  |  |  |  |  |  |  |  |  |  | **S130Δ** |
| 225 | orf19.1685 | *ZCF7* | 1347 | A660G | G1205T | A1257T | G1274T |  |  |  |  |  |  | FS from +1306 |
|  |  |  |  |  | **G402V** | **K419N** | **G425V** |  |  |  |  |  |  | **CHCYHC436-441FIVPP** |
| 226 | orf19.1718 | *ZCF8* | 2748 | T1117A |  |  |  |  |  |  |  |  |  |  |
|  |  |  |  | **W373R** |  |  |  |  |  |  |  |  |  |  |
| 227 | orf19.2077 | *ZCF9* | 2505 | A258G | G294T | G348A | A385G |  |  |  |  |  |  |  |
|  |  |  |  |  | **E98D** |  | **I129V** |  |  |  |  |  |  |  |
| 228 | orf19.3187 | *ZNC1* | 2769 |  |  |  |  |  |  |  |  |  |  |  |
| 229 | orf19.3300 | *ZPR1* | 1530 |  |  |  |  |  |  |  |  |  |  |  |
| 230 | orf19.274 | *TFC4* | 3180 | G211A | C294T | G377A | G379A |  |  |  |  |  |  | ins111GAGGAA |
|  |  |  |  | **D71N** |  | **G126D** | **G127R** |  |  |  |  |  |  | **ins37EE** |
| 231 | orf19.470 | *TAF12L* | 2226 | T1133C | C1321T |  |  |  |  |  |  |  |  | A1146-A1172Δ |
|  |  |  |  | **V378A** | **P441S** |  |  |  |  |  |  |  |  | **FMGNLQQQQ383-391Δ** |
| 232 | orf19.536 |  | 2394 | G88C | A387T | A417T | A435G |  |  |  |  |  |  | CAA142-144Δ |
|  |  |  |  | **E30Q** |  |  |  |  |  |  |  |  |  | **Q48Δ** |
| 233 | orf19.567 | *TFB3* | 1143 | T969C |  |  |  |  |  |  |  |  |  |  |
| 234 | orf19.735 |  | 1467 |  |  |  |  |  |  |  |  |  |  |  |
| 235 | orf19.736 | *SRB8* | 1671 | C834T | A1269G |  |  |  |  |  |  |  |  |  |
| 236 | orf19.794 | *SSN3* | 1815 | G186A | G208A | T216C | T264A | T423C | C513T | A543T | T567C | C585T | G657A | AACAATAACAAC1636-1647Δ |
|  |  |  |  |  | **A70T** |  |  |  |  |  |  |  |  | **NNNN546-549Δ** |
|  |  |  |  | A702G | T750C | C1593T |  |  |  |  |  |  |  |  |
| 237 | orf19.798 | *TAF14* | 711 |  |  |  |  |  |  |  |  |  |  |  |
| 238 | orf19.861 | *CAP4* | 531 |  |  |  |  |  |  |  |  |  |  |  |
| 239 | orf19.2528 |  | 1989 |  |  |  |  |  |  |  |  |  |  |  |
| 240 | orf19.2612 |  | 753 |  |  |  |  |  |  |  |  |  |  |  |
| 241 | orf19.2674 |  | 1029 |  |  |  |  |  |  |  |  |  |  |  |
| 242 | orf19.2682 |  | 828 | T93C | A588G | C639T | T771C | C798T |  |  |  |  |  |  |
| 243 | orf19.2693 | *GST2* | 660 |  |  |  |  |  |  |  |  |  |  |  |
| 244 | orf19.2736 | *HFL2* | 441 | A294T | C297T | C324T | T336C |  |  |  |  |  |  |  |
| 245 | orf19.2857 |  | 2532 | A417G | T821A | T900C | A999G | G1242A |  |  |  |  |  |  |
|  |  |  |  |  | **I274N** |  |  |  |  |  |  |  |  |  |
| 246 | orf19.2961 |  | 1194 | T976C |  |  |  |  |  |  |  |  |  |  |
| 247 | orf19.3018 | *SPP1* | 1221 |  |  |  |  |  |  |  |  |  |  |  |
| 248 | orf19.3088 |  | 540 | T84C |  |  |  |  |  |  |  |  |  |  |
| 249 | orf19.3121 | *GST1* | 738 |  |  |  |  |  |  |  |  |  |  |  |
| 250 | orf19.3242 | *TAF10* | 735 |  |  |  |  |  |  |  |  |  |  |  |
| 251 | orf19.684 |  | 1548 | C123T | A717G | C745T | T876A | C903T | C1092T |  |  |  |  |  |
|  |  |  |  |  |  | **P249S** |  |  |  |  |  |  |  |  |
| 252 | orf19.3407 | *RAD18* | 1137 | T963C |  |  |  |  |  |  |  |  |  |  |
| 253 | orf19.3683 | *AGE3* | 1128 | G829A |  |  |  |  |  |  |  |  |  | ATAATAATAATA661-672Δ |
|  |  |  |  | **E277K** |  |  |  |  |  |  |  |  |  | **NNNN221-224Δ** |
| 254 | orf19.3835 |  | 1959 |  |  |  |  |  |  |  |  |  |  |  |
| 255 | orf19.3928 |  | 1143 |  |  |  |  |  |  |  |  |  |  |  |
| 256 | orf19.3969 | *SFL2* | 2133 | A588G | A792G |  |  |  |  |  |  |  |  | AAC301-303Δ |
|  |  |  |  |  |  |  |  |  |  |  |  |  |  | **N101Δ** |
|  |  |  |  |  |  |  |  |  |  |  |  |  |  | GCACCACCA688-696Δ |
|  |  |  |  |  |  |  |  |  |  |  |  |  |  | **APP230-232Δ** |
| 257 | orf19.4194 |  | 1092 |  |  |  |  |  |  |  |  |  |  |  |
| 258 | orf19.4295 | *HIR2* | 3054 | A142T | C474T | G558T | T573C | G623A | A808G | T2082C |  |  |  |  |
|  |  |  |  | **T48S** |  | **L186F** |  | **S208N** | **I270V** |  |  |  |  |  |
| 259 | orf19.4342 | *SUT1* | 1083 | G75A | C226T | A252T | C466T | C572T | G694A | C714T | G776A | A807G | C1020T | ins225AATACTAATACTACT |
|  |  |  |  |  | **H76Y** |  | **H156Y** | **A191V** | **V232I** |  | **S259N** |  |  | **ins75NTNTT** |
|  |  |  |  |  |  |  |  |  |  |  |  |  |  | CCT1009-1011Δ |
|  |  |  |  |  |  |  |  |  |  |  |  |  |  | **P337Δ** |
| 260 | orf19.4388 |  | 1095 | G282A | T309C |  |  |  |  |  |  |  |  |  |
| 261 | orf19.4882 |  | 855 | G243C | A348G | G786A |  |  |  |  |  |  |  |  |

| 262 | orf19.4972 | *OFI1* | 1947 | G900A | G1003A |  |  |  |  |  |  |  |  |  |
| --- | --- | --- | --- | --- | --- | --- | --- | --- | --- | --- | --- | --- | --- | --- |
|  |  |  |  |  | **D335N** |  |  |  |  |  |  |  |  |  |
| 263 | orf19.4998 | *ROB1* | 2091 |  |  |  |  |  |  |  |  |  |  |  |
| 264 | orf19.5026 | *ZMS1* | 3534 |  |  |  |  |  |  |  |  |  |  |  |
| 265 | orf19.5068 | *IRE1* | 3666 | G628A | A1759G | T1811C | A1812T | C1822T | A1968G | A2139C | T2151C | C2202G |  | CAA124-126Δ |
|  |  |  |  | **A210T** | **I587V** | **I604T** | **I604T** | **P608S** |  |  |  | **D734E** |  | **Q42Δ** |
|  |  |  |  |  |  |  |  |  |  |  |  |  |  | GGCAAT2971-2976Δ |
|  |  |  |  |  |  |  |  |  |  |  |  |  |  | **GN991-992Δ** |
| 266 | orf19.5297 |  | 1893 |  |  |  |  |  |  |  |  |  |  |  |
| 267 | orf19.5388 | *SWD1* | 1344 |  |  |  |  |  |  |  |  |  |  |  |
| 268 | orf19.5846 |  | 1485 | G237A | C1053T |  |  |  |  |  |  |  |  |  |
| 269 | orf19.5853 |  | 531 |  |  |  |  |  |  |  |  |  |  |  |
| 270 | orf19.5871 | *SNF5* | 2091 | C556T | T621C | A809C | C1638T |  |  |  |  |  |  |  |
|  |  |  |  | **R186C** |  | **K270T** |  |  |  |  |  |  |  |  |
| 271 | orf19.5953 | *SFP1* | 1338 |  |  |  |  |  |  |  |  |  |  |  |
| 272 | orf19.5975 | *TRY4* | 861 |  |  |  |  |  |  |  |  |  |  |  |
| 273 | orf19.6102 | *RCA1* | 852 |  |  |  |  |  |  |  |  |  |  |  |
| 274 | orf19.6239 | *KIN28* | 1032 | A528G | C549T | T768C | G948A | A987G |  |  |  |  |  |  |
| 275 | orf19.6694 |  | 1980 |  |  |  |  |  |  |  |  |  |  |  |
| 276 | orf19.6719 | *HIR3* | 4796 |  |  |  |  |  |  |  |  |  |  |  |
| 277 | orf19.6781 | *ZFU2* | 1104 | T783C |  |  |  |  |  |  |  |  |  |  |
| 278 | orf19.6820 | *TAF12* | 1548 |  |  |  |  |  |  |  |  |  |  |  |
| 279 | orf19.6845 |  | 1440 |  |  |  |  |  |  |  |  |  |  |  |
| 280 | orf19.6850 |  | 1734 | A240G |  |  |  |  |  |  |  |  |  |  |
| 281 | orf19.6874 |  | 1824 | C984T | C1715T |  |  |  |  |  |  |  |  |  |
|  |  |  |  |  | **A572V** |  |  |  |  |  |  |  |  |  |
| 282 | orf19.6923 |  | 1032 |  |  |  |  |  |  |  |  |  |  |  |
| 283 | orf19.1007 | *KCS1* | 1059 | C291T | A477G | C555T | C885T |  |  |  |  |  |  |  |
| 284 | orf19.1111 |  | 681 |  |  |  |  |  |  |  |  |  |  | CCACCACCACCACAACAA121-138Δ |
|  |  |  |  |  |  |  |  |  |  |  |  |  |  | **PPPPQQ41-46Δ** |
| 285 | orf19.1178 |  | 606 |  |  |  |  |  |  |  |  |  |  |  |
| 286 | orf19.1228 | *HAP2* | 1095 |  |  |  |  |  |  |  |  |  |  |  |

| 287 | orf19.1496 |  | 1356 | T129C | T168C | T417C | T777C | G822A | C1241T |  |  |  |  | ins1026ACT |
| --- | --- | --- | --- | --- | --- | --- | --- | --- | --- | --- | --- | --- | --- | --- |
|  |  |  |  |  |  |  |  |  | **A414V** |  |  |  |  | **ins342T** |
|  |  |  |  |  |  |  |  |  |  |  |  |  |  | CCA1114-1116Δ |
|  |  |  |  |  |  |  |  |  |  |  |  |  |  | **P372Δ** |
| 288 | orf19.1451 | *SRB9* | 4998 | A1689G | C3582T | T3696C |  |  |  |  |  |  |  |  |
| 289 | orf19.1476 |  | 1632 | T1361C |  |  |  |  |  |  |  |  |  |  |
|  |  |  |  | **V454A** |  |  |  |  |  |  |  |  |  |  |
| 290 | orf19.1526 | *SNF2* | 5079 | A225G | A243G | A354G | G730A | A762G | T1902A | G1904A | A2237C | C3330T | A4065G | CAACAACAACAG190-201Δ |
|  |  |  |  |  |  |  | **G244S** |  |  | **S635N** | **E746A** |  |  | **QQQQ64-67Δ** |
|  |  |  |  | A4071G | C4113T | A4193G |  |  |  |  |  |  |  | ins534CAACAACAACAACAACAA |
|  |  |  |  |  |  | **D1398G** |  |  |  |  |  |  |  | **ins178QQQQQQ** |
| 291 | orf19.1528 |  | 1056 |  |  |  |  |  |  |  |  |  |  |  |
| 292 | orf19.1565 |  | 1842 | C1032T |  |  |  |  |  |  |  |  |  |  |
| 293 | orf19.1574 |  | 1770 | G649C | C1530A | T1548A | T1686C |  |  |  |  |  |  | ins1713AAT |
|  |  |  |  | **E217Q** | **D510E** | **D516E** |  |  |  |  |  |  |  | **ins571N** |
| 294 | orf19.1577 |  | 1329 |  |  |  |  |  |  |  |  |  |  |  |
| 295 | orf19.1729 |  | 1008 |  |  |  |  |  |  |  |  |  |  |  |
| 296 | orf19.1757 | *MET32* | 1755 | T435C | A755C | A1077G | A1156G |  |  |  |  |  |  | ins1011ACT |
|  |  |  |  |  | **K252T** |  | **T386A** |  |  |  |  |  |  | **ins337T** |
| 297 | orf19.1826 | *MDM34* | 1869 | T113C | G129A | G258A | C818T | T867C | C1399T | T1419C | A1467G | T1524C | T1533A |  |
|  |  |  |  | **L38S** |  |  | **A273V** |  | **H467Y** |  |  |  |  |  |
| 298 | orf19.1864 |  | 2160 |  |  |  |  |  |  |  |  |  |  |  |
| 299 | orf19.2064 |  | 3324 | C1539T | A1586G | T1594G | C1843A | T1980C | T3068C |  |  |  |  |  |
|  |  |  |  |  |  | **S532A** | **P615T** |  | **I1023T** |  |  |  |  |  |
| 300 | orf19.2099 | *HIR1* | 2562 | G141A | A142G | T435C |  |  |  |  |  |  |  |  |
|  |  |  |  |  | **N48D** |  |  |  |  |  |  |  |  |  |
| 301 | orf19.2111 |  | 1146 | C234T | G240A | G332A | C417T | C738T | G768A | A838G |  |  |  |  |
|  |  |  |  |  |  | **R111K** |  |  |  | **T280A** |  |  |  |  |
| 302 | orf19.2135 | *TSM1* | 4278 | T582C | A606G | G611A | G1443A | T1468C | G1515A | C1836T | A1977G | G2099A | C3348T |  |
|  |  |  |  |  |  | **S204N** |  |  |  |  |  |  |  |  |
| 303 | orf19.2236 | *FHL1* | 3459 | T756C | C840T | G885A | C898T | T949C | G1045A | A2115G | T2851C | C2909A | G2943C | ins1002ACATCATCGTCGTCACCAACT |
|  |  |  |  |  |  |  | **P299S** | **S317P** | **A349T** |  | **Y951D** | **A970E** |  | **ins334TSSSSPT** |
| 304 | orf19.2260 |  | 1089 | C975G |  |  |  |  |  |  |  |  |  |  |
| 305 | orf19.2272 | *AFT2* | 2385 | T768C | A971C | A981G | G1104T | A1483G | A1484C | T1883C | A1917G | A1920G |  | GGTCAAAATCAA1489-1500Δ |
|  |  |  |  |  | **N324T** |  | **L368F** | **N499A** | **N499A** | **L628S** |  |  |  | **GQNQ497-500Δ** |
| 306 | orf19.2393 |  | 705 | T64C | A68T |  |  |  |  |  |  |  |  |  |
|  |  |  |  |  | **Y23F** |  |  |  |  |  |  |  |  |  |
| 307 | orf19.2399 |  | 1746 |  |  |  |  |  |  |  |  |  |  |  |
| 308 | orf19.2458 | *SIP5* | 1542 |  |  |  |  |  |  |  |  |  |  |  |
| 309 | orf19.653 |  | 327 |  |  |  |  |  |  |  |  |  |  |  |
| 310 | orf19.1129 |  | 588 | C135T |  |  |  |  |  |  |  |  |  |  |
| 311 | orf19.1694 |  | 1581 | C700A | T1095C | G1355A |  |  |  |  |  |  |  |  |
|  |  |  |  | **P234T** |  | **R452K** |  |  |  |  |  |  |  |  |
| 312 | orf19.4125 | *PZF1* | 1239 | G126A |  |  |  |  |  |  |  |  |  |  |
| 313 | orf19.6921 |  | 1821 |  |  |  |  |  |  |  |  |  |  |  |
| 314 | orf19.7098 |  | 645 | T450C |  |  |  |  |  |  |  |  |  |  |
| 315 | orf19.4433 | *CPH1* | 1965 | G1122A | A1164G | G1353A | A1528G | T1880A |  |  |  |  |  | CAACAG1732-1737Δ |
|  |  |  |  |  |  |  | **T510A** | **V627D** |  |  |  |  |  | **QQ578-579Δ** |
| 316 | orf19.7454 | *TAF60* | 1560 |  |  |  |  |  |  |  |  |  |  |  |
| 317 | orf19.1751 | *SPT23* | 2232 | T1224C | C1254T | T1284C | C1572T | C1619T | A1733G | C1763T |  |  |  | AATAAT1603-1608Δ |
|  |  |  |  |  |  |  |  | **T540I** | **N578S** | **S588F** |  |  |  | **NN535-536Δ** |
|  |  |  |  |  |  |  |  |  |  |  |  |  |  | ins1710GGTGCTGCT |
|  |  |  |  |  |  |  |  |  |  |  |  |  |  | **ins570GAA** |
| 318 | orf19.4884 | *WOR1* | 2358 |  |  |  |  |  |  |  |  |  |  |  |
| 319 | orf19.391 | *UPC2*^G648D^ | 2139 | T1338C | C1392T | C1410A | C1539T |  |  |  |  |  |  |  |
| 320 | orf19.7372 | *MRR1*^P683S^ | 3327 |  |  |  |  |  |  |  |  |  |  |  |
| 321 | orf19.7539 | *INO2* | 1140 |  |  |  |  |  |  |  |  |  |  |  |
| 322 | orf19.837.1 | *INO4* | 477 |  |  |  |  |  |  |  |  |  |  |  |
| 323 | orf19.1623 | *CAP1*^ΔC333^ | 1002 | C399A | A420G | T432C | T465C | A552G | T669C | A672G | T681G | C783T | C972T |  |
|  |  |  |  |  | **I140M** |  |  |  |  |  |  |  |  |  |
| 324 | orf19.1604 | *RHA1* | 3205 | C320G | T355G | C388A | A398C | C411A | C414A | C417A | C420A | C433A | A2398G |  |
|  |  |  |  | **Q29E** | **N40K** | **D51E** | **K55Q** | **T59N** | **T60K** | **T61N** | **T62N** | **F66L** |  |  |
|  |  |  |  | A2434G | C2885A | C2890A | G2930A | T2943A | G2952A | G2957A |  |  |  |  |
|  |  |  |  |  | **Q884K** | **D885E** | **D899N** | **I903K** | **R906K** | **V908I** |  |  |  |  |
| 325 | orf19.4056 | *BRG1* | 1272 | A125G | A283T | A834G | G1010A |  |  |  |  |  |  |  |
|  |  |  |  | **N42S** | **T95S** |  | **R337K** |  |  |  |  |  |  |  |
| 326 | orf19.8420 | *TBF1* | 2661 | G2537A |  |  |  |  |  |  |  |  |  |  |
|  |  |  |  | **S846N** |  |  |  |  |  |  |  |  |  |  |
| 327 | orf19.3188 | *TAC1*^G980E^ | 2946 | T139A | T140A | T1257C | T1305C | T1743C | A2202G | T2214C | T2316A | G2326A | G2485C |  |
|  |  |  |  | **L47K** | **L47K** |  |  |  |  |  | **N772K** | **D776N** | **E829Q** |  |

| 328 | orf19.6121 | *MNL1* | 2718 | C1893T | T2174C | C2697T |  |  |  |  |  |  |  |  |
| --- | --- | --- | --- | --- | --- | --- | --- | --- | --- | --- | --- | --- | --- | --- |
|  |  |  |  |  | **L725S** |  |  |  |  |  |  |  |  |  |
| 329 | orf19.4288 | *CTA7* | 2505 | T1188C | T1347C |  |  |  |  |  |  |  |  |  |
| 330 | orf19.6203 | *PUT3* | 2937 | C63T | A117G | T594C | A624G | C681T |  |  |  |  |  |  |
| 331 | orf19.6038 | *UGA32* | 1752 |  |  |  |  |  |  |  |  |  |  |  |
| 332 | orf19.4046 |  | 462 | T90A |  |  |  |  |  |  |  |  |  |  |
| 333 | orf19.166 | *ASG1* | 2973 | C2093T | A2442T | T2747A | T2750A | T2753A | G2799T | G2804A | A2860C | A2931G |  |  |
|  |  |  |  | **S698F** | **L814F** | **I916N** | **I917N** | **I918N** |  | **G935E** | **I954L** |  |  |  |
| 334 | orf19.6888 |  | 1439 | T924C | T935G | G949A | A1055G |  |  |  |  |  |  |  |
|  |  |  |  | **F272L** |  | **R280K** |  |  |  |  |  |  |  |  |
| 335 | orf19.1084 | *CDC39* | 6051 | G217A | G423A | C1278G | C1332T | G1425T | G1689A | C1899T | T1941C | A4834G | C5640T | CAGCAACAGCAACAG610-624Δ |
|  |  |  |  | **A73T** |  |  |  |  |  |  |  | **K1612E** |  | **QQQQQ204-208Δ** |
|  |  |  |  | A5871T |  |  |  |  |  |  |  |  |  |  |
| 336 | orf19.2545 | *DOT6* | 1803 | T352A | A376G | T483A | C531T | A640C | G662A | C717A | T743C | A756G | C1182A |  |
|  |  |  |  | **S118T** | **T126A** |  |  | **N214H** | **S221N** |  | **V248A** |  |  |  |
|  |  |  |  | T1731C | G1800C |  |  |  |  |  |  |  |  |  |
|  |  |  |  |  | **K600N** |  |  |  |  |  |  |  |  |  |
| 337 | orf19.2847 |  | 1788 | T170C | T180C | G341A | T519A | A523C | G612A | A616G | A675G | G1287A |  |  |
|  |  |  |  | **I57T** |  | **G114E** |  | **E175A** |  | **I206V** |  |  |  |  |
| 338 | orf19.3294 | *MBF1* | 456 | A252G | G255C |  |  |  |  |  |  |  |  |  |
| 339 | orf19.3865 | *RFX1* | 2556 | C8981T | G1200A | A1912G |  |  |  |  |  |  |  |  |
|  |  |  |  |  |  | **N638D** |  |  |  |  |  |  |  |  |
| 340 | orf19.7017 | *YOX1* | 1008 |  |  |  |  |  |  |  |  |  |  |  |
| 341 | orf19.7436 | *AAF1* | 1839 |  |  |  |  |  |  |  |  |  |  |  |
| 342 | orf19.5031 | *SSK1* | 2025 |  |  |  |  |  |  |  |  |  |  |  |
| 343 | orf19.2884 | *CDC68* | 3183 | A1170G | G1457C | G2222A | C2961T | A3089G |  |  |  |  |  |  |
|  |  |  |  |  | **G486A** | **R741K** |  | **D1030G** |  |  |  |  |  |  |

The table lists all polymorphisms found in the cloned genes as compared to assembly 21 of the *C. albicans* genome sequence, except for *EFG1* (clone no. 51), which is identical to orf19.8243. Amino acid substitutions in the encoded proteins are shown in bold below the corresponding nucleotide polymorphisms. All polymorphisms resulting in amino acid changes, except those highlighted in red, were either previously described or verified by sequencing of a second, independent clone. The small in-frame deletion in *MET18* (clone no. 90) was assumed to be a truly existing variant instead of a PCR error. The *TUP1* allele with the T938C and T1373C substitutions (clone no. 177) was obtained from strain WO‑1 and had previously been shown to be functional. The *CPH1*^K652N^ allele (clone no. 24) produced the same phenotype as a later obtained wild-type *CPH1* allele (clone no. 315). The G1793A mutation in *TCC1* resulting in the R598H amino acid exchange (clone no. 147) is presumably the result of a PCR error, but a *TCC1* allele with the correct sequence has not yet been obtained.
