# Supplementary material for: Transcriptional control of C. albicans white-opaque switching and modulation by environmental cues and strain background
Source: mBio. 2025 Apr 9;16(5):e00581-25. doi: 10.1128/mbio.00581-25 (PMC12077150; doi:10.1128/mbio.00581-25)
Supplement: Table S6 — Strains used. [file mbio.00581-25-s0004.docx]

**Table S6. *C. albicans* strains used in this study**

| **Strain number** | **Transcription Factor/Parental** | **Source** | **Description** | | **References** |  |
| --- | --- | --- | --- | --- | --- | --- |
| CAY616 | None (parental) | Bennett Laboratory | SC5314 background strain | |  |  |
| CAY12307 | None (parental) | Morschhauser Laboratory | WO-1 background strain | | Slutsky et al. 1987 |  |
| CAY12481 | Cph1 | Bennett Laboratory | CAY616 SC5314 strain transformed with ApaI/SacII digest of P24 pNim1-Cph1. | | This study |  |
| CAY12482 | Cph1 | Bennett Laboratory | CAY616 SC5314 strain transformed with ApaI/SacII digest of P24 pNim1-Cph1. | | This study |  |
| CAY13164 | Rfx2 | Bennett Laboratory | CAY616 SC5314 strain transformed with ApaI/SacII digest of P40 pNim1-Rfx2. | | This study |  |
| CAY13165 | Rfx2 | Bennett Laboratory | CAY616 SC5314 strain transformed with ApaI/SacII digest of P40 pNim1-Rfx2. | | This study |  |
| CAY12485 | Czf1 | Bennett Laboratory | CAY616 SC5314 strain transformed with ApaI/SacII digest of P49 pNim1-Czf1. | | This study |  |
| CAY12486 | Czf1 | Bennett Laboratory | CAY616 SC5314 strain transformed with ApaI/SacII digest of P49 pNim1-Czf1. | | This study |  |
| CAY13154 | Ndt80 | Bennett Laboratory | CAY616 SC5314 strain transformed with ApaI/SacII digest of P103 pNim1-Ndt80. | | This study |  |
| CAY13155 | Ndt80 | Bennett Laboratory | CAY616 SC5314 strain transformed with ApaI/SacII digest of P103 pNim1-Ndt80. | | This study |  |
| CAY13702 | Rbf1 | Bennett Laboratory | CAY616 SC5314 strain transformed with ApaI/SacII digest of P116 pNim1-Rbf1. | | This study |  |
| CAY13703 | Rbf1 | Bennett Laboratory | CAY616 SC5314 strain transformed with ApaI/SacII digest of P116 pNim1-Rbf1. | | This study |  |
| CAY13162 | Rlm1 | Bennett Laboratory | CAY616 SC5314 strain transformed with ApaI/SacII digest of P121 pNim1-Rlm1. | | This study |  |
| CAY13163 | Rlm1 | Bennett Laboratory | CAY616 SC5314 strain transformed with ApaI/SacII digest of P121 pNim1-Rlm1. | | This study |  |
| CAY13710 | Sfu1 | Bennett Laboratory | CAY616 SC5314 strain transformed with ApaI/SacII digest of P137 pNim1-Sfu1. | | This study |  |
| CAY13711 | Sfu1 | Bennett Laboratory | CAY616 SC5314 strain transformed with ApaI/SacII digest of P137 pNim1-Sfu1. | | This study |  |
| CAY12495 | Zcf21 | Bennett Laboratory | CAY616 SC5314 strain transformed with ApaI/SacII digest of P202 pNim1-Zcf21. | | This study |  |
| CAY12496 | Zcf21 | Bennett Laboratory | CAY616 SC5314 strain transformed with ApaI/SacII digest of P202 pNim1-Zcf21. | | This study |  |
| CAY12497 | Wor2 | Bennett Laboratory | CAY616 SC5314 strain transformed with ApaI/SacII digest of P215 pNim1-Wor2. | | This study |  |
| CAY12498 | Wor2 | Bennett Laboratory | CAY616 SC5314 strain transformed with ApaI/SacII digest of P215 pNim1-Wor2. | | This study |  |
| CAY12499 | Zcf35 | Bennett Laboratory | CAY616 SC5314 strain transformed with ApaI/SacII digest of P217 pNim1-Zcf35. | | This study |  |
| CAY12500 | Zcf35 | Bennett Laboratory | CAY616 SC5314 strain transformed with ApaI/SacII digest of P217 pNim1-Zcf35. | | This study |  |
| CAY12501 | Ofi1 | Bennett Laboratory | CAY616 SC5314 strain transformed with ApaI/SacII digest of P262 pNim1-Ofi1. | | This study |  |
| CAY12502 | Ofi1 | Bennett Laboratory | CAY616 SC5314 strain transformed with ApaI/SacII digest of P262 pNim1-Ofi1. | | This study |  |
| CAY12503 | Wor1 | Bennett Laboratory | CAY616 SC5314 strain transformed with ApaI/SacII digest of P315 pNim1-Wor1. | | This study |  |
| CAY12504 | Wor1 | Bennett Laboratory | CAY616 SC5314 strain transformed with ApaI/SacII digest of P315 pNim1-Wor1. | | This study |  |
| CAY15876 | Wor3 | Bennett Laboratory | CAY616 SC5314 strain transformed with ApaI/SacII digest of RB614 pNim1-Wor3 | | This study |  |
| CAY15877 | Wor3 | Bennett Laboratory | CAY616 SC5314 strain transformed with ApaI/SacII digest of RB614 pNim1-Wor3 | | This study |  |
| CAY15880 | Wor4 | Bennett Laboratory | CAY616 SC5314 strain transformed with ApaI/SacII digest of RB604 pNim1-Wor4 | | This study |  |
| CAY15882 | Wor4 | Bennett Laboratory | CAY616 SC5314 strain transformed with ApaI/SacII digest of RB604 pNim1-Wor4 | | This study |  |
| CAY12505 | Cph1 | Bennett Laboratory | CAY12307 WO-1 strain transformed with a ApaI/SacII digest of P24 pNim1-Cph1. | | This study |  |
| CAY12506 | Cph1 | Bennett Laboratory | CAY12307 WO-1 strain transformed with a ApaI/ SacII digest of P24 pNim1-Cph1. | | This study |  |
| CAY12507 | Rfx2 | Bennett Laboratory | CAY12307 WO-1 strain transformed with ApaI/SacII digest of P40 pNim1-Rfx2. | | This study |  |
| CAY12508 | Rfx2 | Bennett Laboratory | CAY12307 WO-1 strain transformed with ApaI/SacII digest of P40 pNim1-Rfx2. | | This study |  |
| CAY12509 | Czf1 | Bennett Laboratory | CAY12307 WO-1 strain transformed with ApaI/SacII digest of P49 pNim1-Czf1. | | This study |  |
| CAY12510 | Czf1 | Bennett Laboratory | CAY12307 WO-1 strain transformed with ApaI/SacII digest of P49 pNim1-Czf1. | | This study |  |
| CAY13166 | Ndt80 | Bennett Laboratory | CAY12307 WO-1 strain transformed with ApaI/SacII digest of P103 pNim1-Ndt80. | | This study |  |
| CAY13167 | Ndt80 | Bennett Laboratory | CAY12307 WO-1 strain transformed with ApaI/SacII digest of P103 pNim1-Ndt80. | | This study |  |
| CAY13619 | Rbf1 | Bennett Laboratory | CAY12307 WO-1 strain transformed with ApaI/SacII digest of P116 pNim1-Rbf1. | | This study |  |
| CAY13620 | Rbf1 | Bennett Laboratory | CAY12307 WO-1 strain transformed with ApaI/SacII digest of P116 pNim1-Rbf1. | | This study |  |
| CAY13174 | Rlm1 | Bennett Laboratory | CAY12307 WO-1 strain transformed with ApaI/SacII digest of P121 pNim1-Rlm1. | | This study |  |
| CAY13175 | Rlm1 | Bennett Laboratory | CAY12307 WO-1 strain transformed with ApaI/SacII digest of P121 pNim1-Rlm1. | | This study |  |
| CAY13626 | Sfu1 | Bennett Laboratory | CAY12307 WO-1 strain transformed with ApaI/SacII digest of P137 pNim1-Sfu1. | | This study |  |
| CAY13627 | Sfu1 | Bennett Laboratory | CAY12307 WO-1 strain transformed with ApaI/SacII digest of P137 pNim1-Sfu1. | | This study |  |
| CAY12519 | Zcf21 | Bennett Laboratory | CAY12307 WO-1 strain transformed with ApaI/SacII digest of P202 pNim1-Zcf21. | | This study |  |
| CAY12520 | Zcf21 | Bennett Laboratory | CAY12307 WO-1 strain transformed with ApaI/SacII digest of P202 pNim1-Zcf21. | | This study |  |
| CAY12521 | Wor2 | Bennett Laboratory | CAY12307 WO-1 strain transformed with ApaI/SacII digest of P215 pNim1-Wor2. | | This study |  |
| CAY12522 | Wor2 | Bennett Laboratory | CAY12307 WO-1 strain transformed with ApaI/SacII digest of P215 pNim1-Wor2. | | This study |  |
| CAY12523 | Zcf35 | Bennett Laboratory | CAY12307 WO-1 strain transformed with ApaI/SacII digest of P217 pNim1-Zcf35. | | This study |  |
| CAY12524 | Zcf35 | Bennett Laboratory | CAY12307 WO-1 strain transformed with ApaI/SacII digest of P217 pNim1-Zcf35. | | This study |  |
| CAY12525 | Ofi1 | Bennett Laboratory | CAY12307 WO-1 strain transformed with ApaI/SacII digest of P262 pNim1-Ofi1. | | This study |  |
| CAY12526 | Ofi1 | Bennett Laboratory | CAY12307 WO-1 strain transformed with ApaI/SacII digest of P262 pNim1-Ofi1. | | This study |  |
| CAY15884 | Wor3 | Bennett Laboratory | CAY12307 WO-1 strain transformed with ApaI/SacII digest of RB614 pNim1-Wor3 | | This study |  |
| CAY15885 | Wor3 | Bennett Laboratory | CAY12307 WO-1 strain transformed with ApaI/SacII digest of RB614 pNim1-Wor3 | | This study |  |
| CAY15888 | Wor4 | Bennett Laboratory | CAY12307 WO-1 strain transformed with ApaI/SacII digest of RB604 pNim1-Wor4 | | This study |  |
| CAY15889 | Wor4 | Bennett Laboratory | CAY12307 WO-1 strain transformed with ApaI/SacII digest of RB604 pNim1-Wor4 | | This study |  |
| CAY16249 | Ndt80-mNeon | Bennett Laboratory | CAY13154 SC5314 strain transformed with mNeonGreen cassette amplified form pRB2174. | | This study |  |
| CAY16251 | Ndt80-mNeon | Bennett Laboratory | CAY13166 WO-1 strain transformed with mNeonGreen cassette amplified form pRB2174. | | This study |  |
| CAY16253 | Ofi1-mNeon | Bennett Laboratory | CAY12501 SC5314 strain transformed with mNeonGreen cassette amplified form pRB2174 | | This study |  |
| CAY16254 | Ofi1-mNeon | Bennett Laboratory | CAY12525 WO-1 strain transformed with mNeonGreen cassette amplified form pRB2174 | | This study |  |
| ***czf1*∆, *wor1*∆, and *wor2*∆ mutants** | | | | | |  |
| WCZF1M4A and ‑B | WO‑1 | Morschhauser Laboratory | *czf1*∆::*FRT*/*czf1*∆::*FRT* | | Ramírez-Z et al. 2008 |  |
| WWOR1M6A and ‑B | WO‑1 | Morschhauser Laboratory | *wor1*∆::*FRT*/*wor1*∆::*FRT*/*wor1*∆::*FRT* | | Ramírez-Z et al. 2008 |  |
| WWOR2M4A and -B | WO‑1 | Morschhauser Laboratory | *wor2*Δ::*FRT*/*wor2*Δ::*FRT* | | Ramírez-Z et al. 2013 |  |
| ***ndt80****∆* **mutants and complemented strains** | | | | | |  |
| WNDT80M1A and -B | WO‑1 | Morschhauser Laboratory | *NDT80*/*ndt80*∆::*SAT1-FLIP* | | This study |  |
| WNDT80M2A | WNDT80M1A | Morschhauser Laboratory | *NDT80*/*ndt80*∆::*FRT* | | This study |  |
| WNDT80M2B | WNDT80M1B | Morschhauser Laboratory | *NDT80*/*ndt80*∆::*FRT* | | This study |  |
| WNDT80M3A | WNDT80M2A | Morschhauser Laboratory | *ndt80*∆::*FRT*/*ndt80*∆::*SAT1-FLIP* | | This study |  |
| WNDT80M3B | WNDT80M2B | Morschhauser Laboratory | *ndt80*∆::*FRT*/*ndt80*∆::*SAT1-FLIP* | | This study |  |
| WNDT80M4A | WNDT80M3A | Morschhauser Laboratory | *ndt80*∆::*FRT*/*ndt80*∆::*FRT* | | This study |  |
| WNDT80M4B | WNDT80M3B | Morschhauser Laboratory | *ndt80*∆::*FRT*/*ndt80*∆::*FRT* | | This study |  |
| WNDT80MK1A | WNDT80M4A | Morschhauser Laboratory | *ndt80*∆::*FRT*/*NDT80-SAT1-FLIP* | | This study |  |
| WNDT80MK1B | WNDT80M4B | Morschhauser Laboratory | *ndt80*∆::*FRT*/*NDT80-SAT1-FLIP* | | This study |  |
| WNDT80MK2A | WNDT80MK1A | Morschhauser Laboratory | *ndt80*∆::*FRT*/*NDT80-FRT* | | This study |  |
| WNDT80MK2B | WNDT80MK1B | Morschhauser Laboratory | *ndt80*∆::*FRT*/*NDT80-FRT* | | This study |  |
| ***rfx2***∆ **mutants and complemented strains** | | | | | |  |
| WRFX2M1A and -B | WO‑1 | Morschhauser Laboratory | | *RFX2*/*rfx2*∆::*SAT1-FLIP* | This study |  |
| WRFX2M2A | WRFX2M1A | Morschhauser Laboratory | | *RFX2*/*rfx2*∆::*FRT* | This study |  |
| WRFX2M2B | WRFX2M1B | Morschhauser Laboratory | | *RFX2*/*rfx2*∆::*FRT* | This study |  |
| WRFX2M3A | WRFX2M2A | Morschhauser Laboratory | | *rfx2*∆::*FRT*/*rfx2*∆::*SAT1-FLIP* | This study |  |
| WRFX2M3B | WRFX2M2B | Morschhauser Laboratory | | *rfx2*∆::*FRT*/*rfx2*∆::*SAT1-FLIP* | This study |  |
| WRFX2M4A | WRFX2M3A | Morschhauser Laboratory | | *rfx2*∆::*FRT*/*rfx2*∆::*FRT* | This study |  |
| WRFX2M4B | WRFX2M3B | Morschhauser Laboratory | | *rfx2*∆::*FRT*/*rfx2*∆::*FRT* | This study |  |
| WRFX2MK1A | WRFX2M4A | Morschhauser Laboratory | | *rfx2*∆::*FRT*/*RFX2-SAT1-FLIP* | This study |  |
| WRFX2MK1B | WRFX2M4B | Morschhauser Laboratory | | *rfx2*∆::*FRT*/*RFX2-SAT1-FLIP* | This study |  |
| WRFX2MK2A | WRFX2MK1A | Morschhauser Laboratory | | *rfx2*∆::*FRT*/*RFX2-FRT* | This study |  |
| WRFX2MK2B | WRFX2MK1B | Morschhauser Laboratory | | *rfx2*∆::*FRT*/*RFX2-FRT* | This study |  |
| ***rlm1***∆ **mutants** | | | | | |  |
| WRLM1M1A and -B | WO‑1 | Morschhauser Laboratory | | *RLM1*/*rlm1*∆::*SAT1-FLIP* | This study |  |
| WRLM1M2A | WRLM1M1A | Morschhauser Laboratory | | *RLM1*/*rlm1*∆::*FRT* | This study |  |
| WRLM1M2B | WRLM1M1B | Morschhauser Laboratory | | *RLM1*/*rlm1*∆::*FRT* | This study |  |
| WRLM1M3A | WRLM1M2A | Morschhauser Laboratory | | *rlm1*∆::*FRT*/*rlm1*∆::*SAT1-FLIP* | This study |  |
| WRLM1M3B | WRLM1M2B | Morschhauser Laboratory | | *rlm1*∆::*FRT*/*rlm1*∆::*SAT1-FLIP* | This study |  |
| WRLM1M4A | WRLM1M3A | Morschhauser Laboratory | | *rlm1*∆::*FRT*/*rlm1*∆::*FRT* | This study |  |
| WRLM1M4B | WRLM1M3B | Morschhauser Laboratory | | *rlm1*∆::*FRT*/*rlm1*∆::*FRT* | This study |  |
| ***sfu1****∆* **mutants and complemented strains** | | | | | |  |
| WSFU1M1A and -B | WO‑1 | Morschhauser Laboratory | | *SFU1*/*SFU1*/*sfu1*∆::*SAT1-FLIP* | This study |  |
| WSFU1M2A | WSFU1M1A | Morschhauser Laboratory | | *SFU1*/*SFU1*/*sfu1*∆::*FRT* | This study |  |
| WSFU1M2B | WSFU1M1B | Morschhauser Laboratory | | *SFU1*/*SFU1*/*sfu1*∆::*FRT* | This study |  |
| WSFU1M3A | WSFU1M2A | Morschhauser Laboratory | | *SFU1*/*sfu1*∆::*FRT*/*sfu1*∆::*SAT1-FLIP* | This study |  |
| WSFU1M3B | WSFU1M2B | Morschhauser Laboratory | | *SFU1*/*sfu1*∆::*FRT*/*sfu1*∆::*SAT1-FLIP* | This study |  |
| WSFU1M4A | WSFU1M3A | Morschhauser Laboratory | | *SFU1*/*sfu1*∆::*FRT*/*sfu1*∆::*FRT* | This study |  |
| WSFU1M4B | WSFU1M3B | Morschhauser Laboratory | | *SFU1*/*sfu1*∆::*FRT*/*sfu1*∆::*FRT* | This study |  |
| WSFU1M5A | WSFU1M4A | Morschhauser Laboratory | | *sfu1*∆::*FRT*/*sfu1*∆::*FRT*/*sfu1*∆::*SAT1-FLIP* | This study |  |
| WSFU1M5B | WSFU1M4B | Morschhauser Laboratory | | *sfu1*∆::*FRT*/*sfu1*∆::*FRT*/*sfu1*∆::*SAT1-FLIP* | This study |  |
| WSFU1M6A | WSFU1M5A | Morschhauser Laboratory | | *sfu1*∆::*FRT*/*sfu1*∆::*FRT*/*sfu1*∆::*FRT* | This study |  |
| WSFU1M6B | WSFU1M5B | Morschhauser Laboratory | | *sfu1*∆::*FRT*/*sfu1*∆::*FRT*/*sfu1*∆::*FRT* | This study |  |
| WSFU1MK1A | WSFU1M6A | Morschhauser Laboratory | | *sfu1*∆::*FRT*/*sfu1*∆::*FRT*/*SFU1-SAT1-FLIP* | This study |  |
| WSFU1MK1B | WSFU1M6B | Morschhauser Laboratory | | *sfu1*∆::*FRT*/*sfu1*∆::*FRT*/*SFU1-SAT1-FLIP* | This study |  |
| WSFU1MK2A | WSFU1MK1A | Morschhauser Laboratory | | *sfu1*∆::*FRT*/*sfu1*∆::*FRT*/*SFU1-FRT* | This study |  |
| WSFU1MK2B | WSFU1MK1B | Morschhauser Laboratory | | *sfu1*∆::*FRT*/*sfu1*∆::*FRT*/*SFU1-FRT* | This study |  |
| ***zcf21****∆* **mutants and complemented strains** | | | | | |  |
| WZCF21M1A and -B | WO‑1 | Morschhauser Laboratory | | *ZCF21*/*zcf21*∆::*SAT1-FLIP* | This study |  |
| WZCF21M2A | WZCF21M1A | Morschhauser Laboratory | | *ZCF21*/*zcf21*∆::*FRT* | This study |  |
| WZCF21M2B | WZCF21M1B | Morschhauser Laboratory | | *ZCF21*/*zcf21*∆::*FRT* | This study |  |
| WZCF21M3A | WZCF21M2A | Morschhauser Laboratory | | *zcf21*∆::*FRT*/*zcf21*∆::*SAT1-FLIP* | This study |  |
| WZCF21M3B | WZCF21M2B | Morschhauser Laboratory | | *zcf21*∆::*FRT*/*zcf21*∆::*SAT1-FLIP* | This study |  |
| WZCF21M4A | WZCF21M3A | Morschhauser Laboratory | | *zcf21*∆::*FRT*/*zcf21*∆::*FRT* | This study |  |
| WZCF21M4B | WZCF21M3B | Morschhauser Laboratory | | *zcf21*∆::*FRT*/*zcf21*∆::*FRT* | This study |  |
| WZCF21MK1A | WZCF21M4A | Morschhauser Laboratory | | *zcf21*∆::*FRT*/*ZCF21-SAT1-FLIP* | This study |  |
| WZCF21MK1B | WZCF21M4B | Morschhauser Laboratory | | *zcf21*∆::*FRT*/*ZCF21-SAT1-FLIP* | This study |  |
| WZCF21MK2A | WZCF21MK1A | Morschhauser Laboratory | | *zcf21*∆::*FRT*/*ZCF21-FRT* | This study |  |
| WZCF21MK2B | WZCF21MK1B | Morschhauser Laboratory | | *zcf21*∆::*FRT*/*ZCF21-FRT* | This study |  |
| ***zcf35****∆* **mutants and complemented strains** | | | | | |  |
| WZCF35M1A and -B | WO‑1 | Morschhauser Laboratory | | *ZCF35*/*zcf35*∆::*SAT1-FLIP* | This study |  |
| WZCF35M2A | WZCF35M1A | Morschhauser Laboratory | | *ZCF35*/*zcf35*∆::*FRT* | This study |  |
| WZCF35M2B | WZCF35M1B | Morschhauser Laboratory | | *ZCF35*/*zcf35*∆::*FRT* | This study |  |
| WZCF35M3A | WZCF35M2A | Morschhauser Laboratory | | *zcf35*∆::*FRT*/*zcf35*∆::*SAT1-FLIP* | This study |  |
| WZCF35M3B | WZCF35M2B | Morschhauser Laboratory | | *zcf35*∆::*FRT*/*zcf35*∆::*SAT1-FLIP* | This study |  |
| WZCF35M4A | WZCF35M3A | Morschhauser Laboratory | | *zcf35*∆::*FRT*/*zcf35*∆::*FRT* | This study |  |
| WZCF35M4B | WZCF35M3B | Morschhauser Laboratory | | *zcf35*∆::*FRT*/*zcf35*∆::*FRT* | This study |  |
| WZCF35MK1A | WZCF35M4A | Morschhauser Laboratory | | *zcf35*∆::*FRT*/*ZCF35-SAT1-FLIP* | This study |  |
| WZCF35MK1B | WZCF35M4B | Morschhauser Laboratory | | *zcf35*∆::*FRT*/*ZCF35-SAT1-FLIP* | This study |  |
| WZCF35MK2A | WZCF35MK1A | Morschhauser Laboratory | | *zcf35*∆::*FRT*/*ZCF35-FRT* | This study |  |
| WZCF35MK2B | WZCF35MK1B | Morschhauser Laboratory | | *zcf35*∆::*FRT*/*ZCF35-FRT* | This study |  |
| ***ofi1****∆* **mutants** | | | | | |  |
| W4972M1A and -B | WO‑1 | Morschhauser Laboratory | | *OFI1*/ *OFI1*/*ofi1∆*::*SAT1-FLIP* | This study |  |
| W4972M2A | W4972M1A | Morschhauser Laboratory | | *OFI1/ OFI1/ofi1*∆::*FRT* | This study |  |
| W4972M2B | W4972M1B | Morschhauser Laboratory | | *OFI1/OFI1/ofi1∆*::*FRT* | This study |  |
| W4972M3A | W4972M2A | Morschhauser Laboratory | | *OFI1/ofi1*∆::*FRT*/*ofi1∆*::*SAT1-FLIP* | This study |  |
| W4972M3B | W4972M2B | Morschhauser Laboratory | | *OFI1/ofi1*∆::*FRT*/*ofi1*∆::*SAT1-FLIP* | This study |  |
| W4972M4A | W4972M3A | Morschhauser Laboratory | | *OFI1/ ofi1*∆::*FRT*/*ofi1*∆::*FRT* | This study |  |
| W4972M4B | W4972M3B | Morschhauser Laboratory | | *OFI1 ofi1*∆::*FRT*/*ofi1*∆::*FRT* | This study |  |
| W4972M5A | W4972M4A | Morschhauser Laboratory | | *ofi1*∆::*FRT*/*ofi1*∆::*FRT*/*ofi1*∆::*SAT1-FLIP* | This study |  |
| W4972M5B | W4972M4B | Morschhauser Laboratory | | *ofi1*∆::*FRT*/*ofi1*∆::*FRT*/*ofi1*∆::*SAT1-FLIP* | This study |  |
| W4972M6A | W4972M5A | Morschhauser Laboratory | | *ofi1*∆::*FRT*/*ofi1*∆::*FRT*/*ofi1*∆::*FRT* | This study |  |
| W4972M6B | W4972M5B | Morschhauser Laboratory | | *ofi1*∆::*FRT*/*ofi1*∆::*FRT*/*ofi1*∆::*FRT* | This study |  |
| **Strains expressing P*_OP4_-GFP* or P*_OP4_-RFP* reporter fusions in wild-type, *czf1***∆**, *wor1***∆**, and *wor2***∆ **backgrounds** | | | | | |  |
| WOP4G42A | WOP4G41A | Morschhauser Laboratory | | *OP4*/*OP4*/*op4*::P*_OP4_-GFP-FRT* | Sasse et al. 2013 |  |
| WOP4R22A | WOP4R21A | Morschhauser Laboratory | | *OP4*/*OP4*/*op4*::P*_OP4_-RFP-FRT* | Sasse et al. 2013 |  |
| WCZF1M4AOP4G41 | WCZF1M4A | Morschhauser Laboratory | | *czf1*∆::*FRT*/*czf1*∆::*FRT OP4*/*OP4*/*op4*::P*_OP4_-GFP-SAT1-FLIP* | Ramírez-Z et al. 2013 |  |
| WCZF1M4AOP4G42 | WCZF1M4AOP4G41 | Morschhauser Laboratory | | *czf1*∆::*FRT*/*czf1*∆::*FRT OP4*/*OP4*/*op4*::P*_OP4_-GFP-FRT* | Ramírez-Z et al. 2013 |  |
| WCZF1M4BOP4R21 | WCZF1M4B | Morschhauser Laboratory | | *czf1*∆::*FRT*/*czf1*∆::*FRT OP4*/*OP4*/*op4*::P*_OP4_-RFP-SAT1-FLIP* | Ramírez-Z et al. 2013 |  |
| WCZF1M4BOP4R22 | WCZF1M4BOP4R21 | Morschhauser Laboratory | | *czf1*∆::*FRT*/*czf1*∆::*FRT OP4*/*OP4*/*op4*::P*_OP4_-RFP-FRT* | Ramírez-Z et al. 2013 |  |
| WWOR1M6AOP4G41 | WWOR1M6A | Morschhauser Laboratory | | *wor1*∆::*FRT*/*wor1*∆::*FRT*/*wor1*∆::*FRT OP4*/*OP4*/*op4*::P*_OP4_-GFP-SAT1-FLIP* | Ramírez-Z et al. 2013 |  |
| WWOR1M6AOP4G42 | WWOR1M6AOP4G41 | Morschhauser Laboratory | | *wor1*∆::*FRT*/*wor1*∆::*FRT*/*wor1*∆::*FRT OP4*/*OP4*/*op4*::P*_OP4_-GFP-FRT* | Ramírez-Z et al. 2013 |  |
| WWOR1M6BOP4R21 | WWOR1M6B | Morschhauser Laboratory | | *wor1*∆::*FRT*/*wor1*∆::*FRT*/*wor1*∆::*FRT OP4*/*OP4*/*op4*::P*_OP4_-RFP-SAT1-FLIP* | Ramírez-Z et al. 2013 |  |
| WWOR1M6BOP4R22 | WWOR1M6BOP4R21 | Morschhauser Laboratory | | *wor1*∆::*FRT*/*wor1*∆::*FRT*/*wor1*∆::*FRT*  *OP4*/*OP4*/*op4*::P*_OP4_-RFP-FRT* | Ramírez-Z et al. 2013 |  |
| WWOR2M4AOP4G41 | WWOR2M4A | Morschhauser Laboratory | | *wor2*∆::*FRT*/*wor2*∆::*FRT OP4*/*OP4*/*op4*::P*_OP4_-GFP-SAT1-FLIP* | Ramírez-Z et al. 2013 |  |
| WWOR2M4AOP4G42 | WWOR2M4AOP4G41 | Morschhauser Laboratory | | *wor2*∆::*FRT*/*wor2*∆::*FRT OP4*/*OP4*/*op4*::P*_OP4_-GFP-FRT* | Ramírez-Z et al. 2013 |  |
| WWOR2M4BOP4R21 | WWOR2M4B | Morschhauser Laboratory | | *wor2*∆::*FRT*/*wor2*∆::*FRT OP4*/*OP4*/*op4*::P*_OP4_-RFP-SAT1-FLIP* | Ramírez-Z et al. 2013 |  |
| WWOR2M4BOP4R22 | WWOR2M4BOP4R21 | Morschhauser Laboratory | | *wor2*∆::*FRT*/*wor2*∆::*FRT OP4*/*OP4*/*op4*::P*_OP4_-RFP-FRT* | Ramírez-Z et al. 2013 |  |

**References**

1. Slutsky B, Staebell M, Anderson J, Risen L, Pfaller M, et al. (1987) "White-opaque transition": a second high-frequency switching system in *Candida albicans*. J Bacteriol 169: 189-197.

2. Ramírez-Zavala B, Reuß O, Park Y-N, Ohlsen K, Morschhäuser J (2008) Environmental induction of white-opaque switching in *Candida albicans*. PLoS Pathog 4: e1000089.

3. Sasse C, Hasenberg M, Weyler M, Gunzer M, Morschhäuser J (2013) White-opaque switching of *Candida albicans* allows immune evasion in an environment-dependent fashion. Eukaryot Cell 12: 50-58.

4. Ramirez-Zavala B, Weyler M, Gildor T, Schmauch C, Kornitzer D, Arkowitz R, Morschhauser J. 2013. Activation of the Cph1-dependent MAP kinase signaling pathway induces white-opaque switching in *Candida albicans*. PLoS Pathog 9:e1003696
